# Is the myth of left-wing authoritarianism itself a myth?

**DOI:** 10.3389/fpsyg.2022.1041391

**Published:** 2023-02-08

**Authors:** Lucian Gideon Conway III, Alivia Zubrod, Linus Chan, James D. McFarland, Evert Van de Vliert

**Affiliations:** ^1^Department of Psychology, Social Work and Sociology, Grove City College, Grove City, PA, United States; ^2^Department of Psychology and Sociology, Park University, Parkville, MO, United States; ^3^Department of Psychology, University of Montana, Missoula, MT, United States; ^4^Department of Social and Organizational Psychology, University of Groningen, Groningen, Netherlands

**Keywords:** left-wing authoritarianism, right-wing authoritarianism, ideology, political psychology, conservatism

## Abstract

Is left-wing authoritarianism (LWA) closer to a myth or a reality? Twelve studies test the empirical existence and theoretical relevance of LWA. Study 1 reveals that both conservative *and* liberal Americans identify a large number of left-wing authoritarians in their lives. In Study 2, participants explicitly rate items from a recently-developed LWA measure as valid measurements of authoritarianism. Studies 3–11 show that persons who score high on this same LWA scale possess the traits associated with models of authoritarianism: LWA is positively related to threat sensitivity across multiple areas, including general ecological threats (Study 3), COVID disease threat (Study 4), Belief in a Dangerous World (Study 5), and Trump threat (Study 6). Further, high-LWA persons show more support for restrictive political correctness norms (Study 7), rate African-Americans and Jews more negatively (Studies 8–9), and show more cognitive rigidity (Studies 10 and 11). These effects hold when controlling for political ideology and when looking only within liberals, and further are similar in magnitude to comparable effects for right-wing authoritarianism. Study 12 uses the World Values Survey to provide cross-cultural evidence of Left-Wing Authoritarianism around the globe. Taken in total, this large array of triangulating evidence from 12 studies comprised of over 8,000 participants from the U.S. and over 66,000 participants world-wide strongly suggests that left-wing authoritarianism is much closer to a reality than a myth.

## 1. Introduction

The idea that liberals can show rigid adherence to authority figures – known as left-wing authoritarianism (LWA) – has a rocky history in psychology. Indeed, some scholars have expressed extreme skepticism about the validity or real-life viability of the construct, with researchers calling it a *myth* on par with the *Loch Ness Monster*, or suggesting that left-wing authoritarian persons are as *rare as hen’s teeth* (Stone, 1980; Altemeyer, 1996; Jost et al., 2003).

However, others have historically argued that left-wing authoritarianism is a relevant reality deserving of scientific attention (e.g., Eysenck, 1954; Shils, 1954; Rokeach, 1960; Ray, 1983; McFarland et al., 1996; Mullen et al., 2003; Van Hiel et al., 2006). Burgeoning evidence spanning multiple nations, independent research programs, and cultural contexts suggests that LWA exists and has important consequences (McFarland et al., 1992, 1993, 1996; Pentony et al., 2000; Todosijević, 2005; Van Hiel et al., 2006; Todosijević and Enyedi, 2008; De Regt et al., 2011; Frimer et al., 2014; Malka et al., 2017a,b; Manson, 2020). Indeed, the case for LWA has been bolstered by a recent *new wave* of research that has sparked a re-emergence of interest in the construct (e.g., Frimer et al., 2014; Federico et al., 2017; Proch et al., 2018; Vasilopoulos et al., 2018; Wronski et al., 2018; Conway et al., 2018, 2020a, 2021a,b; Conway and McFarland, 2019; Fasce and Avendaño, 2020; Manson, 2020; Costello et al., 2022).

Evidence for LWA to date roughly falls into two categories (see Conway et al., 2021b, for discussion). On the one hand, there is a great deal of cross-cultural evidence from nations with authoritarian left-wing regimes – such as the former Soviet Union. Not only do leaders in those liberal regimes exhibit authoritarian behaviors, but authoritarian measurements in those populaces tend to correlate with support for the left-wing authoritarian government (e.g., McFarland et al., 1992, 1993, 1996; Pentony et al., 2000; Grigoryev et al., 2022). However, as evidence for left-wing authoritarianism, this work provides a proverbial mixed bag because generally the authoritarian scales (that are correlated with left-wing ideological beliefs) contain *right-wing* authoritarianism traits. As a result, authoritarianism in those cultures tends to be a mixture of left- and right-wing authoritarian ideologies, making the pure case for truly left-wing authoritarianism harder to parse from this evidence (for discussion, see Jost et al., 2003; Conway et al., 2021b).

The second category of evidence helps offset some of these ambiguities by directly anchoring left-wing authoritarianism to the classic psychological definition of an authoritarian person (as opposed to authoritarian governance). Developed largely on right-wing persons, this psychological approach conceptualizes authoritarians as having motivations to submit to authority, desire authority figures to punish those who do not, and want those authority figures to enforce group norms (e.g., Altemeyer, 1996; Feldman, 2003; Duckitt et al., 2010; see Conway et al., 2021b, for a summary). This conceptualization suggests that although the underlying authoritarian motivations are similar in different types of authoritarians, it is possible for those motivations to be directed towards different ideological leaders and causes. Whereas RWA measurements focus on submission to conservative positions and leaders, LWA measurements focus on submission to liberal positions and leaders. In the case of LWA, someone scores as a left-wing authoritarian if they endorse authoritarianism *for left wing positions only*. As such, when someone scores high on this sort of left-wing authoritarian questionnaire, it is more difficult to argue “this may be a form of right-wing authoritarianism.” It is thus unsurprising that this type of more direct evidence is largely responsible for the *new wave* of research that has re-invigorated the debate about LWA (e.g., Proch et al., 2018; Conway et al., 2018, 2020b, 2021a,b; Conway and McFarland, 2019; Fasce and Avendaño, 2020; Costello et al., 2022).

This renewed energy for LWA and the growing body of evidence in favor of its validity as a measured psychological construct has caused quite a bit of pushback (e.g., Nilsson and Jost, 2020). Against the backdrop of this skepticism, Costello et al. (2022) developed a new LWA scale and subsequently used this scale to produce an array of evidence that LWA is correlated in expected ways (even when controlling for political ideology) to a whole host of factors, including those related to cognitive rigidity and support for political violence (Costello et al., 2022).

However, this recent array of work does not appear to have altered the skepticism surrounding LWA overly much. Indeed, in an even more recent article, Gries et al. (2022) continue to discuss authoritarianism as existing only on the conservative side of the political spectrum. Further, a quick survey of recently-published papers suggests that authoritarianism is still viewed as an almost exclusively right-wing phenomenon; most papers on the topic implicitly assume that authoritarians exist only on the right, entirely ignoring left-wing authoritarianism (for examples, see Górska et al., 2021; Pazhoohi and Kingstone, 2021; Peresman et al., 2021). As a result, clearly more definitive work is needed to fully evaluate the controversial question of left-wing authoritarianism. Inspired by criticisms of both the empirical phenomenon and the theoretical construct, in the present paper, we present novel evidence using multiple approaches for validating the utility of LWA.

This evidence focuses partly (in Studies 2–11) on a scale that was developed independently from Costello et al.’s (2022) scale. Importantly, the two scales were not only developed independently, they were developed with different approaches and aims. Whereas Costello et al. (2022) purposefully developed an LWA scale without overtly incorporating prior authoritarianism scales, Conway et al. (2018) intentionally modeled their scale after the most widely-used RWA scale (Altemeyer, 1996). These two approaches have complementary strengths and weaknesses. Creating a scale without overtly incorporating other scales might uncover truths missed in past work or avoid potential pitfalls of that work. However, this method does not allow for easy comparisons with prior work. For example, if we want to know the relationship of RWA effects to LWA effects, building an LWA scale independent of past RWA work makes it difficult to know the degree that differences in RWA and LWA are inherent in the two constructs versus due to alternative forms of scale construction. Building a scale directly from prior work ameliorates this concern by purposefully keeping the authoritarian language and semantics constant across the scales and only changing aspects related to ideology. This method is based in basic experimental logic: To the degree that (1) all words related to authoritarianism for both RWA and LWA are face valid measurements of authoritarianism, (2) all words related to authoritarianism are essentially identical for both RWA and LWA, and (3) the only thing varied across scales is the *type of authoritarian submitted to*, we can infer that (4) similarities in effects across scales are reasonably attributed to authoritarianism, whereas (5) differences in effects across scales are reasonably attributed to the type of authority figures.

Because they have complementary strengths and weaknesses, work from these two independent research programs can provide conceptually triangulating evidence. When debates in the field are manifest, independent tests of conceptual ideas are vitally important (see, e.g., Crandall and Sherman, 2016). To that end, in the present paper, we pursue four different aims that all help provide tests of the legitimacy and importance of left-wing authoritarianism. These aims include (1) descriptive evidence from the American populace about their likelihood of knowing high-LWA persons, (2) content validity tests of the Conway et al. (2018) LWA questionnaire revealing that participants explicitly rate the items as measuring authoritarianism, (3) tests showing that this LWA measure is related to substantive domains (perceived threat, restrictive norms, negative outgroup perceptions, and cognitive rigidity) that authoritarianism theory suggests it ought to be related to, and (4) a world-wide survey demonstrating that some countries exhibit higher levels of left-wing authoritarianism (LWA) than right-wing authoritarianism (RWA). This work additionally controls for ideology not only by using standard measures of ideological self-identification (Studies 3–10), but also a specific ideological belief related to the dependent variable (climate change; Study 10) and a more nuanced, two-dimensional measure of social and economic conservative content (Study 11). Finally, we provide summary analyses of comparative LWA/RWA tests (Studies 3–11), within-group tests (Studies 3–11), and a world-wide survey using a widely-accepted authoritarian government questionnaire (Study 12). This array of novel triangulating evidence overwhelmingly suggests that left-wing authoritarianism is a fact rather than a fable.

Below, we first describe the basis for skepticism of LWA research. Against that backdrop, we then discuss four aims designed to overcome this skepticism. Finally, we proceed to the 12 studies demonstrating that LWA is, indeed, realistic and relevant.

### 1.1. Basis of skepticism of left-wing authoritarianism

#### 1.1.1. Altemeyer’s LWA evidence

Historically, much of the skepticism about LWA can be traced to Altemeyer’s work on the construct. After constructing an LWA scale purported to be parallel to his RWA scale, he found almost no evidence of LWA, indeed famously reporting that high-LWA persons were “as rare as hen’s teeth in my samples” (Altemeyer, 1996). This lack of evidence from Altemeyer has often been one of the key arguments cited to suggest that LWA does not exist. For example, in their highly-cited paper on ideology, Jost et al. (2003)’s dismissal of LWA as a construct prominently featured Altemeyer’s research. Indeed, one does not have to read far into Jost et al.’s (2003) classic paper to see how much of their own view of the rigidity of the right is based on Altemeyer’s work. For example (Jost et al., 2003; p. 353):

“Altemeyer (1998) concluded, ‘I have yet to find a single “socialist/Communist type” who scores highly (in absolute terms) on the [Left-Wing Authoritarianism] Scale. Shils may have been right about his era, but the “authoritarian on the left” has been as scarce as hens’ teeth in my samples’ Evidence suggests that dogmatism has been no more useful than the construct of authoritarianism for identifying rigidity of the left …”

This reliance on Altemeyer’s evidence by skeptics of LWA would be rather alarming even if the evidence was particularly compelling, because it is based on only a few samples from one cultural context. However, it is especially troubling because upon closer inspection, Altemeyer’s evidence is itself deeply flawed even in describing that one cultural context. For example, although his LWA questionnaire was intended by his own stated goal to be parallel to his RWA scale, it is clearly not parallel in multiple large-scale ways. Specifically, Altemeyer’s LWA scale adds two highly salient item features not present in the RWA scale.

First, Altemeyer’s LWA scale requires participants who score high on the questionnaire to support a revolution to overthrow the established government. In fact, 20 of the 22 items on Altemeyer’s LWA scale reference a revolutionary movement. For example: “The members of the Establishment deserve to be dealt with harshly, without mercy, when they are finally overthrown.” By contrast, *none* of the items on any of Altemeyer’s RWA scales makes a single reference to violent upheavals overthrowing the establishment. Second, whereas the RWA and LWA scales both use vigorous authoritarian, negative, dogmatic, and punitive language, only the LWA items leave absolutely no doubt that the endorsement of violence is explicitly required to score high on the scale. For example: “The conservative right-wing Establishment will never give up its power peacefully, so a revolutionary movement is justified in using violence to crush it.”

Thus, Altemeyer’s RWA and LWA scales are *not* parallel in very important ways. Indeed, whereas Altemeyer’s RWA scale reads like a measure of general authoritarianism, Altemeyer’s LWA scale reads like a screening instrument for joining a violent revolutionary group that wants to overthrow the government. As a result, the fact that few people scored high on Altemeyer’s LWA scale tells us little about left-wing authoritarianism. Rather, it simply tells us the obvious fact that, whether left-wing or right-wing, few people want to endorse, let alone join, a violent military movement designed to attack and overthrow something else. However, historically, some of academia’s dismissal of LWA was based on this clearly flawed evidence from Altemeyer. What is needed, then, are more truly parallel scales that keep authoritarian language constant while altering only the ideological targets of authoritarianism. In this paper, we evaluate the validity of one such scale: Conway et al.’s (2018) LWA scale.

#### 1.1.2. The double-barreled nature of authoritarianism

A second basis of skepticism in LWA research concerns the fact that Left-Wing Authoritarianism is a *double-barreled* construct. Because it contains both ideological (left-wing) content *and* authoritarian content, it can be a challenge to disentangle the degree that LWA effects are the result of *authoritarianism* or the result of *ideology*. For example, Honeycutt and Jussim (2020) wondered about Conway et al.’s (2018) LWA evidence: “Of course, this problem is itself confounded with the measurement problem—is anyone shocked that conservatives score higher than liberals on a *rightwing* authoritarianism scale, whereas liberals score higher than conservatives, on a *leftwing* authoritarianism scale?” The implication is clear: Because LWA simultaneously measures both ideology and authoritarianism (that is, because it is *both* left-wing *and* authoritarian), how can we be sure that any results we find are truly about *authoritarianism per se*? Perhaps those findings could be explained without considering *authoritarianism* at all.

This perfectly reasonable measurement concern is not a problem specific to LWA measurement: It is a problem likely inherent in *any* authoritarianism measurement. Right-wing authoritarianism also has both ideological content (right-wing) and authoritarianism embedded into the construct. Indeed, no matter what you put in the blank, [blank] authoritarianism will be double-barreled in some sense, because it will have both some content (the “[blank]”) and also authoritarianism built in. And yet this state of things does not invalidate that authoritarianism is an important construct with real-world consequences. The fact that a right-wing authoritarian clings to religious authorities but rejects scientific authorities does not make them less authoritarian. Likewise, the fact that a left-wing authoritarian clings to liberal authorities but rejects conservative ones does not make them less authoritarian.

However, in both cases, it does pose a measurement challenge: How are we to separate the ideological parts from the authoritarian parts? In the case of LWA, how do we separate *liberal non-authoritarians* from *liberal authoritarians*? This is, in fact, one of the primary challenges critics have levied at LWA research (Nilsson and Jost, 2020). One way to solve this problem is by attempting to write an “ideologically neutral” scale that does not make reference to ideological positions at all. While this sounds good on the surface, in practice, it does not work well because participants generally infer specific authoritarian leaders. For example, Nilsson and Jost (2020) recommend Dunwoody and Funke’s (2016) Aggression-Submission-Conventionalism (ASC) scale as “value neutral.” But this recommendation reveals the difficulty with this approach. Evidence suggests the ASC scale is not very ideologically-neutral. For example, the ASC scale is extremely highly correlated with Altemeyer’s RWA scale or scales based on Altemeyer’s scale (for details, see Dunwoody and Funke, 2016). Because the ASC scale is highly correlated with a scale set clearly acknowledged as ideology-laden, it is not clear that it is in fact particularly value-neutral.

Drawing on established methods in social psychology, Conway (2020) offered several solutions to the double-barreled measurement problem inherent in authoritarianism. Here we employ two of these methods (see Supplementary material for more details): (a) First, we use *parallel, ideologically-balanced scales that control for ideology* (Studies 3–11). If scientists want to isolate the “authoritarian” part of “X” Authoritarian, they can statistically control for “X” (see, e.g., Preacher, 2015; Hayes, 2017; Hayes and Rockwood, 2020). Thus, if we aim to isolate the “authoritarian” part of left-wing authoritarianism, we can control for participants’ ideology (“left-wing/right-wing”). (b) Second, we use *parallel, ideologically-balanced scales within the focal group* (Studies 3–11; see Wronski et al., 2018, for an example). If one finds an LWA effect *only within liberal persons*, then this suggests the effect of LWA is driven by authoritarianism and not left-wing ideology. In other words, one way to separate liberal authoritarians from liberal non-authoritarians is to look only at liberals.

As we will see, both methods overwhelmingly support the idea that there is something beyond mere ideology at play here; that *something beyond* is, we believe, best described as *authoritarianism*.

## 2. Equivalent standards for RWA and LWA: Four aims

When approaching any scientific issue, it is important to apply the same standards of evidence on all sides of a discussion (Tetlock, 1994). In the present case, this issue is pertinent in several important ways that correspond to four aims for our work.

### 2.1. Aim 1: Descriptive validity

One of the claims made by critics of LWA work is that there simply are not very many left-wing authoritarians. However, no work that we are aware of attempts to directly compare a populace’s own impressions of the number of left- (versus right-) wing authoritarians in their lives using standards that are equivalent across ideological groups. We do so in Aim 1 here.

### 2.2. Aim 2: Content validity

Altemeyer’s RWA scale – on which Conway et al.’s (2018) LWA scale was based – has historically been, and still is, by *far* the most extensively-used measurement of the right-wing authoritarianism construct. For example, an empirical study (Conway et al., 2018) showed that 79% of the scales from recent research that measured RWA used a version based on Altemeyer’s scale – either Altemeyer’s original RWA scale (62%) or the short version constructed by Zakrisson (2005; 17%). Indeed, even since 2018, Altemeyer’s RWA scale has continued to be widely-used in top journals (including *Journal of Personality and Social Psychology* and *Personality and Social Psychology Bulletin*), and in most cases used in a manner that assumes it is measuring *authoritarianism* and not just ideology (see Conway et al., 2020a).

The scientific consensus thus overwhelmingly favors the conclusion that Altemeyer’s RWA scale measures *authoritarianism* above and beyond ideology. Indeed, it is the past and present scientific standard in the field for measuring authoritarianism, and decades of scientific knowledge about the construct – knowledge often accepted as axiomatic – has been built upon it. And for good reason: As we will see in Study 2, it is an excellent face valid measurement of authoritarianism. Thus in this case the scientific consensus is correct: Altemeyer’s RWA measurement, though not without flaws, is a good measure of general *right-wing* authoritarianism.

Given this, it is important that we apply the same standards of evidence to judging LWA that have been used to arrive at that conclusion for RWA. Consider the case of Conway et al.’s (2018) LWA scale used in many of the present studies. Unlike Altemeyer’s own LWA scale, Conway et al.’s LWA scale mirrors the language of the most-validated and widely used RWA scale. As a result, Conway et al.’s LWA scale possesses high content validity as a measure of authoritarianism.

Indeed, participants who score high on the LWA scale agree that (italicized words are direct quotes from the LWA scale): *Our country needs a mighty leader*, that the leader should *destroy* opponents, that people should *trust the judgment of the proper authorities*, should avoid listening to *noisy rabble rousers in our society who are trying to create doubts in people’s minds*, should *put some tough leaders in power who oppose those values and silence the troublemakers*, should *smash* the beliefs of opponents, that *what our country really needs is a strong, determined leader who will crush the evil*, that society should *strongly punish those* they disagree with, deny that their opponents have a right to *be wherever he or she wants to be*, and support the statement that the country would be better off if certain groups *would just shut up and accept their group’s proper place in society*. These items hit all of the hallmarks of the consensus conceptualization of the *authoritarian* person. For decades, it has been assumed that if people agreed with those statements when the targets of authoritarianism were conservative and the outgroups were liberal, then they were indeed *authoritarians*. Therefore, if people agree with those statements when the targets of authoritarianism are *liberal* and the outgroups are *conservative*, we must – applying the same standard – also agree that they are authoritarians. If we grant that someone saying they want to *put some tough leaders in power who oppose those values and silence the troublemakers* is authoritarian when referring to right-wing leaders, then we also have to grant that someone saying the exact same thing when referring to left-wing leaders is also an authoritarian.

Nonetheless, it is important to empirically test these content validity assumptions. In the present work, we provide one such validity test (Study 2).

### 2.3. Aim 3: Predictive validity

Content validity is just one aspect of validity. In Studies 3–11, we provide additional validity evidence for LWA by showing how Conway et al.’s (2018) LWA scale is related to four different types of phenomena strongly linked to authoritarianism models. In so doing, we counter several claims posed by critics of LWA research. Many of those claims center around arguments that LWA is not uniquely predictive of important phenomena in the field. As we shall see, we provide empirical evidence that these attacks on LWA’s unique predictive validity are without merit.

#### 2.3.1. Threat

Almost all prominent theories of authoritarianism maintain that it is psychologically linked to perceptions of threat or danger (e.g., Altemeyer, 1998; Feldman, 2003; Jost et al., 2003; Peterson and Gerstein, 2005; Duckitt et al., 2010; see Choma and Hanoch, 2017, for discussion). Indeed, it is “widely accepted” that authoritarianism and threat are empirically linked (Duckitt, 2013, p. 1). Studies 3–6 test the degree that this theoretical notion applies to LWA as well.

#### 2.3.2. Restrictive norms

Restrictive norms are central to conceptualizations of authoritarianism (e.g., Altemeyer, 1996; Feldman, 2003; Duckitt et al., 2010). Thus, we would expect that LWA would uniquely predict support for left-leaning norms that focused on restriction. Study 7 tested one such norm: Support for restrictive language norms.

#### 2.3.3. Outgroup dislike

Stereotypes and prejudice are typically associated with conservatives in general, and right-wing authoritarianism in particular (e.g., Jost et al., 2003). More recent research has suggested, however, that prejudice can occur on both sides of the political spectrum. For example, work has revealed that liberals show negative attitudes towards African Americans when they believe they possess conservative attitudes such as religious fundamentalism (Chambers et al., 2013). Paralleling work on the unique contribution of right-wing authoritarianism to prejudice, newer work has tied *left-*wing authoritarianism to group attitudes by revealing that persons high in LWA are more likely to exhibit the equivalent of *modern racism* on a scale that targets Christian fundamentalists (Conway et al., 2018).

This prior work on LWA has been criticized for having “selected targets of prejudice that are rarely victims of prejudice in the US” (Saunders et al., 2020). To fill in this gap, Studies 8 and 9 apply the LWA framework to two groups that have historically been the target of prejudice: Religious African American persons and Jewish persons who support Israel.

This is important because, in the modern U.S., the large majority of African Americans are religious (for example, 77% of African Americans believe that “the Bible is the Word of God”; Diamant, 2018). Extrapolating from survey data, a cautious estimate of the number of African Americans who “believe in the Bible” is 30 million persons. Similarly, Jews in the U.S. have historically been the targets of prejudice, and the majority of modern Jewish Americans support the state of Israel, with estimates as high as 90% (Newport, 2019). Extrapolating from survey data, a cautious estimate of the number of Jewish Americans who support Israel is 4 million persons. Thus, if persons high in LWA show negative attitudes towards these groups beyond political ideology, this suggests the unique contribution of LWA to potential prejudice on large groups of persons that have historically been the targets of prejudice.

#### 2.3.4. Cognitive rigidity

Authoritarianism is conceptually related to cognitive rigidity (Jost et al., 2003). Indeed, in their study on LWA, Conway et al. (2018) demonstrated that persons high in LWA showed higher levels of dogmatism and attitude strength in liberal-focused domains. However, these results have been criticized as potentially not representing anything beyond political ideology (Honeycutt and Jussim, 2020). To deal with this criticism, in Study 10 we re-analyze the data provided by their LWA participants to control for political ideology. Further, in most of our studies, we control for ideology at the broadest level as self-reported ideology. While this method has many strengths because it does not smuggle the conclusion into the measurement, triangulation nonetheless suggests that we should also rule out the possibility that it is representative of specific liberal attitudes (and not liberal *authoritarian* attitudes). Study 10 allowed for a very rigorous test of that by including a measurement of attitudes *on the domain of interest* with respect to dogmatism (environmental issues). As we will see, these results overwhelmingly suggest that it is authoritarianism, and not liberal content, that accounts for the LWA-Dogmatism relationship. Study 11 provides an additional test of the predictive validity of LWA on Dogmatism and Need for Closure while using a more nuanced, content-focused measurement of ideology (social and economic conservatism).

### 2.4. Aim 4: Expand cross-cultural evidence

Much of the debate around LWA has centered only on authoritarianism in Western democracies. However, this Western, Educated, Industrial, Rich, and Democratic (WEIRD) group of participants is not representative of the whole earth’s population (Henrich et al., 2010), and much more work is needed on individual differences in non-WEIRD samples (Cooper, 2016). As a result, it is important to evaluate the LWA question in samples beyond the U.S. This is especially true as there are reasons to expect that left-wing authoritarianism might be more prominent in non-Western contexts (e.g., McFarland et al., 1992, 1993; Jost et al., 2003; Todosijević, 2005; Todosijević and Enyedi, 2008; De Regt et al., 2011).

Expanding the cultural reach of authoritarianism research also ameliorates the problems associated with authoritarianism measurement and provides a fairer scientific test of the LWA question. As noted earlier, it is difficult to produce authoritarianism measurements that do not contain ideological content. Even methods that *appear* ideologically neutral often are not neutral *in practice* because they contain implied ideological content *to participants*. For example, when participants are asked to report agreement with the statement “Our country needs a strong leader right now” (e.g., Sprong et al., 2019), it very likely matters to participants whether or not they imagine a person whose political views they agree with is the *strong leader* in question. If conservative persons imagine that the strong leader in question is liberal, it would very likely change their answers to the question (compared to believing that the strong leader was conservative).

Thus, such generic authoritarian language does not produce ideological content-free measurements. However, this problem can be offset somewhat in a large multi-national study. If this kind of “strong leader” item were collected in only one context, it is likely just as ideologically biased as any other kind of measure. However, when averaged across multiple contexts that vary in the ideological bent of the political leadership (and thus likely vary in the way the item maps on to participant beliefs about the ideological bent of the hypothetical person in the question), it allows for a more (though hardly perfect) ideological content-free test.

This means that, as a field, we need to include as many national contexts as possible while using the same set of items. Previous cross-cultural evidence related to authoritarianism, while important, has not comprehensively compared RWA and LWA on expansive cross-cultural footing. For example, Napier and Jost (2008) measured authoritarianism in 19 democratic (mostly Western) countries from Wave 4 of the World Values Survey. However, their study specifically only focused on a region of the world where one would expect LWA to be lowest (Western democracies) and thus does not advance our knowledge very far beyond WEIRD samples.^1^ In spite of this biased sample, they nonetheless found generally only small-to-moderate effect sizes for the conservatism-authoritarianism relationship. A later study provided more comprehensive evidence: Across 28 nations (Sprong et al., 2019) using a generic authoritarian leadership measurement (similar to that used in our Study 12 below), researchers found a small-to-moderate association between authoritarianism and conservative political orientation (*r* = 0.20).

However, both of these prior studies, while important, were limited in their sample of nations. In Study 12 (Aim 4), we nearly doubled the nation-level sample size and included a larger scope of cultural regions for which LWA might be more manifest.

## 3. Summary of the present studies

In total, in the present work we present a wide array of novel evidence concerning the real-life prevalence and importance of LWA within (Studies 1–11) and outside (Study 12) the USA. We divide this work into the four aims discussed above. In Aim 1 (Study 1), we ask participants in the US about their own perceptions of LWA persons in their lives (and discover that participants on both sides of the political spectrum, to a surprising amount, identify a descriptively important number of left-wing authoritarians in their own lives). In Aim 2 (Study 2), we ask participants to judge the degree that items from Conway et al.’s (2018) LWA questionnaire are measurements of authoritarianism (and discover that they are rated as good measurements of authoritarianism). In Aim 3 (Studies 3–11), we evaluate the degree that persons scoring high on this LWA scale show the properties that prominent authoritarianism theories suggest an authoritarian person should have. Studies 3–6 reveal that persons high in LWA show heightened sensitivity to threat. Study 7 shows high-LWA persons have more support for a restrictive social norm. Studies 8 and 9 reveal that high-LWA participants show more negative ratings of African-Americans and Jews, while Studies 10 and 11 reveal that high-LWA participants show higher scores on rigidity measurements such as dogmatism and need for closure. Across studies 3–11, these effects occur when controlling for political ideology and when looking only at liberals. In Aim 4 (Study 12), we use a common authoritarianism questionnaire from the World Values Survey to provide evidence of Left-Wing Authoritarianism around the globe. Taken together, these results show that there is something beyond mere ideology that causes LWA measurements to predict these important phenomena; and the best explanation for that *something beyond* is that it is *authoritarianism*.

## 4. Aim 1: Descriptive validity (study 1)

### 4.1. Study 1 methods

The focus of Study 1 was purely descriptive in that it evaluated the everyday occurrence of authoritarianism in lay populations. For Study 1, we gave participants categories of persons (e.g., family, co-worker) and asked them to identify authoritarians in their lives on both sides of the political spectrum.

***Participants*.** Four hundred forty-one U.S. adults were recruited using Amazon’s *Mechanical Turk (MTurk)*. *MTurk* has been validated for use as a representative sample for research related to politics and political ideology (see, e.g., Clifford et al., 2015, Kennedy et al., 2018) and generally shows similar results as other samples (e.g., Houck et al., 2014). Further, *MTurk* has been validated for use in work on authoritarianism (Choma and Hanoch, 2017; Ludeke et al., 2018). As a result, *MTurk* is an excellent choice for work on U.S. authoritarianism. The sample was 55% female, had an average age of 38, and was slightly left-leaning politically (4.3 on a political conservatism scale with 4.5 as the midpoint).

***Initial Directions to Participants*.** Participants were randomly assigned to one of two different sets of directions. In the first set of directions (*Definition Given*), we gave participants a standard definition of authoritarianism. This definition was drawn from prior work suggesting that authoritarianism has three primary aspects (e.g., Altemeyer, 1996; Feldman, 2001; Duckitt et al., 2010). See Supplementary material for the exact wording.

Participants in the other condition (*Definition Generated*) were given no definition, but instead were asked to generate their own definition. Participants in this condition generally seemed to understand the authoritarianism construct and, as can be seen in the Supplementary material, this manipulation did not impact the results. Thus, the manipulation will subsequently be dropped in the main text for ease of presentation.

***Authoritarianism Measurements: Mean Number.*** Participants then completed a series of parallel measurements asking them how many authoritarians they could identify in their lives across various categories for both liberals and conservatives. For example, participants were asked: *In your family (including all extended family), how many authoritarians can you think of that are politically liberal?* They were then given options 0, 1, 2, 3, 4, 5, 6–10, and more than 10.

A parallel question was asked for how many family members were authoritarians who were politically conservative: *In your family (including all extended family), how many authoritarians can you think of that are politically conservative?*

Using this method, we asked participants to identify left- and right-wing authoritarians across four different categories: Family, Friends or Acquaintances, Co-Workers, and News/TV/Movie/Sports Personalities. We further asked participants to simultaneously consider (and report the overall number of) *all* the authoritarian people they could identify who were politically liberal and politically conservative (scale options = 0, 1, 2, 3, 4, 5, 6–10, 11–15, and greater than 15). To convert these to a single number per variable, when participants chose ranges, we entered the midpoint of the range (e.g., 6–10 became 8). When the “greater than” option was chosen, we added two to the end of the range to estimate the number.

We used two primary summary scores with complementary strengths and weaknesses. First, we used participants’ own report of the total number of authoritarians they knew on the right and the left (referred to in the tables as *TOTAL: REPORTED*). We further took the sum of all the four categories (referred to as *TOTAL: SUM*).

***Authoritarianism Measurements: Most Authoritarian Person.*** Participants were then asked to consider the most authoritarian person they could identify in their life across each of the four categories. In each case, they were asked whether or not the representative *most authoritarian person* was *liberal*, *conservative*, or *neither/do not know*. Finally, participants were asked to think of the most authoritarian person overall in their lives and identify whether the person was liberal, conservative, or neither/do not know. We created an additional summary score conceptually identical to the summary for the mean number measurements by taking the average percentage across the four types of authoritarians.

***Participant Ideology*.** All participants further completed a standard two-item political conservatism scale, with items anchored by liberal/conservative and democratic/republican (e.g., Jost et al., 2003, 2008; Conway et al., 2012). In order to provide easy descriptive summaries, for Study 1 we converted this measurement to a dichotomous measure in a manner identical to prior research (Conway et al., 2016a,b, 2018) by considering people above the mid-point *conservative* and people below the midpoint *liberal* (people right at the mid-point were dropped for all analyses including this variable; *n* = 395 for those analyses, with 244 liberals and 151 conservatives).

### 4.2. Study 1 results and discussion

Although the primary purpose of Study 1 is to investigate the descriptive nature of participant perceptions of left-wing authoritarians, we present inferential comparisons for completeness. Descriptive results for all measures are presented in Tables 1, 2. Because (as the tables reveal) the results tell the same story across all categories, for the sake of brevity, in this narrative we focus only on the overall summary scores.

**Table 1:** Study 1: identification of left- and right-wing authoritarians in everyday life.

	Liberal authoritarians	Conservative authoritarians
**Mean number of authoritarians**		
*Family*	*1.7*	*2.8*
*Friends*	*2.4*	*2.8*
*Co-Workers*	*2.4*	*2.8*
*News/TV/Movie/Sports*	*5.1*	*6.0*
**TOTAL (SUM)**	**11.7**	**14.4**
**TOTAL (REPORTED)**	**7.8**	**10.3**
**Most authoritarian person**		
*Family*	*33%*	*57%*
*Friends*	*36%*	*48%*
*Co-Workers*	*27%*	*51%*
*News/TV/Movie/Sports*	*38%*	*50%*
**TOTAL (AVERAGE)**	**33%**	**52%**
**TOTAL (REPORTED)**	**34%**	**61%**

*N = 441*.

**Table 2:** Study 1: identification of left- and right-wing authoritarians in everyday life, broken down by political identification of the participant.

	Liberal Participants		Conservative Participants	
	Liberal	Cons.	Liberal	Cons.
**Mean number of authoritarians**				
*Family*	*1.6*	*2.6*	*1.8*	*3.1*
*Friends*	*2.5*	*2.6*	*2.4*	*3.2*
*Co-Workers*	*2.3*	*2.9*	*2.5*	*2.9*
*News/TV/Movie/Sports*	*4.0*	*6.6*	*6.7*	*4.9*
**TOTAL (SUM)**	**10.5**	**14.8**	**13.4**	**14.0**
**TOTAL (REPORTED)**	**6.6**	**9.0**	**11.3**	**8.5**
**Most authoritarian person**				
*Family*	*34%*	*57%*	*31%*	*60%*
*Friends*	*34%*	*52%*	*37%*	*53%*
*Co-Workers*	*23%*	*57%*	*34%*	*41%*
*News/TV/Movie/Sports*	*29%*	*60%*	*50%*	*37%*
**TOTAL (AVERAGE)**	**30%**	**56%**	**38%**	**48%**
**TOTAL (REPORTED)**	**24%**	**71%**	**49%**	**48%**

Total *N* = 395. Conservative participant *n* = 151; liberal participant *n* = 244.

Consistent with prior assertions that right-wing authoritarianism is more prevalent than left-wing authoritarianism, participants reported significantly more right-wing authoritarianism for all summary measures of both mean number and most authoritarian person measures, all within-subjects *F*’s > 19.0, *p*’s < 0.001. However, more important to our present purpose, participants consistently identified a large number of left-wing authoritarians as well. As seen in Table 1, participants self-reported identifying 7.8 liberal authoritarians on average, and the sum total of the identified liberal authoritarians across the four categories was 11.7 authoritarians.^2^

This remains the case even when focusing only our liberal participants.^3^ As seen in Table 2, liberal participants self-reported identifying 6.6 liberal authoritarians on average, and the sum total of the identified liberal authoritarians (identified by liberal participants) across the four categories was 10.5 authoritarians.^4^

Indeed, all the mean numbers for liberal authoritarians (both individual categories and summary scores) presented in Tables 1, 2 are significantly different from zero using one-sample *t*-tests (*p*’s < 0.001). More importantly, the descriptive statistics reveal that most people report identifying a substantial number of left-wing authoritarians in their lives. Further, even liberal participants report that 24% (or 30%, depending on the summary measure) of their most authoritarian persons are, in fact, liberal. As a thought experiment, extrapolated to the U.S. population as a whole, these data would mean that tens of millions of people – including liberals – would identify a left-wing authoritarian as the most authoritarian person in their life. This would translate to literally *millions* of (very real) left-wing authoritarians in the U.S. presently, across all walks of life. Thus, while the present data do suggest participants identify more right-wing than left-wing authoritarians, they also suggest that participants – even liberal participants – identify a meaningfully large number of left-wing participants in their lives.

## 5. Aim 2: Content validity (study 2)

### 5.1. Overview

Content validity is a complicated and multi-faceted concept. At a most basic level, however, content validity is a simple question: Does a questionnaire set measure what it purports to measure? One of the most basic, direct, and important ways to determine if a scale measures what it is supposed to measure is to provide content judgments concerning whether or not scale items are measuring the key construct. Indeed, this method has been used in other authoritarianism work (e.g., Funke, 2005; Dunwoody and Funke, 2016). In Study 2, we thus provided such direct validity evidence by asking participants if items from commonly-used LWA and RWA questionnaires do, in fact, measure authoritarianism. To the degree that participants believe they do, this provides a piece of evidence (in a larger puzzle) that LWA is a real construct that is meaningfully measured by a recently-developed LWA scale.

### 5.2. Study 2 methods

Because Conway et al.’s (2018) LWA scale was purposefully designed to be parallel to a version of Altemeyer’s (1996) RWA scale, we selected parallel items from each scale for this validity test.^5^ For a discriminant validity comparison group, we further selected items from a widely-cited Big 5 Personality inventory. In all cases, we asked participants to identify the degree that they believed that someone scoring high on an item would be an authoritarian person.

**Participants.** Four hundred seventeen U.S. adults (50% female, mean age = 38) were recruited using Amazon’s *Mechanical Turk (MTurk)*. The sample was slightly left-leaning politically (4.2 on a political conservatism scale with 4.5 as the midpoint). Participants were randomly assigned to receive one of the three sets of parallel items described in more detail below: RWA, LWA, or De-Politicized LWA.

**Instructions to Participants.** All participants were first given the definition of authoritarianism used in Study 1’s *Definition Given* condition, which contains a summary of the widely-accepted three-aspect model of authoritarianism. Then participants read a description of their task, which asked them to consider the likelihood that someone who answered

“yes” to each question would in fact be an authoritarian (please see Supplementary material for more details).

After that, participants were presented items (described below) for making judgments.

**Selection of LWA and RWA items.** From the LWA and RWA scales, we selected all the pro-trait items (see Supplementary material for all items and selection logic) for this validity test. This left 10 items each to be used in the validity test for both LWA and RWA.

For LWA, we further created a set of De-Politicized LWA items by removing all clearly political language (such as “liberal” and “conservative”) and, when necessary, replacing politicized words with generic alternatives (e.g., replacing “progressive ways and liberal values” with “our group’s values”). The goal of these items is to see what, if any, biases people might have in making attributions about authoritarianism to left- versus right-wing persons. To the degree that the de-politicized items are rated by participants as more *authoritarian*, it suggests the items are measuring authoritarianism – but people are biased to believe otherwise (as some research suggests they will be; Frimer et al., 2014).

***Selection of Big 5 Inventory Items.*** For discriminant validity, we further selected the 9 pro-trait items from the highly-cited MINI Big 5 Inventory (Donnellan et al., 2006; see Supplementary material).

***Participant Ideology*.** All participants further completed the same standard two-item political conservatism scale used in Study 1. As in Study 1, we converted this measurement to a dichotomous measure in a manner identical to prior research (*n* = 365 for those analyses, with 236 liberals and 129 conservatives).

### 5.3. Study 2 results and discussion

**Figure 1:**
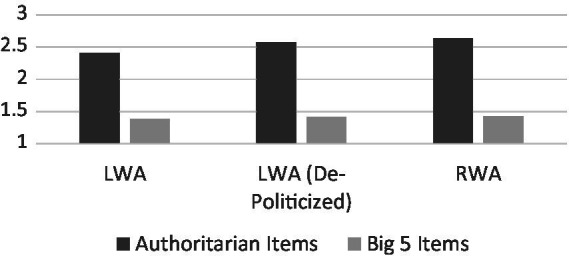
Discriminant validity of authoritarianism scales by type of scale.

As seen in Figure 1, results revealed clear evidence of discriminant validity for both LWA (standard and de-politicized) and RWA as an authoritarianism measurement. Paired-sampled *t*-tests comparing each authoritarianism questionnaire set’s average to the average from the comparison group revealed strong and significant validity effects for LWA (*t*[135] = 19.13, *p* < 0.001, *d* = 0.36, LCI = 0.02, UCI = 0.70), De-Politicized LWA (*t*[140] = 20.28, *p* < 0.001, *d* = 0.36, LCI = 0.02, UCI = 0.69), and RWA (*t*[135] = 22.12, *p* < 0.001, *d* = 0.37, LCI = 0.03, UCI = 0.71). Looked at another way, one-sample t-tests revealed that all six tests significantly differed from the mid-point of the scale (2), with the authoritarianism questionnaires skewing greater than the midpoint (*t*’s > 12.1, *p*’s < 0.001) and the Big 5 questions skewing below the midpoint (*t*’s < −0.14.6, *p*’s < 0.001). This suggests that the authoritarianism questions for all three scales are indeed measuring authoritarianism – as they lean heavily towards the “very likely authoritarian” end of the scale – while the Big 5 questions do not measure this construct.

These metrics overwhelmingly provide content validity support for the pro-trait items in Conway et al.’s (2018) LWA measurement. As can be seen from Figure 1, however, LWA did show slightly lower (though still high in absolute terms) discriminant validity than RWA. We explore this in more detail in the Supplementary material. Regardless of these small differences across scale types, the present results clearly provide direct evidence of the content validity of the LWA scale as a measurement of authoritarianism. It showed strong discriminant validity. Not only did participants rate it as substantially higher than a scale not designed to measure authoritarianism, but they also rated it as significantly leaning towards the end of the scale, clearly indicating it is measuring authoritarianism in absolute terms.

## 6. Aim 3: Predictive validity (studies 3–11)

### 6.1. Methods

Studies 3–11 involved substantive predictive tests of Conway et al.’s (2018) LWA measure. For the sake of brevity, we consider this set of studies together. Unless otherwise noted, all participants in Studies 3–11 were randomly assigned to complete either Conway et al.’s (2018) LWA measure or the parallel Altemeyer (1996) RWA measure. Further, all participants completed either the standard 2-item political identification measure used in Studies 1 and 2 (Studies 3–10) or the Social and Economic Conservatism measure (Study 11).

In each study, participants additionally completed measurements that authoritarianism theory suggests a true authoritarianism measure would predict. We report more detail on each study’s methods and results in the Supplementary material. Here, we focus only on the key variables that differed across each study. Sample details for each Study can be found in Table 3.

**Table 3:** Studies 3–11 sample characteristics.

Nation	Characteristic			
	*N*	Source	Dependent measures	Covariates
**Threat**				
Study 3*	4,855	*MTurk*	Perceived Geographical Ecological Stress	Ideology, Income
Study 4	1,084	*MTurk*	Perceived COVID Stress, Desired Restriction and Punishment	Ideology, Income, Age, Sex, Education
Study 5*	421	*MTurk*	Belief in a Dangerous World	Ideology
Study 6	533	*MTurk*	Political Outgroup Threat	Ideology, Age, Sex
**Restrictive Norms**				
Study 7	350	*MTurk*	Restrictive Norms Support	Ideology, Age, Sex
**Outgroup Dislike**				
Study 8	271	*MTurk*	Dislike of African-Americans and Jews	Ideology, Age, Sex, Population
Study 9	169	*MTurk*	Dislike of African-Americans and Jews	Ideology, Age, Sex, Population
Rigidity				
Study 10a	178	College Student	Dogmatism, Modern Racism, Attitude Strength	Ideology, Attitude Content
Study 10b	147	*MTurk*	Dogmatism, Modern Racism, Attitude Strength	Ideology, Attitude Content
Study 11	479	*MTurk*	Dogmatism, Need for Closure	Social and Economic Conservatism

* Pre-registered study on OSF.

#### 6.1.1. Study 3

Study 3 measured participants’ perceived level of ecological stress in their local geographical environment. Importantly for the present analysis, participants resided in all regions of the United States, including participants from all 50 states. No one region dominated, and percentages from each state reflected the population distribution from the nation as a whole: The largest percentages of participants (by state) resided in California (11%) Florida (9%), Texas (8%), New York (6%), Pennsylvania (5%), Ohio (4%), North Carolina (4%), Michigan (4%), Illinois (4%), and Georgia (4%).

Participants were asked a series of questions related to the likelihood of prevalence of various ecological threats in the area in which they live. These threats were drawn from prior work on the effect of ecological stress on the emergence of cultural beliefs related to authoritarianism and freedom (e.g., Kitayama et al., 2006, 2010; Murray and Schaller, 2010; Fincher and Thornhill, 2012; Van de Vliert, 2013; Conway et al., 2014, 2017a; Beall et al., 2016; Oishi et al., 2017; Van de Vliert and Conway, 2019). These included a question each for *pathogen prevalence, natural disaster prevalence, harsh climate prevalence, mountain* (i.e., *frontier topography*) *prevalence*, and *general ecological stress.* For example, participants were asked “*I feel the primary area where I live has a lot of disease*.” These five items were all modestly correlated with each other and thus we further produced a summary *Ecological Stress* score (conceptually similar to Conway et al., 2017a) by converting each item to a *z*-score and averaging (standardized *alpha* = 0.72).^6^

#### 6.1.2. Study 4

Participants in Study 4 completed six items concerning how threatened or worried they were about COVID-19, for example: “Thinking about the coronavirus (COVID-19) makes me feel threatened” (standardized *alpha* = 0.88).

Participants in Study 4 also completed multiple items concerning their political beliefs about their government’s response to the crisis. We focus here on two cross-governmental dimensions most relevant to participants’ feelings of threat related to COVID-19 (all scale *alphas* > 0.86): The degree they wanted the government to restrict citizens to help stop the spread of the virus (*Restriction*; for example, “I support [Federal/State/City] government measures to restrict the movement of American citizens to curb the spread of Coronavirus (COVID-19)”), and the degree that participants wanted their governments to punish citizens who violated social distancing rules (*Punishment*; for example, “I want my [Federal/State/City] government to severely punish those who violate orders to stay home”). For each belief dimension, participants completed six questions (two for each level of government), and we aggregated the six items for each dimension to create scores for *Restriction* and *Punishment*.

#### 6.1.3. Study 5

Participants in Study 5 were randomly assigned to a version of the Belief in a Dangerous World scale. Half of the participants received the original conservative scale. In this scale, some of the items emphasize ideological content more harmonious with a conservative ideological focus, such as the destruction of the world by God or the preponderance of crime.

Half of the participants completed a modified version of the *BDW* scale designed to focus on threats in domains more harmonious with the ideological focus of liberals: Environmental concerns, lack of medical care, and fighting wars. This modified *Belief in a Dangerous World* scale inserted a new content domain for seven of the 12 items, such that it intentionally pointed the potential danger in the item to liberal content domains, while keeping the danger-related language the same. See Supplementary material for more details.

Inter-item reliability for the scale was satisfactory for both versions (Belief in a Dangerous World Conservative *alpha* = 0.87; Belief in a Dangerous World Liberal *alpha* = 0.81).

#### 6.1.4. Study 6

Participants completed two items of threat-based concerns about the U.S. presidential administration (*alpha* = 0.94): “When I think of Donald Trump, it makes me feel a sense of threat,” and “When I think of Donald Trump, it makes me feel anxious for my country’s future.”

Participants also completed single-item measures of their intent to vote for the Democratic nominee (at that time, yet to be determined) in the upcoming 2020 election, and their intent to vote for Donald Trump in the upcoming 2020 election (we reverse-scored this item as Opposition to Trump).

#### 6.1.5. Study 7

Participants in Study 7 completed four items concerning their support for restrictive communication norms (items and introduction were adapted directly from Conway et al.’s, 2017b restrictive PC norms condition).

#### 6.1.6. Study 8

All participants completed standard “feeling thermometer” measurements drawn from prior research (e.g., Schaller et al., 2002; Dyrbye et al., 2019) concerning their own personal views of groups. The groups rated by each participant included *Bible-believing African-American men, Bible-believing African-American women, Strong supporters of the nation of Israel’s interests who are also Jewish men, and Strong supporters of the nation of Israel’s interests who are also Jewish women*. Because in each case the results were nearly identical for men and women, we collapsed these measurements into a single feeling thermometer for each group for ease of presentation.

To distinguish their private views from their views of society (see Schaller et al., 2002; Nosek, 2005), all participants also completed responses to the same six target groups while considering, not their own views, but the views of society as a whole.

#### 6.1.7. Study 9

Study 9 was identical to Study 8 except that it also included a manipulation of whether the trait (Bible-believing, Support of Israel) or the group (African-American, Jewish) came first linguistically. As seen in the Supplementary material and in Table 4, this manipulation did not affect the results, thus effectively ruling out a language presentation bias issue from Study 8.

**Table 4:** Studies 3–11: LWA effects for both whole-sample and within-group (Liberals-only).

	Whole-sample LWA	Liberals-only LWA
Perceived ecological threat (Study 3)	0.15***	0.10***
COVID threat sensitivity (Studies 4)	0.15**	0.09
Desired restriction (Study 4)	0.16**	0.13*
Desired punishment (Studies 4)	0.23***	0.09^
Belief in a dangerous world (Study 5)	0.42***	0.64***
Trump threat sensitivity (Study 6)	0.18***	0.25**
Restrictive communication norms (Study 7)	0.19***	0.08
Negative views/African Americans (Study 8)^a^	0.50***	0.54***
Negative Views/African Americans (Study 9)^a^	0.58***	0.67***
Negative views/Jews (Study 8)^a^	0.42***	0.37***
Negative views/Jews (Study 9)^a^	0.39**	0.47**
Dogmatism (Study 10a)	0.29***	0.39***
Dogmatism (Study 10b)	0.42***	0.40***
Modern racism/Relig. minorities (Study 10a)	0.53***	0.60***
Modern racism/Relig. minorities (Study 10b)	0.62***	0.64***
Attitude strength (Study 10a)	0.09	0.39***
Attitude strength (Study 10b)	0.06	0.41***
Dogmatism (Study 11)	0.21***	0.29***
Need for closure (Study 11)	0.23***	0.11^
**AVERAGE EFFECT SIZE**	0.30***	0.35***

All metrics = standardized betas. Across-Group LWA correlations always control for political ideology (every study) + any available demographic factors. ^a^Reverse-scored and re-named so that positive correlations always equal the conceptually-expected direction for LWA.

*** *p* < = 0.001;

** *p* < = 0.01;

* *p* < = 0.05;

^ *p* < = 0.15.

#### 6.1.8. Study 10a and 10b

In their study on LWA, Conway et al. (2018) demonstrated that persons high in LWA showed higher levels of dogmatism, modern racism, and attitude strength in liberal-focused domains. However, these results have been criticized as potentially not representing anything beyond political ideology (Honeycutt and Jussim, 2020). To deal with this criticism, we here re-analyze the data provided by their LWA participants to control for political ideology. Further, up to this point, we have only controlled for ideology at the broadest level as self-reported ideology. While this method has many strengths because it does not smuggle the conclusion into the measurement, triangulation nonetheless suggests that we should also rule out the possibility that it is representative of specific liberal attitudes (and not liberal *authoritarian* attitudes). Study 10 allowed for a very rigorous test of that by including a measurement of attitudes *on the domain of interest* with respect to dogmatism. As we will see, these results overwhelmingly suggest that it is authoritarianism, and not liberal content, that accounts for the LWA-Dogmatism relationship.

All participants completed measurements of domain-specific dogmatism based on Rokeach’s Dogmatism scale (adapted from Rokeach, 1960), an adapted Modern Racism scale that targeted religious minorities instead of ethnic minorities (adapted from McConahay, 1986), and a measurement of the strength of their attitudes about climate change (adapted from Conway et al., 2008, 2011).

Further, imbedded in the questionnaire was an item pertaining to the specific attitude domain that the dogmatism questionnaire is about (climate change): “How much do you agree with this statement?: Global warming is occurring and is human caused.” Answers were given on a 1–9 scale where 1 = completely disagree and 9 = completely agree.

#### 6.1.9. Study 11

In Study 11, we use a scientifically-validated measure that captures more specific ideological attitudes on two different dimensions: The Social and Economic Conservatism Scale (Everett, 2013). We focus on conceptually replicating the LWA-cognitive rigidity effects from Study 10 while controlling for this measure.

Participants completed two measurements directly relevant to cognitive rigidity: Altemeyer’s (1996) Dogmatism measure and the short version of the Need for Closure Scale – Revised scale (Roets and Van Hiel, 2011). Finally, as a political ideology covariate, participants completed the 12-item Social and Economic Conservativism Scale (Everett, 2013), a scale which measures people’s favorability towards conservative social and economic policies, respectively.^7^

### 6.2. Aim 3 results

#### 6.2.1. Whole sample and within-group analyses

In Table 4, we present two complementary sets of analyses for separating ideology from authoritarianism in each study: (1) Across-group analyses that control for political ideology and (2) within-ideological group analyses (see Wronski et al., 2018, for an example). These methods for separating out ideology from authoritarianism have complementary strengths and weaknesses.

For across-group analyses (left-hand side of Table 4), in all cases we performed hierarchical regressions where we evaluated the strength of LWA’s relation to the target DVs at Block 2 when entering political ideology (and any available demographic covariates; see Table 1). For within-group analyses (right-hand side of Table 4) we analyzed all key LWA effects by looking only at persons who scored on the liberal side of the 1–9 ideology scale (because the scale is anchored by 1 = liberal and 9 = conservative, for these analyses, we only included persons scoring below the midpoint). For Study 11, we included participants who scored below the midpoint on the scale for both the social and economic conservatism subscales. For ease of understanding, when necessary we reverse-scored (and reverse-named; see Table note) variables so that positive numbers always meant the expected LWA effect. We also combined variables in some cases to make the results easier to digest (using the disaggregated variables yielded an identical set of results; see Supplementary material).

As can be seen in Table 4, in the vast majority of cases, both whole-sample analyses controlling for ideology and within-group analyses evaluating only liberals revealed a largely consistent pattern. Specifically, LWA was significantly positively predictive of all threat measurements: Ecological threat, COVID threat measurements, belief in a dangerous world, and outgroup political threat. LWA was further significantly predictive of punitive communication norms, dislike of African Americans and Jews, modern racism, and multiple measures of cognitive rigidity.

As would be expected in any such large analyses, some significant whole-sample effects became non-significant within-group, and some non-significant whole-sample effects became significant within-group. However, almost all analyses of each type showed effects in the same direction, and the vast majority were significant in both kinds of analyses. This can be easily seen by the evaluating the average effect sizes across studies, which are very similar for both whole-sample (average *beta* = 0.30) and within-group (average *beta* = 0.35) analyses.

These analyses suggest that when we control for ideology measures, LWA is still predictive of a whole range of variables related to authoritarianism. Further, when we compare liberals to other liberals with different degrees of authoritarianism, we still (in the main) get the conceptually-expected relationships in this large array of studies. This provides triangulating evidence that the left-wing authoritarian is more of a reality than a myth. Importantly, this reveals that this is not just about liberal ideology. There must be *something beyond* mere ideology that accounts for the additive predictive ability of LWA; and we believe that *something beyond* is best described as authoritarianism.

#### 6.2.2. Additional results specific to each study

##### 6.2.2.1. Study 6 mediation analyses

**Figure 2:**
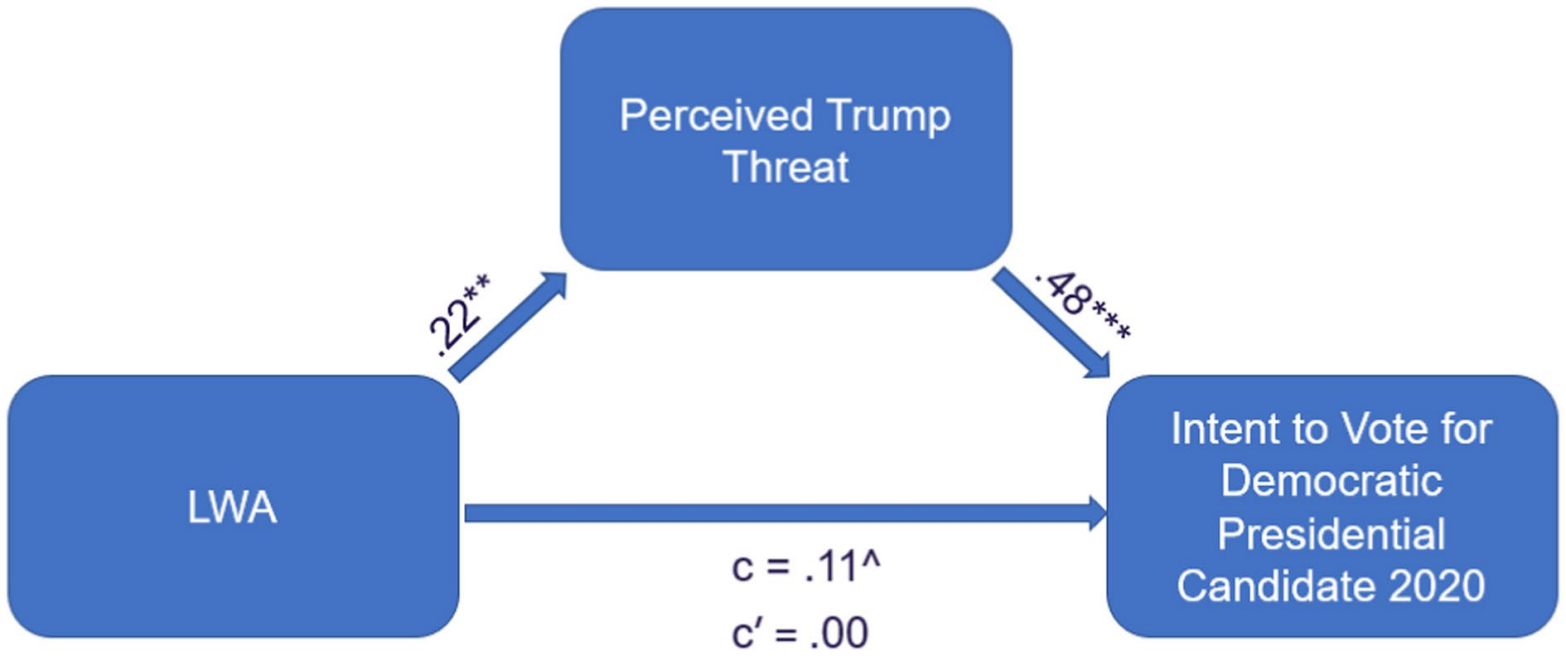
Study 6: LWA ➔ perceived trump threat ➔ voting intent.

Prior research suggests that, above and beyond political ideology, LWA uniquely predicts support of liberal candidates in two elections that were viewed as especially threatening, but did not do so in an election that was less threatening (Conway and McFarland, 2019; Conway et al., 2020b). However, while the authors of that work speculated that, consistent with models of authoritarianism, perceived threat from the candidate in power (e.g., Donald Trump) is the likely mechanism by which LWA operates, no research to date has directly reported measurements of threat from the government in power (see Conway and McFarland, 2019; Conway et al., 2020a). Study 6 also allowed a test of the mediational hypothesis (Figure 2). Specifically, we evaluated the LWA ➔ Threat ➔ voting intentions path (note that the predictive voting power of authoritarian measurements was recently used as validity evidence for conservative authoritarianism; Nilsson and Jost, 2020).

**Figure 3:**
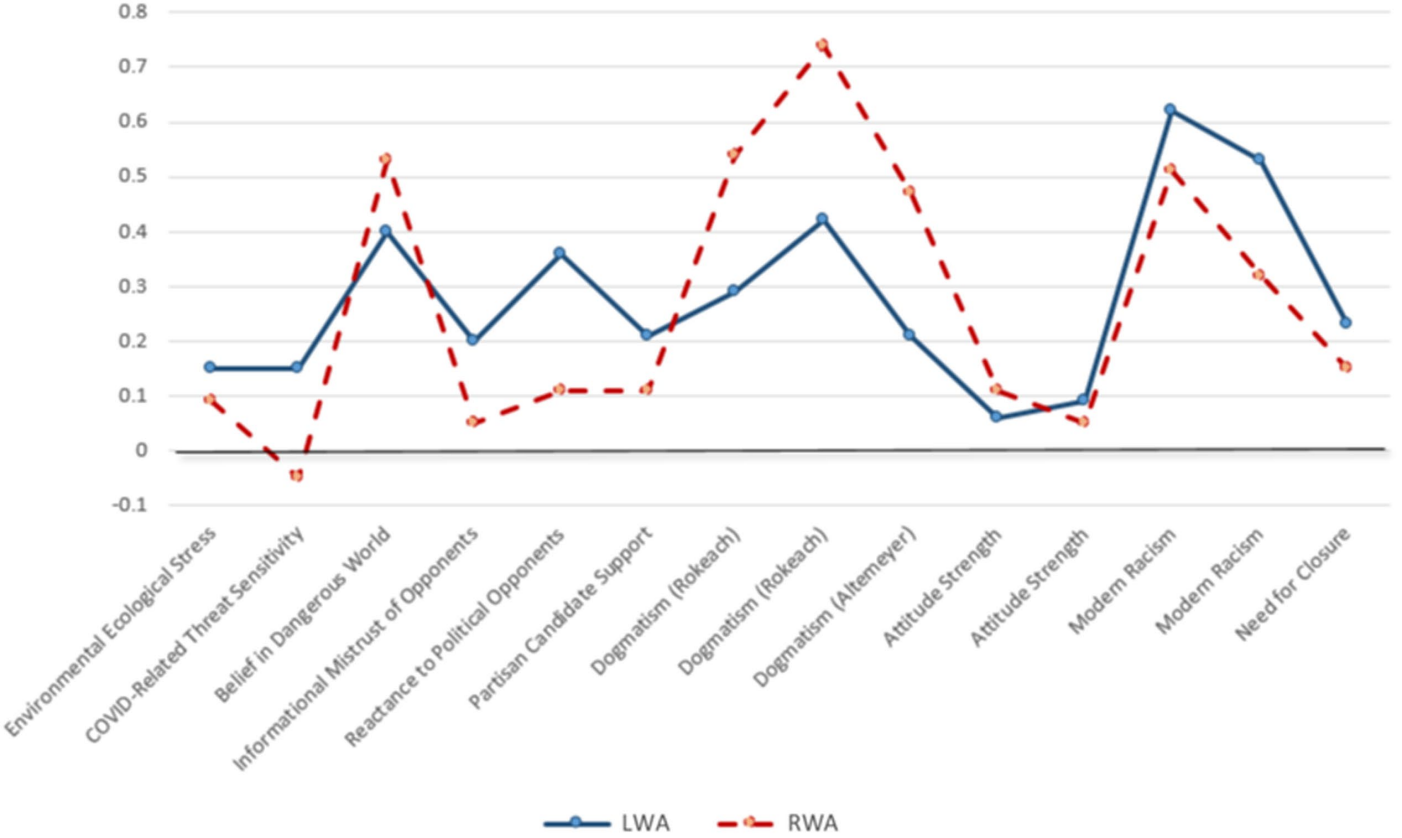
LWA and RWA effect sizes on comparable dependent measures.

Results are presented in Figure 3. As seen there, evidence revealed strong support (while controlling for political ideology) for an LWA ➔ Perceived Trump Threat ➔ Democratic Candidate Support Path, indirect effect = 0.07 (LCI = 0.02, UCI = 0.13), *p* = 0.004. Similarly, evidence revealed strong support for an LWA ➔ Perceived Trump Threat ➔ Oppose Trump Path, indirect effect = 0.08 (LCI = 0.04, UCI = 0.14), *p* = 0.003.^8^

This further demonstrates the practical utility of considering LWA as a construct in helping us better understand voting intent for the 2020 election. Specifically, high LWA persons’ heightened sensitivity to threats from Trump accounts for part of why they were especially likely to vote for the democratic party (again, controlling for ideology and demographic variables).

##### 6.2.2.2. Additional control in study 10

Study 10 participants completed an item pertaining to the specific attitude domain that the dogmatism questionnaire is about (climate change). Adding attitudes about climate change as a predictor did not alter the pattern of results for LWA predicting Dogmatism (Study 10a *beta* = 0.22, *p* = 0.014; Study 10b *beta* = 0.49, *p* < 0.001) or Modern Racism (Study 10a *beta* = 0.59, *p* < 0.001; Study 10b *beta* = 0.61, *p* < 0.001). LWA predicting Attitude Strength remained non-significant but became less positive (Study 10a *beta* = −0.03; Study 10b *beta* = −0.05).

It is noteworthy that controlling for climate change attitudes did not substantially alter the relationship between LWA and a dogmatism scale that specifically targeted environmental issues such as climate change. This suggests that the LWA-dogmatism relationship is not merely the result of attitudinal content overlap between the two scales – rather, it provides evidence that the scales are related because persons who score high on the left-wing authoritarianism scale are authoritarians who are especially prone to dogmatism.

##### 6.2.2.3. Comparable parallel RWA and LWA tests

Both in prior work (Conway and McFarland, 2019; Conway et al., 2021a) and in many of the studies reported here, participants were randomly assigned to complete either the LWA scale (Conway et al., 2018) or the parallel RWA scale (Altemeyer, 1996). Here, we offer tests of the relative strength of comparable RWA and LWA tests.

Our criteria for determining which effects are useful for comparison are outlined in the Supplementary material and focus on the potential structural and psychological equivalence of the measures. Table 5 reports the specific samples and metrics used in the present summary.

**Table 5:** Aim 3: metrics used for LWA and RWA summary comparisons.

Conceptual DV	Study *n*	Source	LWA Measure	RWA Measure	LWA DV	RWA DV
Environmental Ecological Stress	4,988	Present Paper, Study 3	Conway et al. (2018) LWA	Altemeyer (1996) RWA	Summary Scale Reporting Ecological Stress in Local Environment	Summary Scale Reporting Ecological Stress in Local Environment
COVID-Related Threat Sensitivity	1,084	Present Paper, Study 4	Conway et al. (2018) LWA	Altemeyer (1996) RWA	Summary Scale Reporting Perceived COVID Threat	Summary Scale Reporting Perceived COVID Threat
Belief in a Dangerous World	421	Present Paper, Study 5	Conway et al. (2018) LWA	Altemeyer (1996) RWA	BDW-Modified (adapted from Altemeyer)	BDW (Altemeyer)
Informational Mistrust of Opponents	340	Conway et al. (2014)	Conway et al. (2018) LWA	Altemeyer (1996) RWA	Item concerning distrust of Republican Party	Item concerning distrust of Democratic Party
Reactance to Political Opponents	340	Conway et al. (2021a)	Conway et al. (2018) LWA	Altemeyer (1996) RWA	Item concerning emotional reactance to Republican Party	Item concerning emotional reactance to Democratic Party
Partisan Candidate Support	1,582	Conway and McFarland (2019)	Conway et al. (2018) LWA	Altemeyer (1996) RWA	Voting intention for Obama when Republicans held power	Voting intention for Trump when Democrats held power
Dogmatism	178	Present Paper, Study 10a	Conway et al. (2018) LWA	Altemeyer (1996) RWA	Rokeach Dogmatism Scale (Environmental Issues)	Rokeach Dogmatism Scale (Religious Issues)
Dogmatism	147	Present Paper, Study 10b	Conway et al. (2018) LWA	Altemeyer (1996) RWA	Rokeach Dogmatism Scale (modified to focus on Environmental Issues)	Rokeach Dogmatism Scale (modified to focus on religious issues)
Dogmatism	479	Present Paper, Study 11	Conway et al. (2018) LWA	Altemeyer (1996) RWA	Altemeyer Dogmatism Scale	Altemeyer Dogmatism Scale
Attitude Strength	178	Present Paper, Study 10a	Conway et al. (2018) LWA	Altemeyer (1996) RWA	Composite Measurement of Attitude Strength (environmental issue)	Composite Measurement of Attitude Strength (religious issue)
Attitude Strength	147	Present Paper, Study 10b	Conway et al. (2018) LWA	Altemeyer (1996) RWA	Composite Measurement of Attitude Strength (environmental issue)	Composite Measurement of Attitude Strength (religious issue)
Modern Racism	178	Present Paper, Study 10a	Conway et al. (2018) LWA	Altemeyer (1996) RWA	Modern Racism Scale (Religious Minorities)	Modern Racism Scale (Ethnic Minorities)
Modern Racism	147	Present Paper, Study 10b	Conway et al. (2018) LWA	Altemeyer (1996) RWA	Modern Racism Scale (Religious Minorities)	Modern Racism Scale (Ethnic Minorities)
Need for Closure	479	Present Paper, Study 11	Conway et al. (2018) LWA	Altemeyer (1996) RWA	Short Version Need for Closure Scale	Short Version Need for Closure Scale

There are potential non-equivalence issues with any measure, and our set of chosen measures is not perfect; however, we feel the resulting set provides a reasonable test of comparable measurements across LWA and RWA. The high correlation of LWA and RWA effects across measurements (described below) supports this contention.

Results are presented in Figure 3. Three things are noteworthy about this analysis evaluating comparable effects across RWA and LWA. First, almost all of the expected correlations are above zero (and the only one that is not above zero is for RWA). Second, the overall effect size for RWA (average *r* = 0.27) and LWA (average *r* = 0.28) are virtually identical. Third, and perhaps most tellingly, RWA and LWA effect sizes tend to be similar across comparable measurements. In fact, the correlation between their effect sizes reported in Figure 3 is *r* = 0.65. This suggests that when you use comparable measures of authoritarianism and comparable dependent measures, you get similar results for RWA and LWA.

## 7. Aim 4: Expanded cross-cultural evidence (study 12)

As discussed earlier, Sprong et al. (2019) evaluated authoritarianism across 28 nations. In Study 12, we use a generic measurement of governmental authoritarianism similar to that in Sprong et al. (2019) to estimate the worldwide effect of ideology on authoritarianism. This authoritarianism measure was completed in Wave 6 of the World Values Survey (WVS; Inglehart et al., 2014). Specifically, over 66,000 participants across 54 nations completed a standard Political identification (left–right) item (e.g., Jost et al., 2003; Sprong et al., 2019; Nilsson and Jost, 2020) and a standard Authoritarian Governance endorsement questionnaire (e.g., Ariely and Davidov, 2010; Miller, 2017; Malka et al., 2020). The political identification item allows participants to self-identify on the left or right, offering no direct method overlap with authoritarianism. Further, the authoritarianism scale does not directly offer clearly left–right political positions, but rather asks participants about the degree that they would support various authorities countermanding normal governmental processes. Thus, measured in contexts with varying levels of governmental ideologies, these measurements help define the relationship between authoritarianism and ideology across 54 nations on 5 continents in a way that minimizes ideological cross-contamination.

For reasons outlined by other researchers (Jost et al., 2003; Malka et al., 2020; Conway et al., 2021b) and consistent with prior data (Sprong et al., 2019), we expected that in general, there would be a positive association between conservative ideological identification and authoritarianism across the world. However, we also expected that this effect would be moderated by the national political context, such that some nations would show less evidence of purely conservative authoritarianism. For example, we expected that this relationship between conservatism and endorsement of government authoritarianism would be less positive in contexts that had a history of influence by left-wing authoritarian governments (e.g., McFarland et al., 1992, 1993; Jost et al., 2003; Todosijević, 2005; Todosijević and Enyedi, 2008; De Regt et al., 2011). To our knowledge, Study 12 is by far the largest study on world-wide authoritarianism to date.

### 7.1. Study 12 method

***Participants*.** For Wave 6 of the WVS, 66,974 participants across 54 nations completed Ideology and Authoritarianism questionnaires.

***Ideological Conservatism*.** Participants were asked to position themselves on a 1–10 political left–right continuum, where 1 = “Left” and 10 = “Right.”

***Authoritarianism*.** Participants completed a three-item measure of endorsement of Authoritarian Governance that has been used in prior research to measure authoritarianism (e.g., Ariely and Davidov, 2010; Miller, 2017; Malka et al., 2020). These items ask participants the degree that they value “Having a strong leader who does not have to bother with parliament and elections,” “Having experts, not government, make decisions according to what they think is best for the country,” and “Having the army rule.” The items were on a scale from 1–4 where 1 = more agreement; as a result, we reversed-scored them and averaged them into a single *Authoritarianism* measure in a manner identical to prior research (e.g., Ariely and Davidov, 2010; Miller, 2017; Malka et al., 2020).^9^

***Western Democracies*** vs. ***Eastern Europe*.** To evaluate cross-cultural differences, we further compared the ideology-authoritarianism relationship across available Western democracies (defined in the typical manner as the EU15 plus Australia, Canada, New Zealand, Norway, Switzerland, and the United States; see Malka et al., 2020) and a region long influenced by more authoritarian left-wing ideology: Eastern Europe (see INSOL, 2020) and/or the former Soviet Republics (see Supplementary material for more details).

### 7.2. Study 12 results and discussion

**Figure 4:**
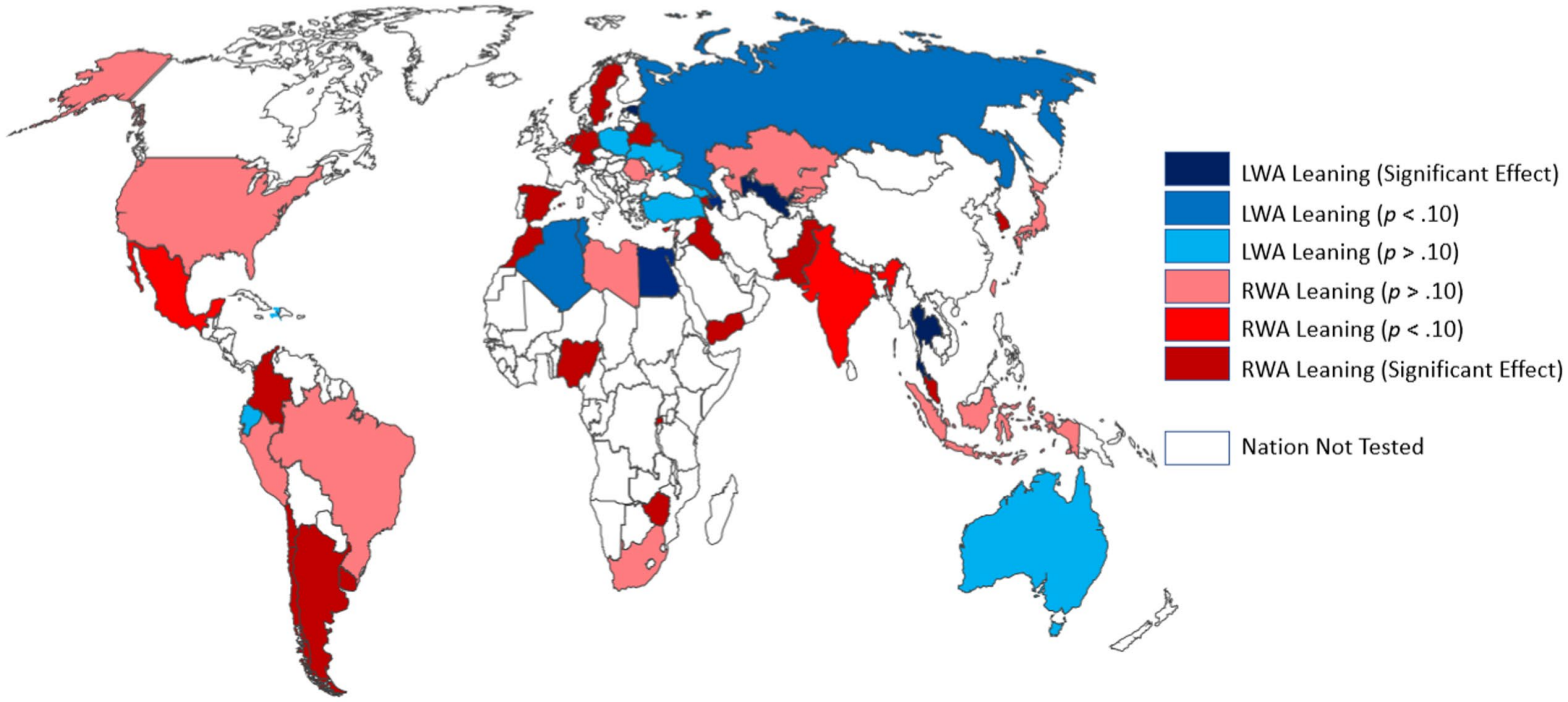
Left-wing authoritarianism (blue) versus right-wing authoritarianism (red) leanings around the world.

The results across the world are graphically depicted in Figure 4. As can be seen there, much variability across the world exists in the degree that authoritarianism leans left (blue) versus right (red).

We used several different statistical approaches to better understand this graphical representation.

**Multilevel Analyses.** We followed standard practices for Multilevel Analyses (e.g., Lorah, 2018; Sprong et al., 2019). Specifically, we used *R* (R Core Team, 2014) to estimate multilevel models with the *lme4* package (Bates et al., 2015). Our primary model (Model 2) predicted authoritarianism from ideology nested within countries.

In Model 1, we first estimated the effect of our level 2 predictor (group: country) on authoritarianism. This predictably showed that nations differed from each other in their levels of authoritarianism, *ICC* = 0.18, *p* < 0.001. In Model 2, we then added our level 1 predictor (conservative ideology) to the level 2 predictor (country). Consistent with prior researchers’ assertions about the general right-leaning nature of authoritarianism (e.g., Napier and Jost, 2008; Sprong et al., 2019; Conway et al., 2020b), this multilevel analysis revealed a positive relationship between conservative ideology and authoritarianism worldwide, *beta* = 0.01, *p* < 0.001. However, this relationship is very small and, as we will see below, within-country analyses clearly showed much variability across the world in the ideology-authoritarianism relationship.

**Within-country analyses.** A second (and related) method of evaluating the worldwide status of the ideology-authoritarianism relationship involves performing within-country analyses. For these analyses, both ideology and authoritarianism scales were first standardized within-country. As a result, any reported summary relationships represent the average within-country effect and thus directly control for across-country mean differences.

More specific results of within-country analyses are presented in Table 6. In that table, positive *betas* between authoritarianism and conservative ideology indicate a right-wing authoritarian leaning, while negative *betas* between authoritarianism and ideology indicate a left-wing authoritarian leaning (a *beta* of zero means that ideology and authoritarianism are unrelated, and thus the nation does not show a propensity towards either right-wing or left-wing authoritarianism on average).

**Table 6:** Study 12: individual-level conservative/liberal ideology predicting individual-level authoritarianism within nations.

Nation	*n*	ID-Auth *Beta*	Ideology mean	Ideology SD	Auth. Mean	Auth. SD
Yemen	1,250	0.29***	5.7	2.5	2.3	0.7
Netherlands	1,610	0.21***	5.5	2.0	2.0	0.5
Argentina	790	0.20***	5.5	1.8	2.1	0.7
Chile	701	0.19***	5.1	2.0	2.1	0.7
Spain	991	0.18***	4.8	1.9	2.1	0.6
Uruguay	833	0.18***	4.7	2.5	2.1	0.6
Morocco	197	0.16*	5.5	2.3	2.2	0.9
Armenia	1,002	0.12**	5.7	2.5	2.4	0.7
Columbia	1,242	0.11***	6.2	2.4	2.5	0.6
Iraq	976	0.10***	6.2	2.4	2.4	0.6
Cyprus	824	0.09**	5.2	2.7	1.9	0.7
Nigeria	1,759	0.09*	5.7	2.5	2.4	0.7
Sweden	1,118	0.08**	5.4	2.5	1.9	0.7
South Korea	1,187	0.08**	5.4	2.1	2.2	0.5
Malaysia	1,300	0.08**	6.6	1.9	2.5	0.7
Germany	1,829	0.07**	5.0	1.8	1.9	0.6
Belarus	1,476	0.07**	5.4	1.6	2.2	0.5
Pakistan	1,172	0.07*	7.4	2.0	2.7	0.6
Rwanda	1,527	0.06*	5.4	1.9	2.3	0.6
Zimbabwe	1,499	0.05*	5.3	2.7	2.0	0.6
Slovenia	681	0.07^	5.1	2.2	2.1	0.5
Mexico	1,903	0.04^	6.2	2.7	2.6	0.5
Kazakhstan	1,500	0.04	6.2	2.2	2.3	0.6
Kyrgyzstan	1,461	0.03	6.5	2.3	2.7	0.6
Lebanon	820	0.03	6.4	2.2	2.7	0.7
Libya	1,393	0.03	5.9	2.7	2.5	0.7
South Africa	3,003	0.02	6.3	2.1	2.6	0.8
United States	2,136	0.02	5.8	2.0	2.0	0.7
India	3,329	0.02^	5.7	2.3	2.7	0.8
Taiwan	1,125	0.02	4.6	1.9	2.4	0.6
Romania	1,082	0.02	5.7	2.7	2.8	0.7
Philippines	1,187	0.02	6.8	2.7	2.5	0.8
Brazil	1,199	0.01	5.3	2.8	2.7	0.6
Ghana	1,552	0.01	5.4	2.7	1.9	0.6
Japan	1,674	0.01	5.6	1.9	1.9	0.6
Peru	1,009	0.00	5.5	2.2	2.5	0.6
Haiti	1,940	−0.01	2.7	2.4	1.9	0.6
Turkey	1,368	−0.01	6.4	2.4	2.4	0.8
Australia1404	−0.02	5.3	2.0	1.9	0.6	
Ecuador	1,139	−0.03	5.6	2.5	2.4	0.6
Georgia778	−0.03	5.6	2.2	2.2	0.7	
Palestine	720	−0.03	6.0	2.3	2.4	0.7
Ukraine	1,500	−0.03	5.5	1.9	2.4	0.6
Poland	741	−0.03	5.5	2.3	2.3	0.5
Russia	1,441	−0.04^	5.4	2.1	2.5	0.6
Tunisia	696	−0.06^	5.6	1.8	2.6	0.8
Algeria	1,041	−0.08^	6.0	2.1	2.0	0.8
Trinidad and Tobago	561	−0.08^	6.4	2.4	1.8	0.7
Hong Kong	974	−0.08**	5.4	1.7	2.1	0.6
Thailand	1,187	−0.09**	5.9	2.2	2.1	0.7
Azerbaijan	991	−0.10**	5.9	2.0	2.2	0.6
Uzbekistan	777	−0.19***	6.5	2.1	2.5	0.9
Estonia	1,254	−0.16***	5.4	1.9	2.1	0.6
Egypt	1,523	−0.22***	6.1	2.3	3.5	0.6
**TOTAL**	**66,974**	0.03***	5.7	2.4	2.3	0.7

ID-Auth Beta = standardized beta for conservative ideology-authoritarianism relationship; both measures standardized within-nation. For ID-Auth Beta, Positive scores = right-wing authoritarianism more prevalent; negative scores = left-wing authoritarianism more prevalent. Means and SDs are unstandardized scores.

*** *p* < = 0.001;

** *p* < = 0.01;

* *p* < = 0.05;

^ *p* < = 0.10.

First, results averaged across nations were consistent with the Multilevel Modeling analyses: There was a small, but statistically significant, positive relationship between authoritarianism and conservative ideology worldwide, *beta*[66974] = 0.03, *p* < 0.001.

However, not only was this relationship negligibly small, within-country analyses clearly showed much variability across the world in the ideology-authoritarianism relationship. As Figure 4 and Table 6 reveal, many nations – particularly those in Western Europe and South America – showed positive and statistically significant relationships between political conservatism and authoritarianism. However, as the dark blue on Figure 4 and the bottom portion of Table 6 reveal, many nations showed positive and statistically significant relationships of authoritarianism with political *liberalism* (as indicated by the negative relationships between the political conservatism scale and authoritarianism measurement).

Importantly, Table 6 also reveals that these differences across nations in their propensity for authoritarianism to lean left or right are not likely an artifact of mean or SD differences across nations. Indeed, using nation as the unit of analysis (*n* = 54), the correlations between the ideology-authoritarianism *beta* and (a) country-level mean authoritarianism, (b) country-level SD for authoritarianism, (c) country-level mean ideology, and (d) country-level SD for ideology were all non-significant (*r*’s range from −0.25 to 0.09), and that was also true if one considers the ideology-authoritarianism effect as an absolute value (*r*’s range from −0.15 to 0.11). These additional results suggest there is real (and not artifactual) variability across countries in their likelihood of showing a conservatism-authoritarianism link.^10^

To understand part of this variability, we compared Western democracies with a region long influenced by more authoritarian left-wing ideology (Eastern Europe and former Soviet Republics). Specifically, after standardizing both authoritarianism and ideology within-nation, we ran a regression with National Context (Western Democracies versus Eastern Europe/Former Soviet Republics) and Ideology predicting Authoritarianism. Consistent with expectations, a National Context X Ideology interaction emerged (interaction *beta*[22,673] = −0.10, UCI = −0.13, LCI = −0.07, *p* < 0.0001).^11^ This interaction resulted from a significantly positive relationship between conservatism and authoritarianism for those living in Western democracies (*beta* = 0.09, *p* < 0.0001), but little to no relationship for those living in Eastern Europe/Soviet Republics (*beta* = −0.01, *p* = 0.14).

These results reveal that authoritarianism is present on both the right *and* the left side of the political spectrum around the world. Using two different methods of estimating the average effect across 54 nations (and over 66 thousand persons), we found that the overall relationship between conservative ideology and desire for authoritarian government is very small. Further, in many nations, authoritarians were significantly more likely to occur on the *left* side of the political spectrum (see the bottom portion of Table 6). It is noteworthy that a right-leaning correlation between ideology and authoritarianism has been interpreted as evidence of *right-wing* authoritarianism (Nilsson and Jost, 2020); thus, applying an equal and fair scientific standard, it is reasonable to interpret a left-leaning correlation between ideology and conservatism as evidence of *left-wing* authoritarianism. Given this, the left-leaning relationships reported at the bottom of Table 6 suggest clear (and statistically significant) evidence for left-wing authoritarianism in multiple nations. Additionally, the conservatism-authoritarianism relationship is stronger on average in contexts where one might expect it to be stronger (Western democracies), and weaker on average in contexts where one might expect it to be weaker (Eastern Europe/former Soviet Republics). In corroboration with Studies 1–11, these data suggest that left-wing authoritarianism is more of a reality than a myth.

## 8. General discussion

Is left-wing authoritarianism a viable construct that predicts important real-world phenomena? Across 12 studies spanning over 8,000 participants in the U.S. and over 66,000 participants worldwide, our data consistently reveal the answer is *yes*. These data reveal that (1) both liberal and conservative American participants identify a large number of left-wing authoritarians in their everyday lives (Study 1), and (2) both liberal and conservative participants rate a common Left-Wing Authoritarianism scale as measuring authoritarianism (Study 2). Further, this same LWA scale (3) consistently predicts key phenomena that major authoritarianism theories suggest it should predict, including (3a) threat sensitivity (Studies 3–6), (3b) restrictive communication norms (Study 7), (3c) negative ratings of minority groups (Studies 8–10), and (3d) dogmatism (Studies 10 and 11). Further, we used multiple methods to help overcome the double-barreled measurement problem inherent in *any* authoritarianism measurement, including controlling directly for ideology (Studies 3–11) and performing analyses only on liberals (Studies 3–11). Finally, we (4) found evidence of left-wing authoritarianism in an expansive world-wide sample (Study 12). Each of these approaches has offsetting strengths and weaknesses, and yet they all point to the same conclusion: This wide array of triangulating evidence provides consistent support for the idea that left-wing authoritarianism is indeed a widespread everyday reality.

Below, we place this array of evidence into the existing literature on authoritarianism and ideology, discuss limitations of our work, and offer a brief set of concluding thoughts.

### 8.1. The authoritarianism debate

The present studies have multiple implications for the ongoing debate about the nature of authoritarianism. Nilsson and Jost (2020) have argued that prior evidence based on Conway et al.’s (2018) LWA scale was due to its overlap with liberal ideology, and thus it did not provide empirical evidence of liberal authoritarianism. The issue raised by this critique is important. What *do* more focused empirical tests – tests based in long-accepted scientific practice – reveal? Our multi-method evidence here suggests that, in fact, the scale *is* measuring something beyond *mere* liberalism. Almost all key effects across Studies 3–11 remain when controlling for political ideology. Further, in a similar fashion, almost all key effects remain within-liberals: Thus, when comparing liberal authoritarians to liberal non-authoritarians, high-LWA persons show conceptually-expected correlations. As a result, the scale differentiates one kind of liberal from another kind, and thus cannot be reduced to *mere* ideology.

This array of evidence overwhelmingly suggests that, contrary to critics’ claims, there is something beyond mere ideology captured by the LWA scale. What *is* that *something beyond*? Consistent with a long line of research on RWA, by far the most parsimonious answer to that question is that the *something beyond* is *authoritarianism*. And indeed, using standard content validity approaches also used in other authoritarianism work (e.g., Funke, 2005; Dunwoody and Funke, 2016), Study 2 showed that participants evaluate the items in Conway’s LWA scale as measurements of *authoritarianism.* This strong empirical evidence is echoed in the judgments of researchers Fasce and Avendaño (2020, p. 3), who commented that the items on Conway et al.’s LWA scale “are not merely statements of liberal ideology; they univocally reflect an extremely authoritarian attitude, opposed to liberal commitments such as equality among citizens, freedom of expression, and tolerance toward political and cultural diversity.”

Taken together, this array of triangulating evidence points to the conclusion that – as is the case for the scientific consensus on the Altemeyer RWA scale on which it was based – Conway et al.’s LWA scale is a valid measurement of authoritarianism.

### 8.2. Limitations

Like all studies, the present study has limitations. First, although employing much larger and more diverse samples than most previous work on authoritarianism, Studies 1–11 (like much prior authoritarianism research) are nonetheless limited to the United States and should not be taken to generalize beyond that region.

Further, as other researchers have noted (Nilsson and Jost, 2020), the Conway et al. (2018) scale on which Studies 2–11 are based is not perfect. However, essentially all critiques of individual items on the scale hinge on the argument that these items do not measure anything beyond left-wing ideology.^12^ As such, all these smaller critiques are best addressed with triangulating empirical evidence that the whole collection of items – used in the way originally intended by the authors of the scale, as a total summative measure – is in fact capturing something beyond mere ideology. Evidence that the whole scale is valid suggests at a minimum that the collection of items as a whole is valid – and thus directly suggests there is no *systemic* problem with items interfering with the validity of the scale. It is just that kind of whole-scale validity evidence that has been supplied across multiple studies in the present package. This empirical approach mirrors the approach in other domains when critiques arise of the empirical validity of particular theoretical constructs (e.g., Banaji et al., 2004).

However, we acknowledge that Conway et al.’s (2018) LWA scale, like all scales, is not perfect and thus does of course have room for improvement (Conway, 2020). But saying a scale is *imperfect* is not the same as saying a scale is *invalid*. *All* measurements contain imperfections and all studies contain messiness, and yet that should not deter us from bigger-picture research conclusions (Cooper, 2016). Thus, we acknowledge the facts that (a) like virtually every scale, the LWA scale could be improved, and (b) as a scale designed to parallel the most widely-used RWA scale, it inherited some of that scale’s weaknesses. However, this lack of perfection should not be confused with the larger, big-picture issue of the degree that it can be construed as a valid measurement of left-wing authoritarianism. The overwhelming amount of evidence across multiple studies speaks clearly: It can be accurately viewed as a measurement of left-wing authoritarianism.

### 8.3. Concluding thoughts

Recent evidence has revealed a need for balanced evaluations of potential symmetries and asymmetries related to political ideology (e.g., Duarte et al., 2015; Jussim et al., 2015, 2016; Crawford, 2017; Frimer et al., 2017; Proch et al., 2018; Ditto et al., 2019; Eadeh and Chang, 2019; Fiagbenu et al., 2019; Clark and Winegard, 2020; Honeycutt and Jussim, 2020). Using a multi-method approach spanning multiple content areas, validity types, statistical controls, and scale types, the present results consistently show that, just as right-wing persons are sometimes authoritarian, left-wing persons may also be similarly authoritarian. Taken together, this large array of evidence suggests that left-wing authoritarianism is more of a reality than a myth.

## Data availability statement

The raw data supporting the conclusions of this article will be made available by the authors, without undue reservation.

## Ethics statement

The studies involving human participants were reviewed and approved by Institutional Review Board, University of Montana. The patients/participants provided their written informed consent to participate in this study.

## Author contributions

LGC, AZ, LC, and JM designed the studies, administered the experiments, and conducted the data analysis. LGC, AZ, LC, JM, and EV wrote and revised the manuscript. All authors contributed to the article and approved the submitted version.

## Funding

This work was supported by the National Cancer Institute at the National Institutes of Health [Grant Number R15CA186247; Conway PI].

## Conflict of interest

The authors declare that the research was conducted in the absence of any commercial or financial relationships that could be construed as a potential conflict of interest.

## Publisher’s note

All claims expressed in this article are solely those of the authors and do not necessarily represent those of their affiliated organizations, or those of the publisher, the editors and the reviewers. Any product that may be evaluated in this article, or claim that may be made by its manufacturer, is not guaranteed or endorsed by the publisher.

## Footnotes

^1^Further, their measurement of authoritarianism was poor on multiple levels. In addition to including only dichotomous responses, it was loaded with conservative content issues, such as those involving obedience to parents and approaches to single parenting. It further included two items related to general cynicism/trust that are conceptually orthogonal to belief in specific authority figures.

^2^We can only speculate as to the reason for the discrepancy between these two measures. Possible explanations include (1) the same authoritarian could appear across multiple categories (e.g., one authoritarian might be a co-worker and a friend), or (2) participants might be biased in their reporting of the overall score, such that it tends to underestimate the actual number when all authoritarians are added together. We cannot determine from our data which of these two is the correct explanation, although the nearly-identical correspondence of the parallel measures for the most authoritarian category suggest that participants’ scores more generally line up in this regard (and thus perhaps might suggest the first, and not the second, explanation). However, the larger point remains – regardless of the measure used, participants reported a substantial amount of left-wing authoritarianism.

^3^A set of 2 (Participant Ideology: Liberal or Conservative) X 2 (Type of Authoritarian Considered: Liberal versus Conservative) Mixed-Model ANOVAs (with Participant Ideology as the Between Subjects variable and Type of Authoritarianism as the Within-Subjects variable) revealed an interaction between ideology and authoritarianism type on mean number of authoritarians and most authoritarian person summary scores, *F*’s > 6.5, *p*’s < 0.02. Consistent with an Authoritarian Norm Fit Model that posits liberals in the U.S. are more motivated than conservatives to avoid authoritarian ingroup labels (Conway et al., 2020b; 2021b), the pattern predictably suggests that, in the main, liberals are more likely (versus conservatives) to identify authoritarians in outgroups than ingroups. However, this is orthogonal to the main point of Study 1.

^4^We also did analyses using a more stringent criterion for considering someone a liberal. Participants answered two categorical ideology questions as well in Study 1, and for this supplementary analyses, we considered someone a liberal if they self-identified as both a "liberal" and a "democrat." While this predictably yielded fewer liberals overall than those reported in the text, the substantive results reported in the main text are essentially identical in their import to these supplementary analyses. No matter how they are sliced, the present data reveal a consistent story: Participants of all political persuasions reported a lot of evidence of left-wing authoritarianism.

^5^Conway et al. (2022) used these and other data to construct a four-item short form of the LWA scale that has excellent validity and is correlated with the two existing major long-form LWA scales.

^6^Some research suggests that conservatives care more about physical threat – and especially disease/disgust based threat (e.g., Terrizzi et al., 2013) – than liberals do (see Crawford, 2017, for a review). Thus, there is reason to suspect that such threats are not ideologically neutral. And indeed, in a preliminary study (*n* = 467) showing results identical to the pre-registered Study 3 presented here, we found that these ecological threats lean *slightly* towards conservatives. That was also true in the present study. However, this would actually work against LWA showing a relationship with threat in the present study – and part of the reason why we controlled for ideology directly.

^7^These data were drawn from Sample 1 of Costello et al. (2022). All analyses presented here are novel. We are grateful to Thomas Costello for sharing these data with us.

^8^We additionally re-computed the indirect path analyses from Study 6 within-group and found the exact same set of results reported on the whole sample. Specifically, looking only at liberals from Study 6 revealed support for an LWA➔Perceived Trump Threat➔Democratic Candidate Support Path, indirect effect = 0.08 (LCI = 0.02, UCI = 0.17), *p* = 0.014. Similarly, within-group evidence revealed support for an LWA➔Perceived Trump Threat➔Oppose Trump Path, indirect effect = 0.07 (LCI = 0.02, UCI = 0.15), *p* = 0.018.

^9^While none of these items is entirely ideologically-free (and it is possible that the military item might skew the results towards RWA), of these three items, the most clearly ideologically-free item is item one (about a desire for a strong leader). Thus, we further computed all analyses on this item only. Those analyses are identical, both descriptively and inferentially, as those reported for the whole scale.

^10^Bolstering this case, we further divided persons up categorically into those that leaned left (below the midpoint of the scale) and those that leaned right (above the midpoint of the scale). As in Conway et al. (2018), analyses with this categorical variable suggests that our findings are not likely due to a correlational sleight-of-hand that is driven by generally higher levels of conservatism world-wide. Indeed, not only is this sample only slightly right-leaning (ideology scale mid-point = 5.5; worldwide *M* = 5.66), making such an alternative explanation unlikely, but also the highest level of authoritarianism in the world in this sample occurred for *liberals* in Egypt (Egypt Liberal Authoritarianism *M* = 3.64; worldwide Authoritarianism *M* = 2.32).

^11^This interaction remained significant (and in the same direction) when using the categorical left/right distinction as the ideology measurement (*F* = 9.98, *p* < 0.002).

^12^We pause to note that, if this were true, as a measurement of *liberalism* the LWA scale would paint an excessively unflattering portrait of liberals – a portrait that would go against much theorizing about liberalism. Fortunately, our own data suggest this is *not* true, and thus the scale can rather be viewed as a measurement of a particular kind of liberal, and not a more general measurement of liberalism.
