# Supplementary material for: Is the myth of left-wing authoritarianism itself a myth?
Source: Front Psychol. 2023 Feb 8;13:1041391. doi: 10.3389/fpsyg.2022.1041391 (PMC9944136; doi:10.3389/fpsyg.2022.1041391)
Supplement: Supplementary file 1 [file Data_Sheet_1.pdf]

Is the Myth of Left-Wing Authoritarianism Itself a Myth?

Expanded Supplementary Material

This supplement contains expanded discussions of arguments, methods, study-specific results, and additional information referenced in the main text.

The Basis of Skepticism About LWA: Expanded Arguments

Altemeyer's LWA Evidence

Historically, much of the skepticism about LWA can be traced to Altemeyer's work on the construct. After constructing an LWA scale purported to be parallel to his RWA scale, he found almost no evidence of LWA, indeed famously reporting that high-LWA persons were "as rare as hen's teeth in my samples" (Altemeyer, 1996). This lack of evidence from Altemeyer has often been one of the key arguments cited to suggest that LWA does not exist. For example, in their highly-cited paper on ideology (one of the most influential papers in the history of the topic, having been cited over 4,400 times on Google Scholar), Jost et al (2003)'s dismissal of LWA as a construct prominently featured Altemeyer's research. Indeed, one does not have to read far into Jost et al.'s (2003) classic paper to see how much of their own view of the rigidity of the right is based on Altemeyer's work. For example (Jost et al., 2003; p. 353):

> *"Altemeyer (1998) concluded, 'I have yet to find a single "socialist/Communist type" who scores highly (in absolute terms) on the [Left-Wing Authoritarianism] Scale. Shils may have been right about his era, but the "authoritarian on the left" has been as scarce as hens' teeth in my samples' Evidence suggests that dogmatism has been no more useful than the construct of authoritarianism for identifying rigidity of the left…"*

This reliance on Altemeyer's evidence by skeptics of LWA would be rather alarming even if the evidence was particularly compelling, because it is based on only a few samples from one cultural context. However, it is especially troubling because upon

closer inspection, Altemeyer's evidence is itself deeply flawed even in describing that one cultural context. For example, although his LWA questionnaire was intended by his own stated goal to be parallel to his RWA scale, it is clearly not parallel in multiple large-scale ways. Specifically, Altemeyer's LWA scale adds two highly salient item features not present in the RWA scale.

First, Altemeyer's LWA scale requires participants who score high on the questionnaire to support a revolution to overthrow the established government. In fact, twenty of the twenty-two items on Altemeyer's LWA scale reference a revolutionary movement. For example: "The members of the Establishment deserve to be dealt with harshly, without mercy, when they are finally overthrown." By contrast, *none* of the items on any of Altemeyer's RWA scales makes a single reference to violent upheavals overthrowing the establishment. Second, whereas the RWA and LWA scales both use vigorous authoritarian, negative, dogmatic, and punitive language, only the LWA items leave absolutely no doubt that the endorsement of violence is explicitly required to score high on the scale. For example: "The conservative right-wing Establishment will never give up its power peacefully, so a revolutionary movement is justifying in using violence to crush it."

Thus, Altemeyer's RWA and LWA scales are *not* parallel in very important ways. Indeed, whereas Altemeyer's RWA scale reads like a measure of general authoritarianism, Altemeyer's LWA scale reads like a screening instrument for joining a violent revolutionary group that wants to overthrow the government. As a result, the fact that few people scored high on Altemeyer's LWA scale tells us little about left-wing authoritarianism. Rather, it simply tells us the obvious fact that, whether left-wing or

right-wing, few people want to endorse, let alone join, a violent military movement designed to attack and overthrow something else. However, historically, some of academia's dismissal of LWA was based on this clearly flawed evidence from Altemeyer.

These flaws were partially addressed by Conway and colleagues (2018) when they used a version of Altemeyer's RWA scale to create a more parallel LWA scale. These researchers thus bypassed the issue of the revolution/aggression asymmetry by mirroring Altemeyer's RWA scale – which did not contain such language – to create a new LWA scale. Unlike Altemeyer's original LWA scale, this new LWA scale kept much of the same authoritarian language as the original scale – only it replaced conservative authorities with liberal authorities. When this more parallel scale is used to measure LWA, evidence of LWA is manifest, often at effect sizes similar to those for RWA (Conway et al., 2018; Conway & McFarland, 2019; Conway et al., 2021; Fasce & Avendaño, 2020). Further, Conway et al's LWA scale predicts (1) voting intentions in the 2016 election (Conway & McFarland, 2019), (2) distrust of opponents, reactance, and support for divisive behaviors (Conway, Houck et al., 2021), and (3) a reactive stance against reward for application (Fasce & Avendaño, 2020).

The Double-Barreled Nature of Authoritarianism

A second basis of skepticism, although arguably historically less important, is more scientifically plausible. That skepticism is based in the fact that Left-Wing Authoritarianism is a *double-barreled* construct. Because it contains both ideological (left-wing) content *and* authoritarian content, it can be a challenge to disentangle the degree that LWA effects are the result of *authoritarianism* or the result of *ideology*. For

example, Honeycut and Jussim (2020) wondered about Conway et al's (2018) LWA evidence: "Of course, this problem is itself confounded with the measurement problem—is anyone shocked that conservatives score higher than liberals on a *rightwing* authoritarianism scale, whereas liberals score higher than conservatives, on a *leftwing* authoritarianism scale?" The implication is clear: Because LWA simultaneously measures both ideology and authoritarianism (that is, because it is *both* left-wing *and* authoritarian), how can we be sure that any results we find are truly about *authoritarianism* per se? Perhaps those findings could be explained without considering *authoritarianism* at all.

This is a perfectly reasonable measurement concern. However, it is not a problem specific to LWA measurement: It is a problem likely inherent in *any* authoritarianism measurement. Right-wing authoritarianism also has both ideological content (right-wing) and authoritarianism embedded into the construct. Indeed, no matter what you put in the blank, [blank] authoritarianism will be double-barreled in some sense, because it will have both some content (the "[blank]") and also authoritarianism built in.

Importantly, this is not a problem manufactured by authoritarianism researchers; rather, it is a measurement challenge likely *inherent in authoritarianism itself.* Authoritarianism is almost certainly expressed primarily in domain-specific ways (Conway et al., 2020), and as a result, all authoritarianism questionnaires must in some sense (either explicitly or implicitly) answer the question "authoritarianism to *WHAT*?" Few people walk around acknowledging every authority figure's legitimacy, and even when specific authority figures are not stated on a questionnaire, they will likely be imagined by respondents (see, e.g., Frimer et al., 2014). Indeed, the way the construct is

historically defined unequivocally means that those high in authoritarianism actually draw sharper lines denouncing *outgroup* authority figures. Persons high in authoritarianism are especially likely to adhere to one authority and group and, as a result, to *reject* other authorities and groups. Right-wing authoritarians adhere to religious and conservative authorities – but they are especially likely to reject scientific and liberal authorities. In much the same way, we would expect left-wing authoritarians to adhere to a *different* set of authorities than right-wing authoritarians, and to *reject* a different set of authority figures as well. For example, left-wing authoritarians would be more likely to adhere to their liberal college professors while rejecting their religious parents.

And yet this state of things does not invalidate that authoritarianism is an important construct with real-world consequences. The fact that a right-wing authoritarian clings to religious authorities but rejects scientific authorities does not make them less authoritarian. Likewise, the fact that a left-wing authoritarian clings to liberal authorities but rejects conservative ones does not make them less authoritarian.

However, in both cases, it does pose a measurement challenge: How are we to separate the ideological parts from the authoritarian parts? In the case of LWA, how do we separate *liberal non-authoritarians* from *liberal authoritarians*? This is, in fact, one of the primary challenges critics have levied at LWA research (Nilsson & Jost, 2020). In the next section, we address this exact question by drawing on established methods in social psychology for this specific measurement challenge. Then, across multiple studies, we provide evidence that suggests it is liberal *authoritarianism*, and not just *liberalism*, that is involved in our key effects.

Scientific Standards of Evidence for LWA: Expanded Arguments

When approaching any scientific issue, it is important to apply the same standards of evidence on all sides of a discussion (Tetlock, 1994). In the present case, this issue is pertinent in several important ways.

Equivalent Standards for RWA and LWA

Altemeyer's RWA scale – on which Conway et al's (2018) LWA scale was based – has historically been, and still is, by *far* the most extensively-used measurement of the right-wing authoritarianism construct. For example, an empirical study (Conway et al., 2018) showed that 79% of the scales from recent research that measured RWA used a version based on Altemeyer's scale – either Altemeyer's original RWA scale (62%) or the short version constructed by Zakrisson (2005; 17%). Indeed, even since 2018, Altemeyer's RWA scale has continued to be widely-used in top journals (including *Journal of Personality and Social Psychology* and *Personality and Social Psychology Bulletin*), and in most cases used in a manner that assumes it is measuring *authoritarianism* and not just ideology (see Conway et al., 2020).

The scientific consensus thus overwhelmingly favors the conclusion that Altemeyer's scale measures *authoritarianism* above and beyond ideology. Indeed, it is the past and present scientific standard in the field for measuring authoritarianism, and decades of scientific knowledge about the construct – knowledge often accepted as axiomatic – has been built upon it. And for good reason: As we will see in Study 2, it is an excellent face valid measurement of authoritarianism. Thus in this case the scientific consensus is correct: Altemeyer's RWA measurement, though not without flaws, is a good measure of general *right-wing* authoritarianism.

Given this, it is important that we apply the same standards of evidence to judging LWA that have been used to arrive at that conclusion for RWA. Consider the case of Conway et al.'s (2018) LWA scale used in the present set of studies. Unlike Altemeyer's own LWA scale, Conway et al's LWA scale mirrors the language of the most-validated and widely used RWA scale (Altemeyer's RWA scale that represents the historical and current scientific consensus on the topic). As a result, Conway et al's LWA scale possesses high content validity as a measure of authoritarianism.

Consider that participants who score high on the LWA scale agree that (italicized words are direct quotes from the LWA scale): *Our country needs a mighty leader*, that the leader should *destroy* opponents, that people should *trust the judgment of the proper authorities*, should avoid listening to *noisy rabble rousers in our society who are trying to create doubts in people's minds,* should *put some tough leaders in power who oppose those values and silence the troublemakers,* should *smash* the beliefs of opponents, that *what our country really needs is a strong, determined leader who will crush the evil*, that society should *strongly punish those* they disagree with, deny that their opponents have a right to *be wherever he or she wants to be*, and support the statement that the country would be better off if certain groups *would just shut up and accept their group's proper place in society*. These items hit all of the hallmarks of the consensus conceptualization of the *authoritarian* person. For decades, it has been assumed that if people agreed with those statements when the targets of authoritarianism were conservative and the outgroups were liberal, then they were indeed *authoritarians*. Therefore, if people agree with those statements when the targets of authoritarianism are *liberal* and the outgroups are *conservative,* we must – applying the same standard – also agree that they are

authoritarians. If we grant that someone saying they want to *put some tough leaders in power who oppose those values and silence the troublemakers* is authoritarian when referring to right-wing leaders, then we also have to grant that someone saying the exact same thing when referring to left-wing leaders is also an authoritarian.

Separating "Liberal" From "Liberal Authoritarian"

But of course, although important, content validity is just one aspect of validity. While LWA passes the content validity test (something we return to in Study 2 more explicitly), what standards can we use to solve the measurement challenge posed by a double-barreled construct? As we noted earlier, one of the difficulties with measuring authoritarianism is that it is inherently content- and value-based. Because authoritarianism focuses on submission to authorities (see e.g., Duckitt et al., 2010; Feldman, 2003), it is hard to consider the construct without considering *what* authorities people are submitting to.

In a presentation on this psychometric conundrum, Conway (2020) offered several solutions to the double-barreled measurement problem inherent in authoritarianism. All of these solutions are drawn from basic social psychological methods, and many of them have served as the basis for accepting RWA as a measurable construct. As a result, evidence along these lines should apply equally to LWA. We cover these solutions next.

Use ideologically-neutral scales. Because the authoritarianism construct is inherently about commitment to specific authorities and rejection of others, it is very difficult, if not impossible, to write an ideologically-neutral authoritarianism scale. However, some language is more ideologically-neutral than others. In Study 12, we use

one such scale that has less ideologically-loaded language to demonstrate the existence of LWA around the world.

Experimental approaches: Use parallel ideologically-loaded scales that manipulate ideological content but hold authoritarian language constant. In classic experimental research paradigms, researchers attempt to isolate specific variables while holding everything else constant. Thus, one method for isolating authoritarianism is based in basic experimental logic and involves writing equivalent scales for RWA and LWA. To the degree that (1) all words related to authoritarianism for both RWA and LWA are face valid measurements of authoritarianism, (2) all words related to authoritarianism are essentially identical for both RWA and LWA, and (3) the only thing varied across scales is the *type of authoritarian submitted to,* we can infer that (4) similarities in effects across scales are reasonably attributed to authoritarianism, whereas (5) differences in effects across scales are reasonably attributed to the type of authority figures (or other aspects associated with conservative versus liberal content).

We employ this method in Studies 3-11. It is worth noting, however, that many times one would expect different covariates of scores on the two types of authoritarianism questionnaires for theoretically-hypothesized reasons (e.g., when the dependent measure has ideologically-loaded content). As a result, we view it important to use this method in tandem with the other methods described below.

Use ideologically-loaded scales that control for ideology. The most typical way to isolate the effect of one variable over and above another variable is to control for the second variable (Hayes, 2013; Preacher, 2015; Darlington & Hayes; 2017 Hayes & Rockwood, 2020). This has a straightforward application to both RWA and LWA: If

scientists want to isolate the "authoritarian" part of "X" Authoritarian, they can statistically control for "X." Thus, if we aim to isolate the "authoritarian" part of left-wing authoritarianism, we can control for participants' ideology ("left-wing/right-wing"). While this method is not perfect, if effects of LWA remain after controlling for ideology, it nonetheless suggests that something *beyond* liberal ideology is accounting for those effects. As we will see across Studies 3-11, we believe that the *something beyond* is best categorized as *authoritarianism.*

Use ideologically-loaded scales *within* the focal group. A complementary method for separating out ideology from authoritarianism is to perform within-ideological group analyses (see Wronski et al., 2018, for an example). This method has the disadvantage of truncating the range and discarding available data. But on the other hand, it gains a simple heuristic advantage: If one, for example, finds an effect of RWA *only within conservative persons*, then it suggests that the effect is not driven by ideology (since that is held roughly constant in within-ideological group analyses), but by authoritarianism. Similarly, if one finds an LWA effect *only within liberal persons*, then this suggests the effect of LWA is driven by authoritarianism and not left-wing ideology. In other words, one way to separate liberal authoritarians from liberal non-authoritarians is to look only at liberals.

To accomplish this, in Study 12 we performed further analyses that analyzed all key LWA effects by looking within liberal groups. As we will see, both across-group and within-group analyses overwhelmingly support the idea that there is something beyond mere ideology at play here; that *something beyond* is, we believe, best described as authoritarianism.

The Present Studies

Informed by this set of standards, we below present a wide array of novel evidence concerning the real-life prevalence and importance of LWA within (Studies 1-11) and outside (Study 12) the USA. In Study 1, we ask participants about their own perceptions of LWA persons in their lives (and discover that participants on both sides of the political spectrum, to a surprising amount, identify a descriptively important number of left-wing authoritarians in their own lives). In Study 2, we ask participants to judge the degree that items from Conway et al's (2018) LWA questionnaire are measurements of authoritarianism (and discover that they are rated as good measurements of authoritarianism). In Studies 3-11, we evaluate the degree that persons scoring high on this LWA scale show the properties that prominent authoritarianism theories suggest an authoritarian person should have. Studies 3-6 reveal that persons high in LWA show heightened sensitivity to threat. Study 7 shows high-LWA persons have more support for a restrictive social norm. Studies 8 and 9 reveal that high-LWA participants show more negative ratings of African-Americans and Jews, while Studies 10 and 11 reveal that high-LWA participants show higher scores on rigidity measurements such as dogmatism and need for closure. Across all studies 3-11, these effects occur when controlling for political ideology. These studies reveal that LWA and RWA show similar effects across comparable measures, and further shows that the majority of the reported effects hold when looking only *within* liberals, thus revealing these effects are about liberal *authoritarianism* and not just *liberalism* in general. Study 12 uses a common authoritarianism questionnaire from the World Values Survey to provide evidence of Left-Wing Authoritarianism around the globe.

Study 1: Expanded Methods, Results, and Discussion

Methods

The focus of Study 1 was purely descriptive in that it evaluated the everyday occurrence of authoritarianism in lay populations. For Study 1, we gave participants categories of persons (e.g., family, co-worker) and asked them to identify authoritarians in their lives on both sides of the political spectrum.

Participants. Four hundred forty-one U.S. adults were recruited using Amazon's *Mechanical Turk (MTurk)*. *MTurk* has been validated for use as a representative sample for research related to politics and political ideology (see, e.g., Clifford, Jewell, & Waggoner, 2015, Kennedy et al., 2018) and generally shows similar results as other samples (e.g., Houck et al., 2014). Further, *MTurk* has been validated for use in work on authoritarianism (Choma & Hanoch, 2017; Ludeke et al., 2018). As a result, *MTurk* is an excellent choice for work on U.S. authoritarianism. The sample was 55% female, had an average age of 38, and was slightly left-leaning politically (4.3 on a political conservatism scale with 4.5 as the midpoint).

Initial Directions to Participants. Participants were randomly assigned to one of two different sets of directions. In the first set of directions (*Definition Given*), we gave participants a standard definition of authoritarianism. This definition was drawn from prior work suggesting that authoritarianism has three primary aspects (e.g., Altemeyer, 1996; Feldman, 2001; Duckitt et al., 2010):

In this study, we are interested in your views of authoritarianism and authoritarian people. First, we will describe what we mean by authoritarianism. Authoritarianism involves a need for group cohesion and the subordination of individual

autonomy to the group and its authority. Authoritarian persons can potentially be a part of any group – to be considered authoritarian, it does not matter to what group they belong. Rather, regardless of the type of group, authoritarian persons generally have the following characteristics: Authoritarian persons have attitudes favoring obedience to group leaders and authorities. Authoritarian persons have attitudes favoring harsh, coercive social control (e.g., punishment, aggression). Authoritarian persons have attitudes favoring conformity to established group norms and values.

Participants in the other condition (*Definition Generated*) were given no definition, but instead were asked to generate their own definition:

In this study, we are interested in your views of authoritarianism and authoritarian people. First, we would like to get your own thoughts on what you think an authoritarian person is. So in the space below, please type a description of what you think someone who is authoritarian would be like.

Participants in this condition generally seemed to understand the authoritarianism construct and, as we will see below, our key interpretations were unaffected by the directions manipulation.

Authoritarianism Measurements: Mean Number. Participants then completed a series of parallel measurements asking them how many authoritarians they could identify in their lives across various categories for both liberals and conservatives. For example, participants were asked: *In your family (including all extended family), how many authoritarians can you think of that are politically liberal?* They were then given options 0, 1, 2, 3, 4, 5, 6-10, and more than 10.

A parallel question was asked for how many family members were authoritarians

who were politically conservative: *In your family (including all extended family), how many authoritarians can you think of that are politically conservative?*

Using this method, we asked participants to identify left- and right-wing authoritarians across four different categories: Family, Friends or Acquaintances, Co-Workers, and News/TV/Movie/Sports Personalities. We further asked participants to simultaneously consider (and report the overall number of) *all* the authoritarian people they could identify who were politically liberal and politically conservative (scale options = 0, 1, 2, 3, 4, 5, 6-10, 11-15, and greater than 15). To convert these to a single number per variable, when participants chose ranges, we entered the midpoint of the range (e.g., 6-10 became 8). When the “greater than” option was chosen, we added two to the end of the range to estimate the number.

We used two primary summary scores with complementary strengths and weaknesses. First, we used participants’ own report of the total number of authoritarians they knew on the right and the left (referred to in the tables as *TOTAL: REPORTED*). We further took the sum of all the four categories (referred to as *TOTAL: SUM*).

Authoritarianism Measurements: Most Authoritarian Person. Participants were then asked to consider the most authoritarian person they could identify in their life across each of the four categories. In each case, they were asked whether or not the representative *most authoritarian person* was *liberal*, *conservative*, or *neither/do not know*. Finally, participants were asked to think of the most authoritarian person overall in their lives and identify whether the person was liberal, conservative, or neither/do not know. We created an additional summary score conceptually identical to the summary for the mean number measurements by taking the average percentage across the four types of

authoritarians.

Participant Ideology. All participants further completed a standard two-item political conservatism scale, with items anchored by liberal/conservative and democratic/republican (e.g., Conway et al., 2012; Jost et al., 2008; Jost et al., 2003). In order to provide easy descriptive summaries, for Study 1 we converted this measurement to a dichotomous measure in a manner identical to prior research (Conway et al., 2016; Conway et al., 2018) by considering people above the mid-point *conservative* and people below the midpoint *liberal* (people right at the mid-point were dropped for all analyses including this variable; $n = 395$ for those analyses, with 244 liberals and 151 conservatives).

Results and Discussion

Although the primary purpose of Study 1 is to investigate the descriptive nature of participant perceptions of left-wing authoritarians, we present inferential comparisons for completeness. Descriptive results for all measures are presented in Tables 1 and 2. Because (as the tables reveal) the results tell the same story across all categories, for the sake of brevity, in this narrative we focus only on the overall summary scores.

We first tested the influence of our directions manipulation by running 2 (Definition Instructions: Definition Given versus Definition Generated) X 2 (Type of Authoritarian Considered: Liberal versus Conservative) Mixed-Model ANOVAs, with Instructions as the Between Subjects variable and Type of Authoritarianism as the Within-Subjects variable. Participants reported more authoritarians in the Definition Given condition than in the Definition Generated condition, F's > 8.7 , p's < .004. However, this did not significantly differ by type of authoritarianism, interaction F's <

.90, *p*'s > .34. Similarly, there was no significant interaction between the instructions manipulation and the type of authoritarianism on the two summary *most authoritarian person* measurements, *p*'s > .15. As a result, we subsequently dropped the instructions manipulation for ease of understanding; however, including it would not substantively change any of the conclusions drawn from Study 1 below.

Table I

Study 1: Identification of Left- and Right-Wing Authoritarians in Everyday Life

	**Liberal Authoritarians**	**Conservative Authoritarians**
**Mean Number of Authoritarians**		
Family	*1.7*	*2.8*
Friends	*2.4*	*2.8*
Co-Workers	*2.4*	*2.8*
News/TV/Movie/Sports	*5.1*	*6.0*
**TOTAL (SUM)**	**11.7**	**14.4**
**TOTAL (REPORTED)**	**7.8**	**10.3**
**Most Authoritarian Person**		
Family	*33%*	*57%*
Friends	*36%*	*48%*
Co-Workers	*27%*	*51%*
News/TV/Movie/Sports	*38%*	*50%*
**TOTAL (AVERAGE)**	**33%**	**52%**

**TOTAL (REPORTED)**	**34%**	**61%**

Notes: N = 441.

Consistent with prior assertions that right-wing authoritarianism is more prevalent than left-wing authoritarianism, participants reported significantly more right-wing authoritarianism for all summary measures of both mean number and most authoritarian person measures, all within-subjects F's > 19.0, p's < .001. However, more important to our present purpose, participants consistently identified a large number of left-wing authoritarians as well. As seen in Table 1, participants self-reported identifying 7.8 liberal authoritarians on average, and the sum total of the identified liberal authoritarians across the four categories was 11.7 authoritarians.[1]

Table II

Study 1: Identification of Left- and Right-Wing Authoritarians in Everyday Life, By Political Identification of the Participant

	Liberal Participants		Conservative Participants	
	**Liberal**	**Cons.**	**Liberal**	**Cons.**
**Mean Number of Authoritarians**				
Family	*1.6*	*2.6*	*1.8*	*3.1*
Friends	*2.5*	*2.6*	*2.4*	*3.2*
Co-Workers	*2.3*	*2.9*	*2.5*	*2.9*
News/TV/Movie/Sports	*4.0*	*6.6*	*6.7*	*4.9*

[1] We can only speculate as to the reason for the discrepancy between these two measures. Possible explanations include (1) the same authoritarian could appear across multiple categories (e.g., one authoritarian might be a co-worker and a friend), or (2) participants might be biased in their reporting of the overall score, such that it tends to underestimate the actual number when all authoritarians are added together. We cannot determine from our data which of these two is the correct explanation, although the nearly-identical correspondence of the parallel measures for the most authoritarian category suggest that participants' scores more generally line up in this regard (and thus perhaps might suggest the first, and not the second, explanation). However, the larger point remains – regardless of the measure used, participants reported a substantial amount of left-wing authoritarianism.

**TOTAL (SUM)**	**10.5**	**14.8**	**13.4**	**14.0**
**TOTAL (REPORTED)**	**6.6**	**9.0**	**11.3**	**8.5**
**Most Authoritarian Person**				
Family	*34%*	*57%*	*31%*	*60%*
Friends	*34%*	*52%*	*37%*	*53%*
Co-Workers	*23%*	*57%*	*34%*	*41%*
News/TV/Movie/Sports	*29%*	*60%*	*50%*	*37%*
**TOTAL (AVERAGE)**	**30%**	**56%**	**38%**	**48%**
**TOTAL (REPORTED)**	**24%**	**71%**	**49%**	**48%**

Notes: Total N = 395. Conservative participant n = 151; liberal participant n = 244.

As Table 2 indicates, this remains the case even when focusing only our liberal participants.[2] As seen in Table 2, liberal participants self-reported identifying 6.6 liberal authoritarians on average, and the sum total of the identified liberal authoritarians (identified by liberal participants) across the four categories was 10.5 authoritarians.[3]

Indeed, all the mean numbers for liberal authoritarians (both individual categories and summary scores) presented in Tables 1 and 2 are significantly different from zero using one-sample t-tests (p's < .001). More importantly, the descriptive statistics reveal that most people report identifying a substantial number of left-wing authoritarians in their lives. Further, even liberal participants report that 24% (or 30%, depending on the

[2] A set of 2 (Participant Ideology: Liberal versus Conservative) X 2 (Type of Authoritarian Considered: Liberal versus Conservative) Mixed-Model ANOVAs (with Participant Ideology as the Between Subjects variable and Type of Authoritarianism as the Within-Subjects variable) revealed an interaction between ideology and authoritarianism type on mean number of authoritarians and most authoritarian person summary scores, F's > 6.5, p's < .02. Consistent with an Authoritarian Norm Fit Model that posits liberals in the U.S. are more motivated than conservatives to avoid authoritarian ingroup labels (Conway et al., 2021; Conway, Zubrod, & Chan, 2020), the pattern predictably suggests that, in the main, liberals are more likely (versus conservatives) to identify authoritarians in outgroups than ingroups. However, this is orthogonal to the main point of Study 1.

[3] We also did analyses using a more stringent criterion for considering someone a liberal. Participants answered two categorical ideology questions as well in Study 1, and for this supplementary analyses, we considered someone a liberal if they self-identified as both a "liberal" and a "democrat." While this predictably yielded fewer liberals overall than those reported in the text, the substantive results reported in the main text are essentially identical in their import to these supplementary analyses. No matter how they are sliced, the present data reveal a consistent story: Participants of all political persuasions reported a lot of evidence of left-wing authoritarianism.

summary measure) of their most authoritarian persons are, in fact, liberal. In the present study alone, that would translate to (at a minimum) 58 extremely left-wing authoritarians identified by *liberal* participants. As a thought experiment, extrapolated to the U.S. population as a whole, these data would mean that tens of millions of people – including liberals – would identify a left-wing authoritarian as the most authoritarian person in their life. This would translate to literally *millions* of (very real) left-wing authoritarians in the U.S. presently, across all walks of life. This thought experiment would hold true even if one excludes the TV personality category that would likely include quite a bit of overlap across persons, and instead focus on categories where across-person overlap would be minimized. Thus, while the present data do suggest participants identify more right-wing than left-wing authoritarians, they also suggest that participants – even liberal participants – identify a meaningfully large number of left-wing participants in their lives.

Of course, we do not wish to *over*state the importance of these data. We recognize that *MTurk*, while a valid research resource, does not represent the whole population of the U.S. Further, participants can sometimes be wrong in their interpretations of other people in their lives. Rather, these data *suggest* that perhaps the debate about left-wing authoritarianism, so ardently discussed in academic circles, is less in evidence in lay populations. The average citizen may feel more confident that left-wing authoritarians exist – as they report knowing many of them.

Study 2: Expanded Methods, Results, and Discussion

Overview

Construct validity is a complicated and multi-faceted concept. At a most basic level, however, construct validity is a simple question: Does a questionnaire set measure

what is purports to measure? One of the most basic, direct, and important ways to determine if a scale measures what it is supposed to measure is to provide content judgments concerning whether or not scale items are measuring the key construct. Indeed, this method has been used in other authoritarianism work (e.g., Funke, 2005; Dunwoody & Funke, 2016). In Study 2, we thus provided such direct validity evidence by asking participants if items from commonly-used LWA and RWA questionnaires do, in fact, measure authoritarianism. To the degree that participants believe they do, this provides a piece of evidence (in a larger puzzle) that LWA is a real construct that is meaningfully measured by a recently-developed LWA scale.

Methods

Because Conway et al's (2018) LWA scale was purposefully designed to be parallel to a version of Altemeyer's (1996) RWA scale, we selected parallel items from each scale for this validity test. For a discriminant validity comparison group, we further selected items from a widely-cited Big 5 Personality inventory. In all cases, we asked participants to identify the degree that they believed that someone scoring high on an item would be an authoritarian person.

Participants. Four hundred seventeen U.S. adults (50% female, mean age = 38) were recruited using Amazon's *Mechanical Turk (MTurk)*. The sample was slightly left-leaning politically (4.2 on a political conservatism scale with 4.5 as the midpoint). Participants were randomly assigned to receive one of the three sets of parallel items described in more detail below: RWA, LWA, or De-Politicized LWA.

Instructions to Participants. All participants were first given the definition of authoritarianism used in Study 1's *Definition Given* condition, which contains a summary

of the widely-accepted three-aspect model of authoritarianism:

> *Authoritarianism involves a need for group cohesion and the subordination of individual autonomy to the group and its authority. Authoritarian persons can potentially be a part of any group – to be considered authoritarian, it does not matter to what group they belong. Rather, regardless of the type of group, authoritarian persons generally have the following characteristics:*
>
> > *Authoritarian persons have attitudes favoring obedience to group leaders and authorities.*
> >
> > *Authoritarian persons have attitudes favoring harsh, coercive social control (e.g., punishment, aggression).*
> >
> > *Authoritarian persons have attitudes favoring conformity to established group norms and values.*

Then participants read a description of their task:

> *Now we are going to present you with questions which may or may not measure authoritarianism. Your task is NOT to answer questions about your own agreement with the question. Rather, your task is simple: It is to judge whether or not the question presented is a legitimate measurement of an authoritarian person. Specifically, for each of the following questions, while considering the definition of authoritarianism (and each of the three characteristics mentioned above), we would like for you to state whether you think someone who answered "yes" to the question would be an authoritarian person, using the following scale: 1 = I cannot tell from someone's answer to this item whether that person is authoritarian or not. 2 = Someone who answered "yes" might be authoritarian,*

but it is not very clear. 3 = Someone who answered "yes" to this item is very likely authoritarian.

After that, participants were presented items (described below) for making judgments. Prior to each item, they were reminded of the nature of their task ("Imagining what it would mean if OTHER PEOPLE responded 'yes,' consider the following item:").

Selection of LWA and RWA items. From the LWA and RWA scales, we selected all the pro-trait items (see Appendix for all items) for this validity test. (Note that because the scales use parallel wording, this means that each scale had the same set of "base" items). This decision was based on the nature of the task: We deemed that trying to ask participants to "reverse" the item to see if someone who scored low would be high in the authoritarianism trait might in this instance be an unnecessarily difficult task – and that this fact would subsequently make interpretation difficult. As a result, throughout we focused only on pro-trait items from all scales. This left 10 items each to be used in the validity test for both LWA and RWA.

For LWA, we further created a set of De-Politicized LWA items by removing all clearly political language (such as "liberal" and "conservative") and, when necessary, replacing politicized words with generic alternatives (e.g., replacing "progressive ways and liberal values" with "our group's values"). The goal of these items is to see what, if any, biases people might have in making attributions about authoritarianism to left- versus right-wing persons. To the degree that the de-politicized items are rated by participants as more *authoritarian*, it suggests the items are measuring authoritarianism – but people are biased to believe otherwise (as some research suggests they will be; Frimer et al., 2014).

Selection of Big 5 Inventory Items. For discriminant validity, we further selected the 9 pro-trait items from the highly-cited MINI Big 5 Inventory (Donnellan et al., 2006; see Appendix).

Task description. Participants were always presented with 19 (10 RWA/LWA + 9 Big 5) items in a random order and asked to make a judgment about each item. Participants in both RWA and LWA conditions always had the same discriminant validity comparison Big 5 items. Regardless of item type, for each item, participants judged on a 1-3 scale whether or not someone who responded "yes" to that item would, in fact, be an authoritarian.

Participant Ideology. All participants further completed the same standard two-item political conservatism scale used in Study 1. As in Study 1, in order to provide easy descriptive summaries, for Study 2 we converted this measurement to a dichotomous measure in a manner identical to prior research (n = 365 for those analyses, with 236 liberals and 129 conservatives).

Results and Discussion

As seen in Figure 1, results revealed clear evidence of discriminant validity for both LWA (standard and de-politicized) and RWA as an authoritarianism measurement. Paired-sampled t-tests comparing each authoritarianism questionnaire set's average to the average from the comparison group revealed strong and significant validity effects for LWA (t[135] = 19.13, p < .001, d = .36, LCI = .02, UCI = .70), De-Politicized LWA (t[140] = 20.28, p < .001, d = .36, LCI = .02, UCI = .69), and RWA (t[135] = 22.12, p < .001, d = .37, LCI = .03, UCI = .71). Looked at another way, one-sample t-tests revealed that all six tests significantly differed from the mid-point of the scale (2), with the

authoritarianism questionnaires skewing greater than the midpoint (*t*'s > 12.1, *p*'s < .001) and the Big 5 questions skewing below the midpoint (*t*'s < -.14.6 , *p*'s < .001). This suggests that the authoritarianism questions for all three scales are indeed measuring authoritarianism – as they lean heavily towards the "very likely authoritarian" end of the scale – while the Big 5 questions do not measure this construct.

These metrics overwhelmingly provide content validity support for the pro-trait items in Conway et al's (2018) LWA measurement. As can be seen from Figure 1, however, LWA did show slightly lower (though still high in absolute terms) discriminant validity than RWA. And indeed, a 2 (within subjects discriminant validity: authoritarianism versus Big 5) X 3 (between subjects authoritarianism scale type: LWA versus RWA versus De-Politicized LWA) mixed-model ANOVA revealed an interaction between Authoritarianism/Big 5 and Type of Authoritarianism scale. To better understand the nature of this interaction, we performed two additional, and more focused, 2X2 interactions: Whereas the scale type by discriminant validity interaction was significant when comparing LWA and RWA (scale type by discriminant validity interaction $F = 6.84$, $p = .009$), this same interaction was not significant for comparing De-Politicized LWA to RWA ($F < 0.69$, interaction $p = .406$). These additional analyses suggest that part of the reason why the LWA scale was lower in validity was a (small but real) general bias against believing liberals could be authoritarian (see Frimer et al., 2014). When language was removed from the LWA scale indicating to participants a liberal leaning of the scale, the LWA scale showed very similar discriminant validity to the RWA scale (see Figure 1).

Regardless of these small differences across scale types, the present results clearly provide direct evidence of the content validity of the LWA scale as a measurement of authoritarianism. It showed strong discriminant validity. Not only did participants rate it as substantially higher than a scale not designed to measure authoritarianism, but they also rated it as significantly leaning towards the end of the scale clearly indicating it is measuring authoritarianism in absolute terms.

Figure 1: Discriminant Validity of Authoritarianism Scales By Type of Scale

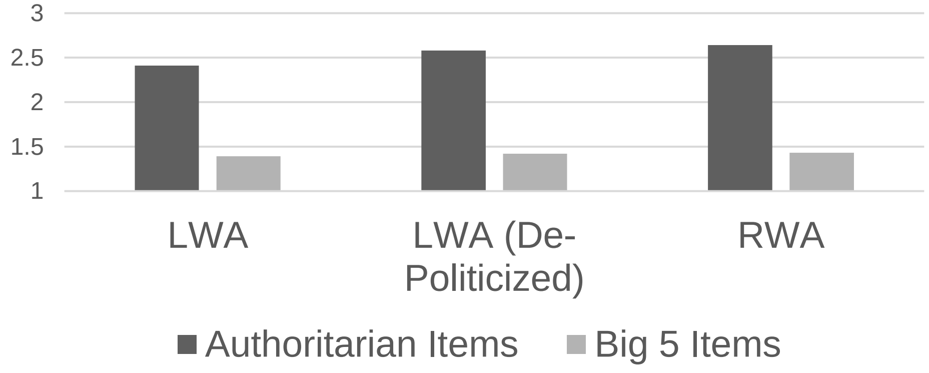

Expanded Transition to Studies 3-6: The Importance of Threat Perceptions to Authoritarianism Theories

Almost all prominent theories of authoritarianism maintain that it is psychologically linked to perceptions of threat or danger (e.g., Altemeyer, 1998; Duckitt et al., 2010; Feldman, 2003; Jost et al., 2003; Peterson & Gerstein, 2005; see Choma & Hanoch, 2017, for discussion). Indeed, it is “widely accepted” that authoritarianism and threat are empirically linked (Duckitt, 2013, p. 1).

However, essentially all of the work on the threat-authoritarianism relationship has focused on authoritarianism for *conservatives* (e.g., Altemeyer, 1998; Choma & Hanoch, 2017; Choma & Hodson, 2017; Jost et al., 2003; Ludeke et al., 2018; for discussions and evidence of the prevalence of RWA, see Chan et al., 2018; Conway et al., 2018; Malka et al., 2017). We would expect, if LWA is measuring authoritarianism, to find evidence that LWA is also related to measurements of threat perceptions.

Because ideology often overlaps with various outcome measurements, it can be difficult to disentangle the effects of ideology from the effects of other phenomena such as threat (e.g., Conway et al., 2018; Duckitt et al., 2010; Thomas, 2013). This problem exists equally for measurements of both RWA and LWA. In the present work, address this issue by adopting approaches with complementary strengths and weaknesses. We first compare LWA and RWA on identical scales that are completed by all participants on both general ecological threats (Study 3) and on the COVID pandemic more specifically (Study 4). Then, we randomly assign participants to threat-related measurements that use similar language, but manipulate whether the content of the language leans liberal or conservative (Study 5). We additionally asked participants how threatened they felt by a political opponent (Study 6). All studies (3-6) further control for self-reported ideology. As seen below, this triangulating approach provides evidence, across different ways of conceptualizing threat, that LWA is positively related to threat perceptions – above and beyond political ideology.

Study 3: Expanded Methods, Results, and Discussion

Methods

Study 3 was pre-registered on the *Open Science Framework* and can be accessed

at [*link to be inserted upon publication*]. All predictions were *a priori*. Study 3 was part of a larger study on rigid thinking and health. Several measurements from that study were relevant to our current investigation: Participants from all regions of the United States completed either an RWA or LWA scale, measurements of ecological stress tied to the area in which they lived, a two-item political ideology measure, and a personal income measurement. We expected small effect sizes for both RWA and LWA, and thus we employed a large sample size.

Participants

A sample of 4,988 U.S. adults were recruited using Amazon's *Mechanical Turk (MTurk)*. The sample was 57% female and was slightly left-leaning politically (4.2 on a political conservatism scale with 4.5 as the midpoint). One-hundred thirty three participants failed an attention check question, leaving 4,855 for analysis.

Importantly for the present analysis, participants resided in all regions of the United States, including participants from all 50 states. No one region dominated, and percentages from each state reflected the population distribution from the nation as a whole: The largest percentages of participants (by state) resided in California (11%) Florida (9%), Texas (8%), New York (6%), Pennsylvania (5%), Ohio (4%), North Carolina (4%), Michigan (4%), Illinois (4%), and Georgia (4%).

Measures

LWA/RWA. Participants were randomly assigned to receive either the 20-item LWA scale (Conway et al., 2018) or the parallel 20-item RWA scale (Altemeyer, 1996).

Ecological Stress Measurements. Participants were asked a series of questions related to the likelihood of prevalence of various ecological threats in the area in which

they live. These threats were drawn from prior work on the effect of ecological stress on the emergence of cultural beliefs related to authoritarianism and freedom (e.g., Beall et al., 2016; Conway et al., 2014; Conway et al., 2017; Fincher & Thornhill, 2012; Kitayama et al., 2006, 2010; Murray & Schaller, 2010; Oishi et al., 2017; Van de Vliert, 2013; Van de Vliert & Conway, 2019). These included a question each for *pathogen prevalence, natural disaster prevalence, harsh climate prevalence,* and *mountain (i.e., frontier topography) prevalence.* Specifically, items (all anchored on a 1-7 scale) were:

I feel the primary area where I live has a lot of disease.

I feel the primary area where I live has a lot of natural disasters.

I feel the primary area where I live has a harsh climate.

I feel the primary area where I live has a lot of mountains.

Finally, we included one item that specifically identified stress resulting from dangers in the natural environment (also on a 1-7 scale):

I sometimes feel stressed about the dangers present in the natural environment where I live (including disease, harsh climate, natural disasters, and other forms of environmental stress).

These five items were all modestly correlated with each other and thus we further produced a summary *Ecological Stress* score (conceptually similar to Conway et al., 2017) by converting each item to a *z*-score and averaging (standardized *alpha* = .72).[4]

Co-Variates: Political Ideology and Income. All participants further completed

[4] Some research suggests that conservatives care more about physical threat – and especially disease/disgust based threat (e.g., Terrizzi et al., 2013) – than liberals do (see Crawford, 2017, for a review). Thus, there is reason to suspect that such threats are not ideologically neutral. And indeed, in a preliminary study (n = 467) showing results identical to the pre-registered Study 3 presented here, we found that these ecological threats lean *slightly* towards conservatives. That was also true in the present study. However, this would actually work against LWA showing a relationship with threat in the present study – and part of the reason why we controlled for ideology directly.

the same standard two-item political conservatism scale used in Studies 1 and 2. Replicating past work (Conway et al., 2018), RWA was positively correlated with political conservatism ($r = .68$, $p <.001$), while LWA was positively correlated with political liberalism (reverse-scored conservatism scale; $r = .58$, $p < .001$). Further, participants also completed a standard measurement of personal income.

Analytic Strategy

For Study 3, we performed both zero-order and hierarchical regressions for each ecological stressor predicting LWA and RWA. Hierarchical regressions included political ideology and income at Block 1, and then each ecological stress measurement (separately) at Block 2. Thus, inferential tests reported for hierarchical regressions at Block 2 are for the added predictive ability of each ecological stressor, beyond ideology and income.[5]

Results and Discussion

Table 3 presents results from Study 3. As can be seen there, zero-order *betas* generally revealed that ecological stress measurements predicted both LWA and RWA. The overall Ecological Stress summary measurement revealed significant (p's < .001) effects of ecological stress as a predictor, with almost equal effect sizes for LWA and RWA. Looking at the ecological stress measurements separately told a similar story: In

[5] We did not perform analyses with all ecological stressors simultaneously entered at Block 2 because that was not relevant to our current theoretical purpose – and indeed would possibly interfere with it. Entering all ecological stressors at Block 2 would be relevant to questions concerning the unique predictive ability of each ecological stressor above and beyond the other ecological stressors. However, that is beyond our present purpose, which was to test the overall relationship of ecological stress with LWA and RWA. Entering all ecological stressors at Block 2 would in fact unnecessarily remove variance that was important to our purpose (see, e.g., Breiman & Freedman, 1983, on the dangers of including too many predictors). Thus, all analyses on individual ecological stressors did not include the other stressors.

all cases except for mountain prevalence predicting LWA, each ecological stress measure significantly predicted both LWA and RWA.

Importantly, subsequent hierarchical regressions entering the summary Ecological Stress measurement at Block 2 revealed Ecological Stress still significantly predicted both LWA and RWA when first entering political conservatism and income at Block 1 (see right half of Table 3), and analyses of individual ecological stress measurements revealed a similar story.[6]

Study 3 revealed clear threat-based predictors of authoritarianism on the right *and* on the left: Perceptions that ecological stressors were prevalent in their geographic locale – as well as a subjective measurement of danger from those stressors – were significantly positively predictive of both RWA *and* LWA.

Study 4: Expanded Methods, Results, and Discussion

Method

Participants. As a part of a larger project within the USA on COVID perceptions, Amazon's *Mechanical Turk* (*MTurk*) participants completed nearly-identical batteries of items in Study 4a (N = 297), Study 4b (N = 285), and Study 4c (N = 502) from March 30 to April 3 (2020).[7] Participants were randomly assigned to complete either the LWA or RWA measurements from Study 3.[8] Further, all participants completed measurements

[6] Some analyses from the sample for Study 3 were reported in Conway, Houck, et al., 2021. However, none of those analyses overlap with the present study, so all Study 3 analyses are entirely novel. Further, the Study 3 sample also included multiple measures related to physical health or health-related activities. These additional measures are not relevant to the present purpose.
[7] Given recently-identified potential quality issues with *MTurk* (e.g., Kennedy et al., 2018), we ensured the highest quality of data by including several screener questions that participants had to answer correctly to be included in the study. Evidence suggests that *MTurk* still produces excellent data given such safeguards (Kennedy et al., 2018).
[8] All three studies contained a paragraph-writing prime at the beginning of the study: Participants wrote about COVID-19, a happy topic, a neutral topic, or no topic. However, all the relationships reported here were essentially identical in each priming condition. Further, we found no consistent main effects of the prime on anxiety, self-reported ideology, or on voting preferences. Thus, the prime is irrelevant to the key storylines reported here, and we mention it no further.

Table III

Study 3: Individual-level Judgements of Ecological Stress Predicting Left- and Right-Wing Authoritarianism

	Zero-Order		Block 2 (Block 1 = Income and Conservatism)	
	**LWA**	**RWA**	**LWA**	**RWA**
**Ecological Stress Measure**				
Pathogen Prevalence	*.09****	*.14****	*.12****	*.10****
Natural Disaster Prevalence	*.05**	*.12****	*.10****	*.08****
Harsh Climate Prevalence	*.09****	*.06***	*.10****	*.03^*
Mountain Topography Prevalence	*.03^*	*.05**	*.07****	*.00*
Subjective Ecological Stress	*.16****	*.08****	*.13****	*.08****
**TOTAL ECOLOGICAL STRESS**	**.12*** **	**.13*** **	**.15*** **	**.09*** **

Notes: Total *N* = 4855. LWA *N* = 2470; RWA *N* = 2385. All metrics = standardized *betas*. Total ecological stress = summary measure (see text). ***p* <= .001; ***p* <= .01;**p* <= .05, ^*p* <= .10.

related to their perceptions of COVID-19: Perceived threat of COVID-19 and their reaction to government responses. Because the three samples were nearly identical and showed the same pattern, for the sake of brevity we combine them in a pooled analysis.

Coronavirus Threat and Government Response Questionnaires. Participants in Study 4 completed six items concerning how threatened or worried they were about COVID-19, for example: “Thinking about the coronavirus (COVID-19) makes me feel threatened” (standardized *alpha* = .88).

Participants in Study 4 also completed multiple items concerning their political beliefs about their government's response to the crisis. We focus here on two cross-governmental dimensions most relevant to participants' feelings of threat related to COVID-19 (all scale *alphas* > .86): The degree they wanted the government to restrict citizens to help stop the spread of the virus (*Restriction*; for example, "I support [Federal/State/City] government measures to restrict the movement of American citizens to curb the spread of Coronavirus (COVID-19)"), and the degree that participants wanted their governments to punish citizens who violated social distancing rules (*Punishment;* for example, "I want my [Federal/State/City] government to severely punish those who violate orders to stay home"). For each belief dimension, participants completed six questions (two for each level of government), and we aggregated the six items for each dimension to create scores for *Restriction* and *Punishment*.

Control Variables. To measure self-identification with political conservatism, participants completed the same *Political Conservatism* scale from Study 3 (*alpha* = .95). All participants in Study 4 additionally completed measurements of age, biological sex assigned at birth, and the population size of the city in which they resided. Further, in Study 4c only, participants completed measurements of income and education level (see Online Supplement).

Study 4 Results and Discussion

Analyses for Study 4 as a whole are reported in Table 4 below, and disaggregated analyses for each sample separately are presented in the supplement accompanying this paper. As can be seen in the supplement, the pattern of results was consistent across samples.

Table IV

Study 4: COVID Threat, Desired Restriction, and Desired Punishment Predicting Left- and Right-Wing Authoritarianism

	Zero-Order		Block 2 (Block 1 = Demographics and Conservatism)	
	**LWA**	**RWA**	**LWA**	**RWA**
Perceived COVID Threat	***.25****	***-.28****	***.15***	***-.05***
Desired Restriction	***.24****	***-.21****	***.16***	***-.05***
Desired Punishment	***.23****	***.02***	***.23****	***.22****

Notes: All metrics = standardized *betas*. ***p <= .001; **p <= .01;*p <= .05, ^p <= .15.

Zero-order standardized *betas* revealed that LWA was significantly positively related to Perceived COVID Threat (*beta* = .25, $p < .001$), Desire for Government Restriction (*beta* = .24, $p < .001$), and Desire for Government Punishment (*beta* = .23, $p < .001$). Subsequent hierarchical regressions revealed LWA's relationship to all three variables remained significant at Block 2 when entering political ideology, age, sex, and resident city population at Block 1 (see Table 4).

RWA also showed a theoretically consistent pattern: As expected by the ideologically-loaded nature of COVID-related issues (see Conway et al., 2020), RWA showed generally the opposite pattern of LWA at zero-order. However, when controlling for ideology and demographic variables at Block 1, at Block 2 these relationships altered such that there was no relationship between RWA and COVID Threat or Restriction. Further, the RWA-Punishment relationship became significantly positive (indeed, with a

similar effect size as the LWA relationship), suggesting that isolating the authoritarian part of right-wing authoritarianism yielded more desire for punishment (even on an issue that leans liberal).

Taken together, Study 4 corroborates and extends Study 3. Whereas Study 3 focused on sensitivity to ecological stressors at a very general level, Study 4 focused on threat perceptions and desired responses to a very specific stressor. Across both studies, threat sensitivity (and related measures) significantly predicted LWA.

This is not merely an artifact of the overlap of political ideology with COVID threat perceptions. As in Study 3, in Study 4 the threat-LWA relationship held when controlling for self-reported political ideology. Thus, there is something *beyond* ideology that accounts for the pattern in these studies. Consistent with the large literature on authoritarianism and threat for RWA and growing evidence for a similar relationship for LWA (Conway et al., 2019; Manson, 2020), the most likely interpretation is that it is the *authoritarianism* part of LWA that accounts for the relationship.

Study 5: Expanded Methods, Results, and Discussion

Study 3 showed evidence that, on the exact same (and largely ideologically-neutral) measures of threat, high LWA and RWA persons report similar levels of threat sensitivity above and beyond political ideology. Study 4 showed that LWA predicts perceived COVID threat above and beyond political ideology (and generally did so more than RWA, which is understandable given the potential ideological conflict high-RWA persons might have over the disease). Study 5 employed a different method of evaluating threat with complementary strengths and weaknesses. Specifically, for Study 5, we created separate, ideologically-*balanced* scales that attempt to tap into threat in a parallel

fashion for conservatives and liberals (for an example with dogmatism, see Conway et al., 2016). In Study 5, we use this method by creating two different, ideologically-balanced scales of the threat-related construct *Belief in a Dangerous World* (*BDW*; Altemeyer, 1998).

There is a long history of associating right-wing authoritarianism with *Belief in a Dangerous World* (BDW) and, indeed, much of the evidence for the threat-authoritarianism relationship is based on *BDW* (e.g., Altemeyer, 1998; Crawford, 2017; Duckitt, 2001; Duckitt et al., 2002; Duckitt et al., 2010; Jost et al., 2003). However, many of the beliefs outlined on the well-used *Belief in a Dangerous World* scale are not ideologically neutral, and this ideological content makes a direct comparison with LWA scientifically challenging. As a result, we created a new BDW scale that, while keeping the same language about danger, pointed the participant towards ideological concerns of more import to liberals. We then followed the approach prior researchers have applied with other constructs (Conway et al., 2016; Conway et al., 2018; Conway et al., 2020) and used both the original *BDW* (focused on conservative threats) and the new *BDW* (focused on liberal threats) to predict both RWA and LWA. In all cases, we controlled for political ideology.

Method

Study 5 was pre-registered on the *Open Science Framework* and can be accessed at [*link to be inserted upon publication*]. All predictions were *a priori*.

Participants. Four hundred and twenty-one U.S. adults were recruited using Amazon's *Mechanical Turk (MTurk)*. Study 5's sample had a mean age of 38, largely identified as Caucasian (82%), held a fairly even split between males and females (49%

female), and was slightly left-leaning politically (4.3 on a political conservatism scale with 4.5 as the midpoint).

LWA/RWA and Ideology. Participants were randomly assigned to receive either the RWA scale or the LWA scale. All participants also completed the same standard two-item political conservatism scale as in Studies 1-4.

Belief in a Dangerous World: Conservative Version (BDWC). Participants were further randomly assigned to either the conservative or liberal Belief in a Dangerous World scale.

In the original conservative scale, some of the items emphasize ideological content more harmonious with a conservative ideological focus, such as the destruction of the world by God or the preponderance of crime. The presence of these items may further influence the context of other items that appear to be more ideologically neutral.

Belief in a Dangerous World: Liberal Version (BDWL). Half of the participants completed a modified version of the *BDW* scale designed to focus on threats in domains more harmonious with the ideological focus of liberals: Environmental concerns, lack of medical care, and fighting wars. This modified *Belief in a Dangerous World Liberal* scale inserted a new content domain for seven of the twelve items, such that it intentionally pointed the potential danger in the item to liberal content domains, while keeping the danger-related language the same.

An example will illustrate. A (reverse-scored) item from the original conservative Belief in a Dangerous World scale reads: “The ‘end’ is not near. People who think that earthquakes, wars and famines mean God might be about to destroy the world are being foolish.” This item in the modified *Belief in a Dangerous World Liberal* scale was

adapted to read "The 'end' is not near. People who think that increases in destructive natural phenomena mean global warming might be about to destroy the world are being foolish."

In this way, seven of the twelve items were adapted to direct the item towards liberal-focused content. However, the key language of the items that illustrated the belief that the world is dangerous (e.g., words that portray worsening conditions and an inherent susceptibility to danger) were left identical (or as identical as the context allowed) in all cases.

Consistent with Conway et al.'s (2016, Study 1) work on dogmatism, for the modified *BDWL* scale, we kept five of the items in their original state. These items on the surface appeared more ideologically neutral (e.g., "If a person takes a few sensible precautions, nothing bad will happen to him or her. We do not live in a dangerous world") and yet for participants receiving the *BDWL*, these items were completed in the context of a scale clearly about liberal-leaning ideological threats.

The resulting *BDWL* scale is as follows (bold words are those altered or inserted from the original scale):

It seems that every year there are fewer and fewer truly respectable people ***[who care about the environment]****, and more and more persons with no morals at all who threaten everyone else.*

Although it may appear that things are constantly getting more ***[environmentally]*** *dangerous and chaotic, it really isn't so. Every era has its problems, and a person's chances of living a safe, untroubled life are better today than ever before.*

If our society keeps degenerating the way it has been lately, it's liable to collapse like a rotten log and everything will be in chaos.

Our society is <u>not</u> *full of immoral and degenerate people who prey on decent people. News reports of such cases are grossly exaggerating and misleading.*

The "end" is <u>not</u> *near. People who think that increases in* ***[destructive natural phenomenon mean global warming]*** *might be about to destroy the world are being foolish.*

There are many dangerous people in our society who will attack someone out of pure meanness, for no reason at all.

Despite what one hears about ***[a lack of medical care]****, there probably is just as much* ***[good health care]*** *now than there ever has been.*

Any day now, ***[environmental chaos and social]*** *anarchy could erupt around us. All the signs are pointing to it.*

If a person takes a few sensible precautions, nothing bad will happen to him or her. We do not live in a dangerous world.

Everyday, as our society becomes more ***[immoral]****, a person's chances of* ***[dying due to unlawful wars or lack of proper medical care]*** *go up and up.*

Things are getting so bad, even a decent ***[person who respects their environment]*** *can still become a victim of the senseless destruction of others.*

Our country is not falling apart or rotting from within.

Inter-item reliability for the scale was satisfactory in both conditions (Belief in a Dangerous World Conservative *alpha* = .87; Belief in a Dangerous World Liberal *alpha* = .81).

Results and Discussion

Table 5 presents results from Study 5. As can be seen there, results suggest that both LWA and RWA are related to Belief in a Dangerous World, but that the nature of those relationships is danger-domain specific. Consistent with pre-registered predictions, zero-order relationships revealed that while RWA was significantly positively related to the standard BDWC scale that focused on threat content domains of more relevance to conservatives (*beta*[98] = .63, *p* < .001, *LCI* = .49, *UCI* = .74), the LWA scale was significantly positively related to the BDWL that contained threat domains of more relevance to liberals (*beta*[90] = .38, *p* < .001, *LCI* = .19, *UCI* = .54). Unexpectedly, LWA was also significantly negatively correlated with the conservative domain BDWC (see Table 5).

<u>Table V</u>

Study 5: Relationship Between Authoritarianism and Belief in a Dangerous World

	Zero-Order		Block 2 (Block 1 = Conservatism)	
	**LWA**	**RWA**	**LWA**	**RWA**
**Dangerous World Measure**				
Conservative Domains (Standard)	***-.34****	***.63****	***-.14***	***.53****
Liberal Domains (Modified)	***.38****	***.12***	***.42****	***.22****

Notes: N = 417. **p <= .001; **p <= .01;*p <= .05. All metrics = standardized *betas*.

Table 5 also indicates that these relationships go beyond mere political ideology, thus representing something specific to authoritarianism: The key relationships between LWA-BDWL (*beta* = .42, $p < .001$) and RWA-BDWC (*beta* = .53, $p < .001$) remained significant when controlling directly for political ideology – indeed, the relationships between RWA/LWA and their respective counterpart BDW scale became descriptively more similar to each other when controlling for ideology. Further, the non-matching ideological correlations both shifted more positively, such that the LWA-BDWC correlation became non-significant, while the RWA-BDWL correlation became significantly positive (though still much lower descriptively than the RWA-BDWR correlation as expected).

These results suggest that while authoritarians on both sides of the political spectrum show heightened sensitivity to threat, the kind of threat that is important differs for LWA and RWA. This effect is more than *simply* political ideology, but rather says something about authoritarianism specifically: The key effects hold when controlling for self-reported political ideology. By balancing the ideological content via scales with both liberal and conservative content and controlling for self-reported ideology, this offers

additional support for the fact that authoritarianism on both the right *and the left* is related to threat concerns. Recall that the majority of the language – and all danger-related language – on the *BDWL* was identical to the language on the *BDWC*.

It is important to remember that the relationship between Belief in a Dangerous World (Conservative Version) and RWA has been previously used by scientists to infer a threat-authoritarianism relationship on the right side of the political spectrum (e.g., Altemeyer, 1998; Crawford, 2017; Duckitt, 2001; Duckitt et al., 2002; Duckitt et al., 2010; Jost et al., 2003). Because we must use the same standard of evidence on both sides (Tetlock, 1994), it is similarly reasonable to interpret an *LWA-BDWL* relationship as evidence for a threat-authoritarianism relationship on the left side of the political spectrum.

These results also reveal that this effect is stronger and more pervasive for authoritarianism on the right: The RWA effects were stronger overall, and RWA was a significant *positive* predictor of the liberal-content focused BDW scale when ideology was controlled for. Thus, while offering no support for an account that suggests danger concerns do not matter in producing authoritarianism on the left, they do suggest the possibility of asymmetric influence. We return to this issue below in the general discussion.

Study 6: Expanded Methods, Results, and Discussion

Prior research suggests that, above and beyond political ideology, LWA uniquely predicts support of liberal candidates in two elections that were viewed as especially threatening, but did not do so in an election that was less threatening (Conway & McFarland, 2019; Conway et al., 2020). However, while the authors of that work

speculated that, consistent with models of authoritarianism, perceived threat from the candidate in power (e.g., Donald Trump) is the likely mechanism by which LWA operates, no research to date has directly reported measurements of threat from the government in power (see Conway & McFarland, 2019; Conway et al., 2020). Study 6 used data from two samples collected five days apart in December 2019. In both samples, we evaluated the degree that LWA uniquely predicts perceptions that the then-sitting U.S. president was threatening, and further tested an LWA→Threat→voting intentions path (note that the predictive voting power of authoritarian measurements was recently used as validity evidence for conservative authoritarianism; Nilsson & Jost, 2020). Because both samples used nearly-identical measures and showed the same basic pattern, for the sake of brevity we consider them together.

Method

Participants. Six hundred and fifty U.S. adults were recruited using Amazon's *Mechanical Turk (MTurk)* as part of a larger project studying political beliefs. Of those, 533 completed all measurements relevant to Study 6, thus comprising our final sample.

***LWA/RWA and Ideology*.** Participants were randomly assigned to receive either the RWA scale or the LWA scale. All participants also completed the same standard two-item political conservatism scale used in prior studies, as well as measurements of biological sex assigned at birth and age.

***Trump Threat Perception*.** Participants also completed two items of threat-based concerns about the U.S. presidential administration (*alpha* = .94): "When I think of Donald Trump, it makes me feel a sense of threat," and "When I think of Donald Trump, it makes me feel anxious for my country's future."

Voting Intent. Participants completed single-item measures of their intent to vote for the Democratic nominee (at that time, yet to be determined) in the upcoming 2020 election, and their intent to vote for Donald Trump in the upcoming 2020 election (we reverse-scored this item as Opposition to Trump).

Results and Discussion

Consistent with expectations, LWA was significantly positively related to Perceived Trump Threat at Block 2 (controlling for political ideology, biological sex assigned at birth, and age), *beta* [256] = .18, $p < .001$, whereas RWA was negatively related to Trump Threat at Block 2, *beta* [277] = -.19, $p < .001$.

Further, as visually illustrated in Figure 2, evidence revealed strong support (while controlling for political ideology) for an LWA→Perceived Trump Threat→Democratic Candidate Support Path, indirect effect = .07 (LCI = .02, UCI = .13), $p = .004$. Similarly, evidence revealed strong support for an LWA→Perceived Trump Threat→Oppose Trump Path, indirect effect = .08 (LCI = .04, UCI = .14), $p = .003$.

These results provide additional corroboration of the evidence linking LWA to threat. As before, they cannot be accounted for by political ideology: It is not merely that liberals felt Trump was more threatening (although liberals overwhelmingly did), but rather that *authoritarian* liberals were uniquely prone to believe Trump was threatening. Taken together with the evidence so far, this suggests wide grounds for believing that the LWA scale captures threat-sensitivity that is more than *mere* liberal ideology.

Importantly, Study 6 further demonstrates the practical utility of considering LWA as a construct in helping us better understand voting intent for the 2020 election.

Specifically, high LWA persons' heightened sensitivity to threats from Trump accounts for part of why they were especially likely to vote for the democratic party (again, controlling for ideology and demographic variables).

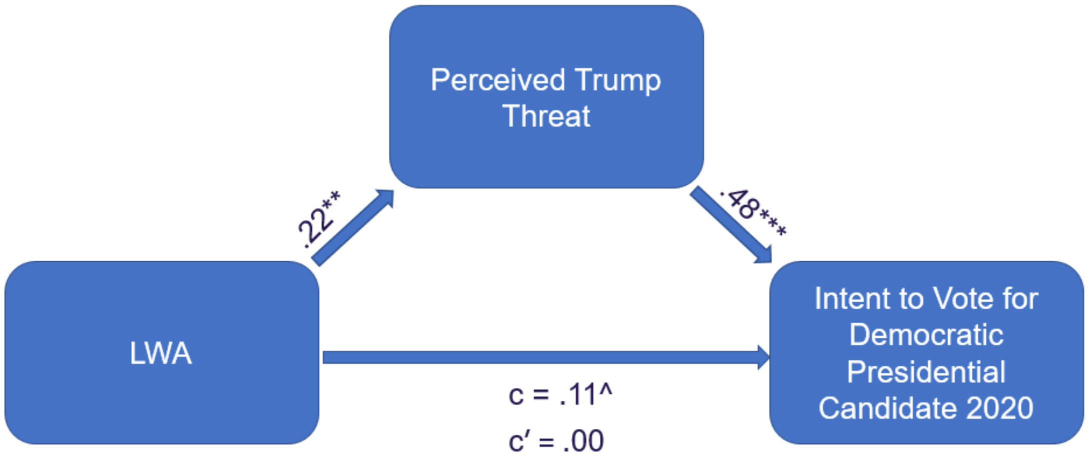

Figure II. Study 6: LWA→Perceived Trump Threat→Voting Intent

Study 7: Expanded Methods, Results, and Discussion

Restrictive norms are central to conceptualizations of authoritarianism (e.g., Altemeyer, 1996; Duckitt et al., 2010; Feldman, 2003). Thus, we would expect that LWA would uniquely predict support for left-leaning norms that focused on restriction. Study 7 tested one such norm: Support for restrictive language norms.

Method

Participants. A sample of three hundred and fifty U.S. adults were recruited using Amazon's *Mechanical Turk (MTurk)*.

LWA/RWA, Ideology, and Demographic Items. Participants were randomly assigned to receive either the RWA scale or the LWA scale. All participants also

completed the same standard two-item political conservatism scale as in Studies 3-6, as well as measurements of age and biological sex assigned at birth.

Restrictive Norms Support. Participants completed four items concerning their support for restrictive communication norms (items and introduction were adapted directly from Conway et al's 2017 restrictive PC norms condition). The items were preceded by the following instructions:

> *Now, we would like to get your opinions on societal norms. In our modern society, we have norms that dictate that we refrain from saying negative things – especially those things deemed as politically incorrect to say. These norms state that it is better to have rules that constrain us from anything that might sound too-negative or might be offensive to members of particular groups. We want to get your opinion on these norms.*

Participants then completed the following four items: "I am in favor of norms (such as "Political Correctness" norms) that restrict what people in society can say about others, especially other groups," "I think norms restricting the negativity of communication have value," "I strongly oppose societal pressures to restrict what people are 'allowed' to say without retribution" (reverse-scored), and "In general, I believe strongly in the value of societal norms governing communication." Inter-item reliability for the scale was satisfactory (*alpha* = .85).

Results and Discussion

Consistent with expectations, LWA was significantly positively related to Restrictive Norm endorsement (standardized *beta*[171] = .35, $p < .001$), and this relationship remained significant even when accounting for political ideology, age, and

biological sex assigned at birth at Block 2 (*beta* = .19 , *p* = .027). RWA was not significantly related to PC Norm endorsement at either Block 1 or Block 2. These results clearly offer additional support for our present purpose. Specifically, Study 7 shows that, above and beyond political ideology, LWA is predictive of a desire for society to have and to enforce restrictive communication norms.[9]

Study 8: Expanded Methods, Results, and Discussion

Stereotypes and prejudice are typically associated with conservatives in general, and right-wing authoritarianism in particular (e.g., Jost et al., 2003). More recent research has suggested, however, that prejudice can occur on both sides of the political spectrum. For example, work has revealed that liberals show negative attitudes towards African Americans when they believe they possess conservative attitudes such as religious fundamentalism (Chambers et al., 2013). Paralleling work on the unique contribution of right-wing authoritarianism to prejudice, newer work has tied *left*-wing authoritarianism to group attitudes by revealing that persons high in LWA are more likely to exhibit the equivalent of *modern racism* on a scale that targets Christian fundamentalists (Conway et al., 2018).

This prior work on LWA has been criticized for having “selected targets of prejudice that are rarely victims of prejudice in the US” (Saunders et al., 2020). To fill in this gap, Studies 8 and 9 apply the LWA framework to two groups that have historically

---

[9] Studies 6 and 7 also contained a manipulation of thinking about Trump: Half of the participants were assigned to write a brief paragraph about Trump at the beginning of the study, the other half to write about broccoli. This manipulation is not relevant to the purpose of the studies outlined here, and analyses within-condition across studies reveal essentially identical effect sizes for the key tests in both conditions. Thus we discuss this no further. Additionally, both Studies 6 and 7 contained other measurements not relevant to the primary storyline here – see supplemental materials.

been the target of prejudice: Religious African American persons and Jewish persons who support Israel.

In the current study, we draw on prior work suggesting that the presence of perceived conservative attitudes will cause liberals to show dislike of African Americans. One such attitude is religiosity (Chambers et al., 2013). This is important because, in the modern U.S., the large majority of African Americans are religious (for example, 77% of African Americans believe that “the Bible is the Word of God”; Diamant, 2018). Thus, if LWA uniquely predicts negative attitudes towards religious African Americans beyond political ideology, this suggests its potential power in understanding prejudicial attitudes towards a large group of persons that have historically been the targets of prejudice in the U.S. Extrapolating from survey data, a cautious estimate of the number of African Americans who “believe in the Bible” is 30 million persons.

Similarly, Jews in the U.S. have historically been the targets of prejudice. The majority of modern Jewish Americans support the state of Israel, with estimates as high as 90% (Newport, 2019). Thus, if persons high in LWA show negative attitudes towards this group beyond political ideology, this suggests the unique contribution of LWA to potential prejudice on a large group of persons that have historically been the targets of prejudice. Extrapolating from survey data, a cautious estimate of the number of Jewish Americans who support Israel is 4 million persons.

Method

Participants. Two hundred and seventy-one U.S. adults were recruited using Amazon’s *Mechanical Turk (MTurk)*.

LWA/RWA, Ideology, and Demographic Items. Participants were randomly

assigned to receive either the RWA scale or the LWA scale. All participants also completed the same standard two-item political conservatism scale as in Studies 3-7, as well as measurements of age, biological sex assigned at birth, and population of resident city.

Personal Group Favorability Ratings. All participants completed standard "feeling thermometer" measurements drawn from prior research (e.g., Dyrbye et al., 2019; Schaller et al., 2002) concerning their own personal views of particular groups. The directions preceding these ratings were as follows:

Please indicate below your responses to each group, where 1 = your personal view is VERY UNFAVORABLE towards this group, 5 = your personal view is neither favorable nor unfavorable towards this group, and 9 = your personal view is VERY FAVORABLE towards this group. In each of these items, you are indicating your own personal view, not what anyone else believes.

Participants were then presented with six groups and rated each group on a 1-9 feeling thermometer: *Bible-believing men, Bible-believing women, Bible-believing African-American men, Bible-believing African-American women, Strong supporters of the nation of Israel's interests who are also Jewish men, and Strong supporters of the nation of Israel's interests who are also Jewish women.*

Societal Attitudes. To distinguish their private views from their views of society (see Nosek, 2005; Schaller et al., 2002), all participants also completed responses to the same six target groups while considering, not their own views, but the views of society as a whole. This set was preceded by the following directions:

Please indicate below your responses to each group, where 1 = society in general

is VERY UNFAVORABLE towards this group, 5 = society in general is neither favorable nor unfavorable towards this group, and 9 = society in general is VERY FAVORABLE towards this group. In each of these items, you are NOT indicating your own personal view – rather, you are indicating what you think society in general believes.

The order with which participants completed the private versus societal measures was randomly assigned. Further, after each section (private or societal), participants were asked to indicate whether they had completed private or societal measures, and participants who answered incorrectly in either section were dropped.

Results and Discussion

As can be seen in Table VI, results overwhelmingly support the unique predictive validity of LWA for negative group attitudes towards African-Americans and Jews. When accounting at Block 2 for political ideology and the demographic factors, LWA predicted negative personal attitudes towards African-Americans who believed in the Bible and Jews who were supportive of Israel (all p's $< .001$). This occurred in spite of the fact that, as revealed in the bottom half of Table VI, there was, if anything, a positive relationship between LWA and participants' beliefs about societal views on these groups (although this relationship was generally non-significant once accounting for ideology and demographic factors).

As expected by theories of the ideology-prejudice relationship (e.g., Chambers et al., 2013), RWA was generally positively predictive of attitudes towards African-Americans who believe in the Bible. RWA was also positively predictive of favorable attitudes towards Jews who support Israel, although these relationships became non-

significant when accounting for ideology and demographic factors at Block 2.

Table VI

Study 8: LWA and RWA Predicting Attitudes Towards African Americans and Jews

	Zero-Order		Block 2 (Block 1 = Demographics and Conservatism)	
	**LWA**	**RWA**	**LWA**	**RWA**
**Personal Group Favorability:**				
Bible-believing men	*-.63****	*.62****	*-.47****	*.50****
Bible-believing women	*-.62****	*.58****	*-.51****	*.48****
Bible-believing African-American men	*-.58****	*.52****	*-.48****	*.37****
Bible-believing African-American women	*-.61****	*.55****	*-.51****	*.45****
Strong supporters of Israel/men	*-.53****	*.35****	*-.43****	*.13*
Strong supporters of Israel/women	*-.50****	*.30****	*-.39****	*.08*
**Societal Attitudes:**				
Bible-believing men	*.42****	*-.25***	*.35****	*-.12*
Bible-believing women	*.41****	*-.17**	*.38****	*-.00*
Bible-believing African-American men	*.24****	*.09*	*.22^*	*.05*
Bible-believing African-American women	*.21****	*.12*	*.18^*	*.17*
Strong supporters of Israel/men	*.28****	*-.09*	*.20^*	*-.11*
Strong supporters of Israel/women	*.29****	*.01*	*.26**	*.01*

Notes: Total N = 271. LWA N = 137; RWA N = 134. All metrics = standardized *betas*. ***p <= .001; **p <= .01;*p <= .05, ^p <= .10. Block 2 controls for political ideology, age, biological sex assigned at birth, and population of resident city.

Study 9: Expanded Methods, Results, and Discussion

Study 8 provided initial evidence that persons high in LWA are uniquely prone to negative attitudes towards persons in two groups historically the targets of prejudice: African-Americans and Jews. This LWA-negative attitude relationship occurs *if* certain characteristics of the groups – characteristics that describe the large majority of group members in each case (Diamant, 2018; Newport, 2019) – are made salient.

Nevertheless, it is possible that the methods of Study 8 affected the outcome by presenting the content issue first, both across items ("Bible-believing men" was the first item, thus making "bible-believing" and not ethnicity salient) and within-item ("Bible-believing" and "Supports Israel" were always first within-item, with ethnicity coming at the end). Study 9 directly accounted for this by providing conditions that, instead of first presenting the politically-relevant issue, presented the ethnicity first (e.g., "African-American men" and not "Bible-believing men" appears first in this condition).

As we will see below, this manipulation did not impact the results, thus effectively ruling out a language presentation bias issue from Study 8.

Method

Participants. one hundred and sixty-nine U.S. adults were recruited using Amazon's *Mechanical Turk (MTurk)*.

LWA/RWA, Ideology, and Demographic Items. As in Study 8, Participants were randomly assigned to receive either the RWA scale or the LWA scale. All other covariates and measures were also identical to Study 8.

Personal Group Favorability and Societal Ratings. Half of the participants were randomly assigned to complete standard "feeling thermometer" measurements that were identical to Study 8 (*Issue-First Condition*). The other half (*Ethnicity-First Condition*) completed measurements that were conceptually identical, but differed in two respects. (1) Instead of the opening questions being about "Bible-Believing Men" and "Bible-Believing Women," they were replaced with "African American Men" and "African American Women." (2) Language within-item was altered so that the ethnicity, and not the politically-relevant issue, came first. For example, "Bible-Believing African-

American Women" became "African-American Women who believe in the Bible."

This same principle was applied to the societal ratings – participants in the Issue-First Condition also received parallel issue-first societal ratings identical to Study 8, whereas participants in the Ethnicity-First Condition received altered questions that were the same as those described for personal favorability ratings (only with societal content). All other aspects of Study 9 were identical to Study 8.

Results and Discussion

All key results were essentially identical across both Ethnicity-First and Issue-First conditions.[10] As a result, for the sake of brevity, we collapse them here.

As can be seen in Table VII, results again overwhelmingly support the unique predictive validity of LWA for negative group attitudes. When accounting at Block 2 for political ideology and the demographic factors, LWA predicted negative personal attitudes towards African-Americans who believed in the Bible and Jews who were supportive of Israel in a manner descriptively and inferentially identical to Study 8 (all p's < .001).

Although there was more of a negative relationship between beliefs about societal attitudes and LWA than in Study 8, these results (in a manner identical to Study 8) generally became non-significant when controlling for ideology and demographics. Importantly for our purposes, both studies revealed a strong and consistent negative effect of LWA on private attitudes towards the two groups, with smaller (and generally

[10] There was a descriptive tendency for RWA to be more positively associated with African-American and Jewish personal favorability scores in the *Issue-First* condition than in the *Ethnicity-First* condition. This makes sense and suggests part of the drop in effect size for RWA for Study 11 might have been due to the addition of the Issue-First condition. However, the general tenor of the RWA results remains the same across these conditions, and as our paper is focused primarily on LWA, we did not pursue this potential RWA interaction any further. The larger point is that the language context manipulation has no bearing on the key relationship reported here between LWA and negative group attitudes.

non-significant) effects on beliefs about societal attitudes. In other words, these results clearly isolate this effect to the personal beliefs of our LWA participants – an effect that was consistently strong, significant, and negative – as opposed to their perceptions of what society at large believes.

As seen in Table VII, the results for RWA were in the same direction, but generally weaker (and at BLOCK 2, non-significant) than those for Study 8.

Taken together, Studies 8 and 9 demonstrate a consistent effect of LWA on personal attitudes towards two groups historically the targets of prejudice: African-Americans and Jews. Persons high in LWA are more likely to have negative attitudes towards members of those groups that are described in terms consistent with the beliefs of the majority of the groups' members. This effect occurs even when the ethnicity, and not the issue, is initially made salient in the ratings. And – of vital importance – it occurs even when controlling for ideology and demographic factors. As in Studies 3-7, the results in Studies 8 and 9 reveal that there is *something* about certain kinds of liberals that goes *beyond* mere ideology. Consistent with decades of research on the authoritarianism-prejudice link, that *something* is very likely best described as *authoritarianism*.

Table VII

Study 9: LWA and RWA Predicting Attitudes Towards African Americans and Jews

	Zero-Order		Block 2 (Block 1 = Demographics and Conservatism)	
	**LWA**	**RWA**	**LWA**	**RWA**
**Personal Group Favorability:**				
Bible-believing African-American men	*-.65****	*.26**	*-.59****	*.20*
Bible-believing African-American women	*-.64****	*.25**	*-.56****	*.20*
Strong supporters of Israel/men	*-.50****	*.19^*	*-.35***	*.09*
Strong supporters of Israel/women	*-.52****	*.22**	*-.40****	*.11*
**Societal Attitudes:**				
Bible-believing African-American men	*-.25**	*.27***	*-.21*	*.30^*
Bible-believing African-American women	*-.27**	*.29***	*-.21*	*.27^*
Strong supporters of Israel/men	*-.11*	*-.01*	*-.06*	*.08*
Strong supporters of Israel/women	*-.14*	*-.04*	*-.11*	*.08*

Notes: Total *N* = 169. LWA *N* = 80; RWA *N* = 89. All metrics = standardized *betas*. ****p* <= .001; ***p* <= .01;**p* <= .05, ^*p* <= .10. Block 2 controls for political ideology, age, biological sex assigned at birth, and population of resident city.

Study 10: Expanded Methods, Results, and Discussion

In their study on LWA, Conway and colleagues (2018) demonstrated that persons high in LWA showed higher levels of dogmatism, modern racism, and attitude strength in liberal-focused domains. However, these results have been criticized as potentially not representing anything beyond political ideology (Honeycutt & Jussim, 2020). To deal with this criticism, we here re-analyze the data provided by their LWA participants to control for political ideology. Further, up to this point, we have only controlled for ideology using broad self-reported ideological identification measurements

(liberal/conservative and democrat/republican). While this method has many strengths in providing unbiased ideology estimates (see Houck & Conway, 2019, for discussion), triangulation nonetheless suggests that we should also control for more specific political attitudes. To the degree that LWA effects still hold, this would reveal that liberal *authoritarian* attitudes are important in the effects above and beyond specific liberal content. Study 10 allowed for a very rigorous test of that by including, in a test of LWA's relationship to dogmatism, a covariate measurement of attitudes *on the domain of interest* with respect to dogmatism. As we will see, these results overwhelmingly suggest that it is authoritarianism, and not liberal content, that accounts for the LWA-Dogmatism relationship.

Method

Participants. For this re-analysis, we only focused on participants in the original two studies who completed the LWA questionnaire. As reported in Conway et al. (2018), for their Study 1, one hundred and seventy-eight undergraduates completed questionnaires for course credit. For their Study 2, one hundred and forty-seven participants were recruited from *MTurk*.

Measures. All participants completed the LWA scale. They further completed measurements of domain-specific dogmatism based on Rokeach's Dogmatism scale (adapted from Rokeach, 1960), an adapted Modern Racism scale that targeted religious minorities instead of ethnic minorities (adapted from McConahay, 1986), and a measurement of the strength of their attitudes about climate change (adapted from Conway et al., 2008; Conway et al., 2011).

Further, imbedded in the questionnaire was an item pertaining to the specific attitude domain that the dogmatism questionnaire is about (climate change): "How much do you agree with this statement?: Global warming is occurring and is human caused." Answers were given on a 1-9 scale where 1 = completely disagree and 9 = completely agree.

Results and Discussion

Conway et al. (2018) reported the zero-order correlations between LWA and dogmatism, modern racism, and attitude strength (all significantly positive). To establish the level of unique contribution above and beyond political ideology, here we use their publically-available data to control for political ideology. When accounting for political ideology, results revealed that significant variance remained for LWA predicting Dogmatism in their Study 1 (standardized *beta* = .29, $p < .001$) and Study 2 (standardized *beta* = .42, $p < .001$). Similarly, when accounting for political ideology, results revealed that significant variance remained for LWA predicting Modern Racism targeted at religious minorities in Study 1 (standardized *beta* = .53, $p < .001$) and Study 2 (standardized *beta* = .62, $p < .001$). However, although the relationships were still positive, the relationship between LWA and attitude strength became non-significant when accounting for political ideology in Study 1 (standardized *beta* = .09) and Study 2 (standardized *beta* = .06).

Adding attitudes about climate change as a predictor did not alter this pattern of results for LWA predicting Dogmatism (Study 1 *beta* = .22, $p = .014$; Study 2 *beta* = .49, $p < .001$) or Modern Racism (Study 1 *beta* = .59, $p < .001$; Study 2 *beta* = .61, $p < .001$). LWA predicting Attitude Strength remained non-significant but became less positive

(Study 1 *beta* = -.03; Study 2 *beta* = -.05). Importantly, the LWA-Dogmatism relationship remained significant even when directly accounting for an attitude that comprises a key part of the content of the dogmatism questionnaire.

Taken together, these results suggest that in the majority of Conway et al's original work – like all of the work presented here in Studies 3-9 – LWA predicts key phenomena above and beyond both general political ideological identification and a specific liberal attitude relevant to one of the key DVs.

Indeed, the latter finding is worth highlighting in regard to the dogmatism measure. It is noteworthy that controlling for climate change attitudes did not substantially alter the relationship between LWA and a dogmatism scale that specifically targeted environmental issues such as climate change. This suggests that the LWA-dogmatism relationship is not merely the result of attitudinal content overlap between the two scales – rather, it provides evidence that the scales are related because persons who score high on the left-wing authoritarianism scale are especially prone to dogmatism.

No construct will show unique predictive validity in every instance; but the overwhelming tenor of the combined work presented here reveals that LWA is a unique predictor of the kind of phenomena that conceptually an authoritarianism measure should predict.

Study 11: Expanded Methods, Results, and Discussion

Although we have controlled for political ideology throughout – and a domain-relevant ideological attitude in Study 10 – it is still possible that the effects of LWA would not be in evidence if we used more complex, content-based measures of ideology as a control. In Study 11, we use a scientifically-validated measure that captures more

specific ideological attitudes on two different dimensions: The Social and Economic Conservatism Scale (Everett, 2013). We focus on conceptually replicating the LWA-cognitive rigidity effects from Study 10 while controlling for this measure.

Method

Four-hundred and seventy-nine persons participated via Amazon's Mechanical Turk (Age *M* = 41.1, 53% Female). Participants completed Conway et al.'s (2018) measurement of LWA and two measurements directly relevant to cognitive rigidity: Altemeyer's (1996) Dogmatism measure and the short version of the Need for Closure Scale – Revised scale (Roets & Van Hiel, 2011). Finally, as a political ideology covariate, participants completed the 12-item Social and Economic Conservativism Scale (Everett, 2013), a scale which measures people's favorability towards conservative social and economic policies, respectively.[11]

Results and Discussion

Primary results again support the validity of LWA in predicting rigidity-based measures, even when using a more nuanced measure of ideology as a control. When controlling for both SECS ideology scales simultaneously, LWA was significantly positively predictive of both cognitive rigidity measures, predicting Need for Closure (*beta* = .23, $p < .001$) and Dogmatism (*beta* = .21, $p < .001$).[12]

---

[11] These data were drawn from Sample 1 of Costello et al. (2021). All analyses presented here are novel. We are grateful to Thomas Costello for sharing these data with us.

[12] Study 11 also contained personality measurements. On personality domains, when controlling for both ideology scales, LWA was negatively predictive of Openness (*beta* = -.21, $p < .001$), Altruism (*beta* = -.22, $p < .001$), Agreeableness (*beta* = -.19, $p < .001$), and Honesty/Humility (*beta* = -.22, $p < .001$). LWA was largely unrelated to Emotionality (*beta* = .07, *p* = ,197), Extraversion (*beta* = -.01, *p* =.873), and Conscientiousness (*beta* = -.07, *p* = .256). These exploratory relationships with personality variables generally (though not perfectly) conform to expectations for an authoritarian person and with past research on authoritarianism in both liberals and conservatives (see Costello et al., 2021, for a discussion).

Taken together, this set of results speaks directly to concerns that controlling for more nuanced measurements of ideology would make LWA invalid predictors of rigidity. Controlling for a scientifically-validated measurement of two-dimensional ideological attitudes (the SECS), LWA significantly predicted both measurements of cognitive rigidity in the correct direction. These results thus confirm that the prior results from Study 10 were not an artifact of the particular ideology measurements used in that study.

Parallel LWA/RWA and Within-Group Validity Tests: Expanded Discussion

Across nine studies using Conway et al's (2018) LWA measure, we have demonstrated the predictive power and theoretical relevance of LWA as a construct. In each case, we have controlled for participants' level of political ideology (as well as any available demographic factors that were measured in each study) using three different measurements of ideology. This set of results suggests there is *something* beyond mere ideology that explains high-LWA persons' increased sensitivity to threat (Studies 3-6), their support for restrictive communication norms (Study 7), their negative group attitudes (Studies 8 and 9), and their cognitive rigidity (Studies 10 and 11).

Of course, as noted in the introduction, measuring authoritarianism is tricky because, almost by definition, people are authoritarian *to a specific authority figure or group*. That means that all authoritarian measures contain content related to the specific group (e.g., the "right-wing" in right-wing authoritarianism) and to authoritarianism (e.g., the "authoritarianism" in right-wing authoritarianism; Conway, 2020). Left-wing

authoritarianism shares this measurement challenge with all other measurements of authoritarianism. Thus far, our primary method of overcoming this challenge has involved controlling directly for political ideological content/targets, thus isolating the "authoritarian" part of "[content domain] authoritarianism."

We here use available data to provide a big-picture summary evaluation of two of the additional methods: (1) Assigning participants to complete balanced LWA and RWA scales to test parallel authoritarianism content/targets simultaneously, and (2) performing within-ideological group analyses.

Comparable Parallel RWA and LWA Tests

Both in prior work (Conway & McFarland, 2019; Conway, Houck, et al., 2021) and in many of the studies reported here, participants were randomly assigned to complete either the LWA scale (Conway et al., 2018) or the parallel RWA scale (Altemeyer, 1996). Although for brevity we have not always highlighted comparative tests in the present paper, we use this data to provide a big-picture summary of available evidence concerning comparable LWA and RWA effects.

Our criteria for determining which effects are useful for comparison were as follows. (1) The independent variables have to be roughly parallel. (2) The dependent measures have to be roughly functionally equivalent. Perfect equivalence is not possible, because it is always partially uncertain if two scales mean the exact same things to high-LWA and high-RWA persons, or if adapted scales are functionally similar enough for comparison. However, this is a challenge for all research; and this challenge should not stop reasonable comparisons. Thus, we do not make claims of perfect equivalence – only

that the measures are defensibly equivalent enough that comparison is useful. Table VIII reports the specific samples and metrics used in the present summary.

More specifically, we included measurements that were both structurally similar (e.g., identical wording or only slight wording alterations) and psychologically similar (e.g, items that might be conceptually expected to produce the same valence for conservatives as for liberals). For example, our ecological stress items from Study 3 are identical across conditions, and it seems clear that these items ought to be psychologically associated with both LWA and RWA. Thus, this measure was considered a comparable measurement. However, we did not include the *restrictive norms* measurement from Study 7 because it focuses on norms liberals are especially likely to support, and we had no parallel measure of restrictive conservative norms in that study. Inclusion of that study would thus overly exaggerate the effects of LWA versus RWA.

There are potential non-equivalence issues with any measure, and our set of chosen measures is not perfect; however, we feel the resulting set provides a reasonable test of comparable measurements across LWA and RWA. The high correlation of LWA and RWA effects across measurements (described below) supports this contention.

Results are presented in Figure 3. Three things are noteworthy about this analysis evaluating comparable effects across RWA and LWA. First, almost all of the expected correlations are above zero (and the only one that is not above zero is for RWA). Second, the overall effect size for RWA (average r = .27) and LWA (average r = .28) are virtually identical. Third, and perhaps most tellingly, RWA and LWA effect sizes tend to be similar across comparable measurements. In fact, the correlation between their effect sizes reported in Figure 3 is r = .65. This suggests that when you use comparable

measures of authoritarianism (recall that Conway's LWA scale was designed to be parallel to Altemeyer's RWA scale) and comparable dependent measures, you get similar results for RWA and LWA.

Table VIII

Study 12: Metrics Used for LWA and RWA Summary Comparisons

**Conceptual DV**	**Study *n***	**Source**	**LWA Measure**	**RWA Measure**	**LWA DV**	**RWA DV**
Environmental Ecological Stress	4988	Present Paper, Study 3	Conway et al (2018) LWA	Altemeyer (1996) RWA	Summary Scale Reporting Ecological Stress in Local Environment	Summary Scale Reporting Ecological Stress in Local Environment
COVID-Related Threat Sensitivity	1084	Present Paper, Study 4	Conway et al (2018) LWA	Altemeyer (1996) RWA	Summary Scale Reporting Perceived COVID Threat	Summary Scale Reporting Perceived COVID Threat
Belief in a Dangerous World	421	Present Paper, Study 5	Conway et al (2018) LWA	Altemeyer (1996) RWA	BDW-Modified (adapted from Altemeyer)	BDW (Altemeyer)
Informational Mistrust of Opponents	340	Conway, Houck et al. (2021)	Conway et al (2018) LWA	Altemeyer (1996) RWA	Item concerning distrust of Republican Party	Item concerning distrust of Democratic Party
Reactance to Political Opponents	340	Conway, Houck et al. (2021)	Conway et al (2018) LWA	Altemeyer (1996) RWA	Item concerning emotional reactance to Republican Party	Item concerning emotional reactance to Democratic Party
Partisan Candidate Support	1582	Conway & McFarland (2019)	Conway et al (2018) LWA	Altemeyer (1996) RWA	Voting intention for Obama when Republicans held power	Voting intention for Trump when Democrats held power
Dogmatism	178	Present Paper, Study 10a	Conway et al (2018) LWA	Altemeyer (1996) RWA	Rokeach Dogmatism Scale (Environmental Issues)	Rokeach Dogmatism Scale (Religious Issues)
Dogmatism	147	Present Paper, Study 10b	Conway et al (2018) LWA	Altemeyer (1996) RWA	Rokeach Dogmatism Scale (modified to focus on Environmental Issues)	Rokeach Dogmatism Scale (modified to focus on religious issues)
Dogmatism	479	Present Paper, Study 11	Conway et al (2018) LWA	Altemeyer (1996) RWA	Altemeyer Dogmatism Scale	Altemeyer Dogmatism Scale
Attitude Strength	178	Present Paper, Study 10a	Conway et al (2018) LWA	Altemeyer (1996) RWA	Composite Measurement of Attitude Strength (environmental issue)	Composite Measurement of Attitude Strength (religious issue)
Attitude Strength	147	Present Paper, Study 10b	Conway et al (2018) LWA	Altemeyer (1996) RWA	Composite Measurement of Attitude Strength (environmental issue)	Composite Measurement of Attitude Strength (religious issue)
Modern Racism	178	Present Paper, Study 10a	Conway et al (2018) LWA	Altemeyer (1996) RWA	Modern Racism Scale (Religious Minorities)	Modern Racism Scale (Ethnic Minorities)
Modern Racism	147	Present Paper, Study 10b	Conway et al (2018) LWA	Altemeyer (1996) RWA	Modern Racism Scale (Religious Minorities)	Modern Racism Scale (Ethnic Minorities)
Need for Closure	479	Present Paper, Study 11	Conway et al (2018) LWA	Altemeyer (1996) RWA	Short Version Need for Closure Scale	Short Version Need for Closure Scale

Figure 3: LWA and RWA Effect Sizes on Comparable Dependent Measures

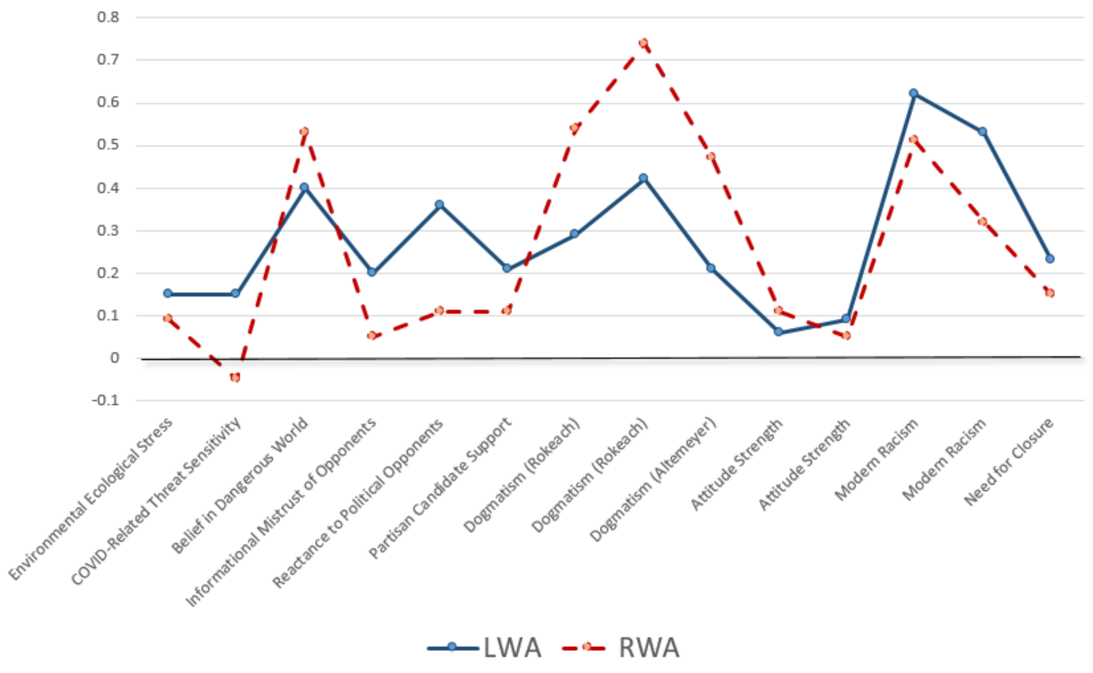

Within-Group Analyses: Comparing Liberals to Liberals

A further method for separating out ideology from authoritarianism that has complementary strengths and weaknesses is to perform within-ideological group analyses (see Wronski, 2018, for an example). In other words, one way to separate liberal authoritarians from liberal non-authoritarians is to look only at liberals. To accomplish this, for Studies 3-11, we performed further analyses that analyzed all key LWA effects by looking only at persons who scored on the liberal side of the 1-9 ideology scale (because the scale is anchored by 1 = liberal and 9 = conservative, for these analyses, we only included persons scoring below the midpoint). For Study 11, we included participants who scored below the midpoint on the scale for both the social and economic conservatism subscales. For ease of understanding, when necessary we reverse-scored

(and reverse-named; see Table note) variables so that positive numbers always meant the expected LWA effect. We also combined variables in some cases to make the results easier to digest (using the disaggregated variables yielded an identical set of results).

Results are presented in Table IX. As can be seen there, in the vast majority of cases, the within-group analyses in the last column corroborated whole-sample analyses in the preceding column (all whole-sample analyses in the table control for political ideology + any demographic variables measured in that study). As would be expected in any such large analyses, some significant whole-sample effects became non-significant within-group, and some non-significant whole-sample effects became significant within-group. However, almost all analyses of each type showed effects in the same direction, and the vast majority were significant in both kinds of analyses. This can be easily seen by the evaluating the average effect sizes across studies, which are very similar for both whole-sample (average *beta* = .30) and within-group (average *beta* = .35) analyses.

These analyses suggest that when we compare liberals to other liberals with different degrees of authoritarianism, we still (in the main) get the conceptually-expected relationships in this large array of studies. This provides additional triangulating evidence that the left-wing authoritarian is more of a reality than a myth.[13]

[13] We additionally re-computed the indirect path analyses from Study 6 within-group and found the exact same set of results reported on the whole sample. Specifically, looking only at liberals from Study 6 revealed support for an LWA→Perceived Trump Threat→Democratic Candidate Support Path, indirect effect = .08 (LCI = .02, UCI = .17), p = .014. Similarly, within-group evidence revealed support for an LWA→Perceived Trump Threat→Oppose Trump Path, indirect effect = .07 (LCI = .02, UCI = .15), p = .018.

Table IX

Studies 3-11: LWA Effects Both Whole-Sample and Within-Group (Liberals-Only)

	**Whole-Sample LWA**	**Liberals-Only LWA**
Perceived Ecological Threat (Study 3)	.15***	.10***
COVID Threat Sensitivity (Studies 4)	.15**	.09
Desired Restriction (Study 4)	.16**	.13*
Desired Punishment (Studies 4)	.23***	.09^
Belief in a Dangerous World (Study 5)	.42***	.64***
Trump Threat Sensitivity (Study 6)	.18***	.25**
Restrictive Communication Norms (Study 7)	.19***	.08
Negative Views/African Americans (Study 8)[a]	.50***	.54***
Negative Views/African Americans (Study 9)[a]	.58***	.67***
Negative Views/Jews (Study 8)[a]	.42***	.37***
Negative Views/Jews (Study 9)[a]	.39**	.47**
Dogmatism (Study 10a)	.29***	.39***
Dogmatism (Study 10b)	.42***	.40***
Modern Racism/Relig. Minorities (Study 10a)	.53***	.60***
Modern Racism/Relig. Minorities (Study 10b)	.62***	.64***
Attitude Strength (Study 10a)	.09	.39***
Attitude Strength (Study 10b)	.06	.41***
Dogmatism (Study 11)	.21***	.29***
Need for Closure (Study 11)	.23***	.11^
**AVERAGE EFFECT SIZE**	**.30*** **	**.35*** **

Notes: All metrics = standardized *betas*. ***p <= .001; **p <= .01;*p <= .05, ^p <= .15. Across-Group LWA correlations always control for political ideology (every study) + any available demographic factors. [a]Reverse-scored and re-named so that positive correlations always equal the conceptually-expected direction for LWA.

Study 12: Expanded Methods, Results, and Discussion

Much of the debate around LWA has centered only on authoritarianism in Western democracies. However, this WEIRD group of participants is not representative of the whole earth's population (Henrich et al., 2010), and much more work is needed on individual differences in particular in non-WEIRD samples (Cooper, 2016). As a result, it is important to evaluate the LWA question in samples beyond the U.S. This is especially true as there are reasons to expect that left-wing authoritarianism might be more prominent in non-Western contexts (e.g., De Regt et al., 2011; Jost et al., 2003; McFarland et al., 1992, 1993; Todosijević, 2005; Todosijević & Enyedi, 2008).

One method of evaluation is to find validated comparative measurements across nations that attempt to measure both ideological self-identification and authoritarianism with as little measurement cross-contamination as possible. This requires a left-right self-identification measurement that does not contain potential authoritarian content (see Houck & Conway, 2019, for discussion) and an authoritarianism measurement that does not contain explicit ideological content.

While perfectly non-contaminated measurements are essentially impossible – especially for a domain-specific measurement like authoritarianism (Conway, 2020) – some measurements are more bias-free than others. One method of reducing the ideological content in authoritarianism measures is to use generic statements that focus on governmental leadership in a non-partisan way (e.g., Sprong et al., 2019). It is worth noting that these measurements are not ideological content-free; at a minimum, they contain implied ideological content *to participants*. For example, when participants are asked to report agreement with the statement "Our country needs a strong leader right

now" (e.g., Sprong et al., 2019), it very likely matters to participants whether or not they imagine a person whose political views they agree with is the *strong leader* in question. If conservative persons imagine that the strong leader in question is liberal, it would very likely change their answers to the question (compared to believing that the strong leader was conservative). If liberal persons imagine that the strong leader in question is conservative, it would very likely change their answers (compared to believing that the strong leader was liberal).

Thus, such generic language does not produce ideological content-free measurements. However, in a large multi-national study, it does have the advantage of allowing participants themselves across a large number of political contexts to self-determine their own views of leadership and ideology. If this kind of item were collected in only one context, it is likely just as ideologically biased as any other kind of measure. However, when averaged across multiple contexts that vary in the ideological bent of the political leadership (and thus likely vary in the way the item maps on to participant beliefs about the ideological bent of the hypothetical person in the question), it allows for a more (though hardly perfect) ideological content-free test.

In Study 12, we use a generic measurement of governmental authoritarianism similar to that in Sprong et al. (2019) to estimate the worldwide effect of ideology on authoritarianism. This authoritarianism measure was completed in Wave 6 of the World Values Survey (WVS; Inglehart et al., 2014). Specifically, over 66,000 participants across 54 nations completed a standard Political identification (left-right) item (e.g., Jost et al., 2003; Sprong et al., 2019; Nilsson & Jost, 2020) and a standard Authoritarian Governance endorsement questionnaire (e.g., Ariely & Davidov, 2010; Miller, 2017;

Malka, Lelkes, Bakker, & Spivack, 2020). The political identification item allows participants to self-identify on the left or right, offering no direct method overlap with authoritarianism. Further, the authoritarianism scale does not directly offer clearly left-right political positions, but rather asks participants about the degree that they would support various authorities countermanding normal governmental processes. Thus, measured in contexts with varying levels of governmental ideologies, these measurements help define the relationship between authoritarianism and ideology across 54 nations on 5 continents in a way that minimizes ideological cross-contamination.

Prior research across twenty-eight nations (Sprong et al., 2019) using a generic authoritarian leadership measurement (similar to that used in the present study) found a small-to-moderate association between authoritarianism and conservative political orientation (r = .20). Although not designed to specifically test the relationship between authoritarianism and conservative political orientation, this study nonetheless provides a useful starting point. However, it has a limited number of nations and does not have a strong sampling of nations that might be especially prone historically to LWA (e.g., Eastern Europe and former Soviet Republics). In the present study, we nearly doubled the nation-level sample size and included more areas for which LWA might be more manifest.[14] We used two different approaches – multilevel modeling and standardized

[14] In a separate study, Napier and Jost (2008) had 19 democratic (mostly Western) countries from Wave 4 of the World Values Survey. However, not only does their study specifically only focus on a region of the world where one would expect LWA to be lowest (Western democracies) and thus does not advance our knowledge very far beyond WEIRD samples, their measurement of authoritarianism was poor on multiple levels. In addition to including only dichotomous responses, it was loaded with conservative content issues, such as those involving obedience to parents and approaches to single parenting. It further included two items related to general cynicism/trust that are conceptually orthogonal to belief in specific authority figures. In spite of these reasons to distrust the results as a biased approach to the question of LWA, they nonetheless found generally only small-to-moderate effect sizes for the conservatism-authoritarianism

within-country analyses – to estimate the worldwide effects of ideology on authoritarianism.

For reasons outlined by other researchers (Conway, McFarland et al., 2021; Jost et al., 2003; Malka et al., 2020) and consistent with prior data (Sprong et al., 2019), we expected that in general, there would be a positive association between conservative ideological identification and authoritarianism across the world. However, we also expected that this effect would be moderated by the national political context, such that some nations would show less evidence of purely conservative authoritarianism. For example, we expected that this relationship between conservatism and endorsement of government authoritarianism would be less positive in contexts that had a history of influence by left-wing authoritarian governments (e.g., De Regt et al., 2011; Jost et al., 2003; McFarland et al., 1992, 1993; Todosijević, 2005; Todosijević & Enyedi, 2008). Study 12 thus provides novel evidence to more clearly evaluate the state of world authoritarianism on a comparable set of authoritarianism and ideology questionnaires in 54 countries. To our knowledge, this is by far the largest study on world-wide authoritarianism to date.

Method

Participants. For Wave 6 of the WVS, 66,974 participants across 54 nations completed Ideology and Authoritarianism questionnaires.

---

relationship – in the exact region of the world where effect sizes for RWA should be highest. As a result, even taken at face value, they largely corroborate the message of the paper here.

Ideological Conservatism. Participants were asked to position themselves on a 1-10 political left-right continuum, where 1 = "Left" and 10 = "Right." (Participants who responded with answers not placing themselves on the continuum were dropped).

Authoritarianism. Participants completed a three-item measure of endorsement of Authoritarian Governance that has been used in prior research to measure authoritarianism (e.g., Ariely & Davidov, 2010; Miller, 2017; Malka et al., 2020). These items ask participants the degree that they value "Having a strong leader who does not have to bother with parliament and elections," "Having experts, not government, make decisions according to what they think is best for the country," and "Having the army rule." The items were on a scale from 1-4 where 1 = more agreement; as a result, we reversed-scored them and averaged them into a single *Authoritarianism* measure in a manner identical to prior research (e.g., Ariely & Davidov, 2010; Miller, 2017; Malka et al., 2020). (Participants who responded with answers not placing themselves on the continuum were dropped).[15]

Western Democracies Versus Eastern Europe. To evaluate cross-cultural differences, we further compared the ideology-authoritarianism relationship across available Western democracies (defined in the typical manner as the EU15 plus Australia, Canada, New Zealand, Norway, Switzerland, and the United States; see Malka et al., 2020) and a region long influenced by more authoritarian left-wing ideology: Eastern Europe (see INSOL, 2020) and/or the former Soviet Republics (taken together, in our sample this list includes Bulgaria, Czech Republic, Estonia, Hungary, Latvia, Lithuania,

[15] While none of these items is entirely ideologically-free, of these three items, the most clearly ideologically-free item is item one (about a desire for a strong leader). Thus, we further computed all analyses on this item only. Those analyses are identical, both descriptively and inferentially, as those reported for the whole scale.

Moldova, Poland, Kosovo, Moldova, Romania, Russia, Serbia, Slovenia, Kazakhstan, Kyrgyzstan, Tajikistan, Turkmenistan, Uzbekistan, Armenia, Azerbaijan, Georgia, and Ukraine).

Results

The results across the world are graphically depicted in Figure 4. As can be seen there, much variability across the world exists in the degree that authoritarianism leans left (blue) versus right (red).

Figure 4: Left-Wing Authoritarianism (Blue) Versus Right-Wing Authoritarianism (Red) Leanings Around the World

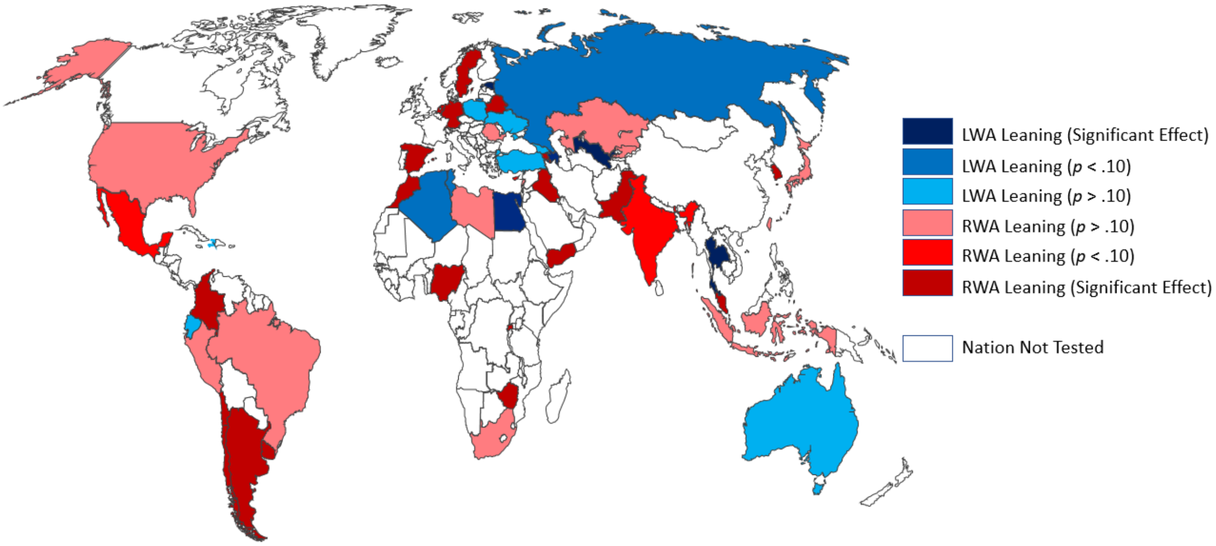

We used several different statistical approaches to better understand this graphical representation.

Multilevel Analyses. We followed standard practices for Multilevel Analyses (e.g., Lorah, 2018; Sprong et al., 2019). Specifically, we used *R* (R Core Team, 2014) to estimate multilevel models with the *lme4* package (Bates et al., 2015). Our primary model (Model 2) predicted authoritarianism from ideology nested within countries.

In Model 1, we first estimated the effect of our level 2 predictor (group: country) on authoritarianism. This predictably showed that nations differed from each other in their levels of authoritarianism, *ICC* = .18, $p < .001$. In Model 2, we then added our level 1 predictor (conservative ideology) to the level 2 predictor (country). Consistent with prior researchers' assertions about the general right-leaning nature of authoritarianism (e.g., Conway et al., 2020; Napier & Jost, 2008; Sprong et al., 2019), this multilevel analysis revealed a positive relationship between conservative ideology and authoritarianism worldwide, *beta* = .01, $p < .001$. However, this relationship is very small and, as we will see below, within-country analyses clearly showed much variability across the world in the ideology-authoritarianism relationship.

Within-country analyses. A second (and related) method of evaluating the worldwide status of the ideology-authoritarianism relationship involves performing within-country analyses. For these analyses, both ideology and authoritarianism scales were first standardized within-country. As a result, any reported summary relationships represent the average within-country effect and thus directly control for across-country mean differences.

More specific results of within-country analyses are presented in Table X. In that table, positive *betas* between authoritarianism and conservative ideology indicate a right-wing authoritarian leaning, while negative *betas* between authoritarianism and ideology indicate a left-wing authoritarian leaning (a *beta* of zero means that ideology and authoritarianism are unrelated, and thus the nation does not show a propensity towards either right-wing or left-wing authoritarianism on average).

First, results averaged across nations were consistent with the Multilevel Modeling analyses: There was a small, but statistically significant, positive relationship between authoritarianism and conservative ideology worldwide, *beta*[66974] = .03, $p < .001$. Thus, two different methods of estimating the worldwide effect of ideology on authoritarianism validated prior researchers' assertions that authoritarianism tends to be right-leaning (e.g., Conway et al., 2020; Napier & Jost, 2008; Sprong et al., 2019).

However, not only was this relationship negligibly small, within-country analyses clearly showed much variability across the world in the ideology-authoritarianism relationship. As Figure 4 and Table X reveal, many nations – particularly those in Western Europe and South America – showed positive and statistically significant relationships between political conservatism and authoritarianism. However, as the dark blue on Figure 4 and the bottom portion of Table X reveal, many nations showed positive and statistically significant relationships of authoritarianism with political *liberalism* (as indicated by the negative relationships between the political conservatism scale and authoritarianism measurement).

Importantly, Table X also reveals that these differences across nations in their propensity for authoritarianism to lean left or right are not likely an artifact of mean or SD differences across nations. Indeed, using nation as the unit of analysis ($n = 54$), the correlations between the ideology-authoritarianism *beta* and (a) country-level mean authoritarianism, (b) country-level SD for authoritarianism, (c) country-level mean ideology, and (d) country-level SD for ideology were all non-significant (*r*'s range from -.25 to .09), and that was also true if one considers the ideology-authoritarianism effect as an absolute value (*r*'s range from -.15 to .11). These additional results suggest there is

real (and not artifactual) variability across countries in their likelihood of showing a conservatism-authoritarianism link.[16]

To understand part of this variability, we compared Western democracies with a region long influenced by more authoritarian left-wing ideology (Eastern Europe and former Soviet Republics). Specifically, after standardizing both authoritarianism and ideology within-nation, we ran a regression with National Context (Western Democracies versus Eastern Europe/Former Soviet Republics) and Ideology predicting Authoritarianism. Consistent with expectations, a National Context X Ideology interaction emerged (interaction *beta*[22,673] = -.10, UCI = -.13, LCI = -.07, $p <$ .0001).[17] This interaction resulted from a significantly positive relationship between conservatism and authoritarianism for those living in Western democracies (*beta* = .09, p < .0001), but little to no relationship for those living in Eastern Europe/Soviet Republics (*beta* = -.01, p = .14).

Discussion

These results reveal that authoritarianism is present on both the right *and* the left side of the political spectrum around the world. Using two different methods of estimating the average effect across 54 nations (and over 66 thousand persons), we found that the overall relationship between conservative ideology and desire for authoritarian

[16] Bolstering this case, we further divided persons up categorically into those that leaned left (below the midpoint of the scale) and those that leaned right (above the midpoint of the scale). As in Conway et al. (2018), analyses with this categorical variable suggests that our findings are not likely due to a correlational sleight-of-hand that is driven by generally higher levels of conservatism world-wide. Indeed, not only is this sample only slightly right-leaning (ideology scale mid-point = 5.5; worldwide M = 5.66), making such an alternative explanation unlikely, but also the highest level of authoritarianism in the world in this sample occurred for *liberals* in Egypt (Egypt Liberal Authoritarianism M = 3.64; worldwide Authoritarianism M = 2.32).

[17] This interaction remained significant (and in the same direction) when using the categorical left/right distinction as the ideology measurement (F = 9.98, p < .002).

government is very small. Further, in many nations, authoritarians were significantly more likely to occur on the *left* side of the political spectrum (see the bottom portion of Table X). It is noteworthy that a right-leaning correlation between ideology and authoritarianism has been interpreted as evidence of *right-wing* authoritarianism (Nilsson & Jost, 2020); thus, applying an equal and fair scientific standard, it is reasonable to interpret a left-leaning correlation between ideology and conservatism as evidence of *left-wing* authoritarianism. Given this, the left-leaning relationships reported at the bottom of Table X suggest clear (and statistically significant) evidence for left-wing authoritarianism in multiple nations. Additionally, the conservatism-authoritarianism relationship is stronger on average in contexts where one might expect it to be stronger (Western democracies), and weaker on average in contexts where one might expect it to be weaker (Eastern Europe/former Soviet Republics).

The relationship between a desire for authoritarian government and ideology in each nation is doubtless influenced by a complicated and ever-changing interplay of the sitting government's ideology, the history of left-wing (versus right-wing) authoritarian movements in a nation, ongoing cultural movements related to authoritarianism on each side of the political spectrum, and nation-level stressors. It is beyond the scope of Study 12 to pursue that more complicated set of factors in depth. Our purpose is less complex but no less important: At a minimum, these results cast doubt on any explanation that leaves *left-wing* authoritarianism out of the world equation. In corroboration with Studies 1-11, they more generally suggest that left-wing authoritarianism is more of a reality than a myth.

<u>Table X</u>

Study 12: Individual-level Conservative/Liberal Ideology Predicting Individual-level Authoritarianism within Nations

Nation	*n*	ID-Auth *Beta*	Ideology Mean	Ideology SD	Auth. Mean	Auth. SD
Yemen	1250	.29***	5.7	2.5	2.3	0.7
Netherlands	1610	.21***	5.5	2.0	2.0	0.5
Argentina	790	.20***	5.5	1.8	2.1	0.7
Chile	701	.19***	5.1	2.0	2.1	0.7
Spain	991	.18***	4.8	1.9	2.1	0.6
Uruguay	833	.18***	4.7	2.5	2.1	0.6
Morocco	197	.16*	5.5	2.3	2.2	0.9
Armenia	1002	.12**	5.7	2.5	2.4	0.7
Columbia	1242	.11***	6.2	2.4	2.5	0.6
Iraq	976	.10***	6.2	2.4	2.4	0.6
Cyprus	824	.09**	5.2	2.7	1.9	0.7
Nigeria	1759	.09*	5.7	2.5	2.4	0.7
Sweden	1118	.08**	5.4	2.5	1.9	0.7
South Korea	1187	.08**	5.4	2.1	2.2	0.5
Malaysia	1300	.08**	6.6	1.9	2.5	0.7
Germany	1829	.07**	5.0	1.8	1.9	0.6
Belarus	1476	.07**	5.4	1.6	2.2	0.5
Pakistan	1172	.07*	7.4	2.0	2.7	0.6
Rwanda	1527	.06*	5.4	1.9	2.3	0.6
Zimbabwe	1499	.05*	5.3	2.7	2.0	0.6
Slovenia	681	.07^	5.1	2.2	2.1	0.5
Mexico	1903	.04^	6.2	2.7	2.6	0.5
Kazakhstan	1500	.04	6.2	2.2	2.3	0.6
Kyrgyzstan	1461	.03	6.5	2.3	2.7	0.6
Lebanon	820	.03	6.4	2.2	2.7	0.7
Libya	1393	.03	5.9	2.7	2.5	0.7
South Africa	3003	.02	6.3	2.1	2.6	0.8
United States	2136	.02	5.8	2.0	2.0	0.7
India	3329	.02^	5.7	2.3	2.7	0.8
Taiwan	1125	.02	4.6	1.9	2.4	0.6
Romania	1082	.02	5.7	2.7	2.8	0.7
Philippines	1187	.02	6.8	2.7	2.5	0.8
Brazil	1199	.01	5.3	2.8	2.7	0.6
Ghana	1552	.01	5.4	2.7	1.9	0.6
Japan	1674	.01	5.6	1.9	1.9	0.6
Peru	1009	.00	5.5	2.2	2.5	0.6
Haiti	1940	-.01	2.7	2.4	1.9	0.6

Turkey	1368	-.01	6.4	2.4	2.4	0.8
Australia	1404	-.02	5.3	2.0	1.9	0.6
Ecuador	1139	-.03	5.6	2.5	2.4	0.6
Georgia	778	-.03	5.6	2.2	2.2	0.7
Palestine	720	-.03	6.0	2.3	2.4	0.7
Ukraine	1500	-.03	5.5	1.9	2.4	0.6
Poland	741	-.03	5.5	2.3	2.3	0.5
Russia	1441	-.04^	5.4	2.1	2.5	0.6
Tunisia	696	-.06^	5.6	1.8	2.6	0.8
Algeria	1041	-.08^	6.0	2.1	2.0	0.8
Trinidad and Tobago	561	-.08^	6.4	2.4	1.8	0.7
Hong Kong	974	-.08**	5.4	1.7	2.1	0.6
Thailand	1187	-.09**	5.9	2.2	2.1	0.7
Azerbaijan	991	-.10**	5.9	2.0	2.2	0.6
Uzbekistan	777	-.19***	6.5	2.1	2.5	0.9
Estonia	1254	-.16***	5.4	1.9	2.1	0.6
Egypt	1523	-.22***	6.1	2.3	3.5	0.6
**TOTAL**	**66974**	**.03*** **	5.7	2.4	2.3	0.7

Notes: *ID-Auth Beta* = standardized *beta* for conservative ideology-authoritarianism relationship; both measures standardized within-nation. ***p <= .001; **p <= .01;*p <= .05, ^p <= .10. *For ID-Auth Beta*, Positive scores = right-wing authoritarianism more prevalent; negative scores = left-wing authoritarianism more prevalent. Means and SDs are unstandardized scores.

Expanded General Discussion

Is left-wing authoritarianism a viable construct that predicts important real-world phenomena? Across twelve studies spanning over 8,000 participants in the U.S. and over 66,000 participants worldwide, our data consistently reveal the answer is *yes*. These data reveal that (1) both liberal and conservative American participants identify a large number of left-wing authoritarians in their everyday lives (Study 1), and (2) both liberal and conservative participants rate a common Left-Wing Authoritarianism scale as measuring authoritarianism (Study 2). Further, this same LWA scale consistently predicts key phenomena that major authoritarianism theories suggest it should predict, including (3) threat sensitivity (Studies 3-6), (4) restrictive communication norms (Study 7), (5)

negative ratings of minority groups (Studies 8-10), and (6) dogmatism (Study 10). Further, we used multiple methods to help overcome the double-barreled measurement problem inherent in *any* authoritarianism measurement, including controlling directly for ideology (Studies 3-11), comparing parallel RWA and LWA effects (Studies 3-11), performing analyses only on liberals (Studies 3-11), and including a more generic measurement of authoritarian leadership across numerous national contexts (Study 12). Each of these approaches has offsetting strengths and weaknesses, and yet they all point to the same conclusion: This wide array of triangulating evidence provides consistent support for the idea that left-wing authoritarianism is indeed a widespread everyday reality.

Below, we place this array of evidence into the existing literature on authoritarianism and ideology, discuss limitations of our work, and offer a brief set of concluding thoughts.

The Authoritarianism Debate

The present studies have multiple implications for the ongoing debate about the nature of authoritarianism. We here highlight two: (1) LWA is not merely about ideology, and (2) LWA, far from muddying the authoritarianism waters, actually provides insight that helps us better understand authoritarianism in the bigger picture.

LWA measurement is not merely an ideology measurement. Nilsson and Jost (2020) have argued that prior evidence based on Conway et al's (2018) LWA scale was due to its overlap with liberal ideology, and thus it did not provide empirical evidence of liberal authoritarianism.[18] The issue raised by this critique is important. What *do* more

---

[18] Nilsson and Jost (2020) recommend solving this problem by using a different scale they claim is more

focused empirical tests – tests based in long-accepted scientific practice – reveal? Our multi-method evidence here suggests that, in fact, the scale *is* measuring something beyond *mere* liberalism. Almost all key effects across Studies 3-11 remain when controlling for political ideology. Further, in a similar fashion, almost all key effects remain within-liberals: Thus, when comparing liberal authoritarians to liberal non-authoritarians, high-LWA persons show conceptually-expected correlations. As a result, the scale differentiates one kind of liberal from another kind, and thus cannot be reduced to *mere* ideology.

This array of evidence overwhelmingly suggests that, contrary to critics' claims, there is something beyond mere ideology captured by the LWA scale. What *is* that *something beyond*? Consistent with a long line of research on RWA, by far the most parsimonious answer to that question is that the *something beyond* is *authoritarianism*. And indeed, using standard content validity approaches also used in other authoritarianism work (e.g., Funke, 2005; Dunwoody & Funke, 2016), Study 2 showed that participants evaluate the items in Conway's LWA scale as measurements of *authoritarianism.* This strong empirical evidence is echoed in the judgments of researchers Fasce and Avendaño (2020, p. 3), who commented that the items on Conway et al's LWA scale "are not merely statements of liberal ideology; they univocally reflect

"value neutral": Dunwoody and Funke's Aggression-Submission-Conventionalism (2016) scale. This recommendation is curious because Dunwoody and Funke's (2016) scale is highly unlikely to be value-neutral, as evidenced by the fact that it is extremely highly correlated with Altemeyer's RWA scale (which those authors acknowledge is clearly *not* value-neutral) or scales based on Altemeyer's scale: One early version of the ASC scale correlated at $r = .81$ with Altemeyer's RWA scale, and the final ASC correlated at .71 and .73 in two samples with an RWA scale (Funke, 2005) based off of Altemeyer's scale (for details, see Dunwoody & Funke, 2016). At a minimum, *far* more work needs to be done to establish a genuinely value-neutral scale where participants are not imagining conservative leaders as they complete the scale. And that work has to be placed against the large range of work described here, where efforts *were* made to produce a genuinely balanced approach to the LWA/RWA question using standard social psychological methods.

an extremely authoritarian attitude, opposed to liberal commitments such as equality among citizens, freedom of expression, and tolerance toward political and cultural diversity."

Taken together, this array of triangulating evidence points to the conclusion that – as is the case for the scientific consensus on the Altemeyer RWA scale on which it was based – Conway et al's LWA scale is a valid measurement of authoritarianism.

LWA helps *un*confound ideology from authoritarianism. In commenting on the LWA scale, Nilsson and Jost (2020) argued that "they [Conway et al, 2018] deliberately confounded authoritarian inclinations and support for liberal (as opposed to conservative) opinions and groups in society." The present work helps illustrate that this criticism is misguided. As laid out in Conway et al's (2018) original LWA paper, they did indeed deliberately include parallel ideological content in their LWA scale. However, as they noted, they were not trying to deliberately *confound* ideology and authoritarianism, but deliberately trying to *un*confound them. Indeed, Conway et al (2018) were attempting (and, as our work here across all studies supports, succeeding) to help solve the very problem those authors critical of LWA themselves recently raised with Altemeyer's RWA scale (from Nilsson & Jost, 2020):

> *"For example, Altemeyer's RWA scale contains items such as 'You have to admire those who challenged the law and the majority's view by protesting for women's abortion rights, for animal rights, or to abolish school prayer' (reverse-scored) and 'God's laws about abortion, pornography and marriage must be strictly followed before it is too late, and those who break them must be strongly punished'. These items conflate authoritarian attitudes with conservative*

> *positions on specific issues, such as women's rights, abortion, and marriage equality. This content overlap could produce a spurious correlation between authoritarianism and conservatism."*

We agree. As we have laid out here, among the ways to try and better understand authoritarianism distinct from ideology is to produce equivalent parallel scales that use similar authoritarian language on both sides of the ideological spectrum. Conway et al (2018)'s scale – unlike Altemeyer's early LWA scale – used language that created straightforward equivalence across the scales. While no endeavor is perfect – and the nature of the task means it is impossible to produce exactly comparable scales (by definition, a left-wing authoritarian scale contains left-wing content, and a right-wing authoritarianism scale contains right-wing content) – nonetheless, the overwhelming amount of empirical evidence presented in Studies 2-12 suggests Conway et al's (2018) LWA scale is a valid measurement of liberal authoritarianism. Further, this evidence has to be taken in context with other evidence showing the predictive validity of Conway et al's LWA scale for (1) voting intentions in the 2016 election (Conway & McFarland, 2019), (2) informational contamination, reactance, and support for divisive behaviors (Conway, Houck et al., 2021), and (3) a reactive stance against reward for application (Fasce & Avendaño, 2020).

Importantly, this approach has import not just for our understanding of *left-wing* authoritarianism, but also authoritarianism more generally. In fact, taken at face value, Nilsson and Jost's (2020) critique of Altemeyer calls into question decades of RWA research that has established the fundamentals of the authoritarian person that are now largely accepted as axiomatic by the field at large. Is it possible that all the research

showing that, for example, authoritarians are threat sensitive really just means that *conservatives* are threat sensitive? If so, then we have learned nothing about authoritarianism. The present work helps provide a more complete picture by considering directly what an authoritarian on the other side of the political aisle might look like, thus helping us separate authoritarianism from ideology. When we do that, we see that authoritarianism on the left shares some similar properties with authoritarians on the right – for example, they both show heightened sensitivity to threat. As a result of this, we can more confidently isolate the effects of authoritarianism as an ideologically embedded theoretical construct.

The Ideological Asymmetry Debate

The present results further fit into a growing literature showing that negatively-valenced outcomes once believed to asymmetrically apply to conservatives can in fact sometimes apply to liberals as well (e.g., Clark & Winegard, 2020; Crawford, 2017; Ditto et al., 2019; Duarte et al., 2015; Eadeh & Chang, 2019; Fiagbenu et al., 2019; Frimer et al., 2017; Honeycutt & Jussim, 2020; Jussim et al., 2015; Jussim et al., 2016; Proch et al., 2018). For example, Fiagbenu and colleagues (2019) recently showed that, while in line with traditional asymmetrical conceptions of ideology, conservatives showed more learning of negative stimuli in a "BeanFest" game, liberals actually showed more learning of negative stimuli when the same scenario was re-framed as a "StockFest" game. Similarly, work reveals that in some circumstances, liberals (versus conservatives) can show less acceptance of attitudinal ambivalence (Newman & Sargent, 2020; Sargent & Newman, 2020), more desire for social stability (Proch et al., 2018), less cognitive complexity (Conway et al., 2016; Houck & Conway, 2019), more dogmatism (Conway et

al., 2016), more negative outgroup attitudes (Chambers et al., 2013), and more ingroup bias (Ditto et al., 2019).

The present results directly contribute to that debate by demonstrating that authoritarianism – once largely confined to the right side of the political spectrum – can also be found in general populations on the left side of the political spectrum. However, we must be cautious in over-interpreting this as perfect evidence of authoritarian symmetry, whereby we assume that liberals and conservatives are essentially equal with respect to authoritarianism. Indeed, there are at least three interpretations which we do *not* make.

First, we are not claiming that there are equal numbers of authoritarians among both liberals and conservatives. At this point, we simply do not have enough data to know the answer to that question for certain – but we agree with Jost et al. (2003) that there are reasons to expect authoritarianism to appear more frequently on the right than on the left. As a result, in claiming that there are a large number of measurable left-wing authoritarians world-wide, we are not claiming that they are of an *equal* number as right-wing authoritarians. We do not know for sure, but we rather suspect that conservative authoritarians will remain more prominent worldwide for much the same reasons detailed by Jost et al. (2003) – and although our own data suggest plenty of evidence of LWA, they also suggest a small (but real) tendency for RWA to be more prominent.

Second, we do not mean that specific authoritarians on the left and the right will be identical in every respect. Indeed, that would be highly improbable given the differences in liberals and conservatives more broadly. Rather, our data suggests that left-wing authoritarians share the basic properties of an authoritarian person, and as such are

similar to right-wing authoritarians in those properties. Thus, it is reasonable to call someone scoring high on the LWA scale an “authoritarian” in the same way that it is reasonable to call someone scoring high on the RWA scale an “authoritarian.” However, we would expect them to be different in multiple ways too, and parsing out those similarities and differences is an important task for future research (Chan et al., 2018). To name just one potential example, Conway, McFarland et al (2021) propose an Authoritarian Norm Fit Model. Among other things, this model proposes that in the U.S., a liberal authoritarian may feel that “authoritarianism” is less congruent with their own ingroup norms than a conservative authoritarian. Thus, a liberal authoritarian in the U.S. may be analogous to an introvert living in a house of extraverts, while a conservative authoritarian may be like an introvert living in a house of introverts. Both persons are comparably equally introverted (or authoritarian), and yet those two people should differ on measurable qualities as well.

Finally, we also must be careful to not present this debate as a false dichotomy between perfect symmetry and invalidity. As we have outlined, there almost certainly will be some asymmetry between LWA and RWA, either in the frequencies those persons occur, their psychological properties, or both. However, these asymmetries do not undermine the larger empirical validity and theoretical relevance of the LWA construct. Because one can identify state-or-trait asymmetries between two introverts does not mean either of them is *not introverted*. That is a separate question that is dependent on the specific properties of the introvert. As we have shown in Studies 1-13, there is ample evidence to believe that authoritarians *exist* on the left side of the political spectrum just as they do on the right side.

Limitations

Like all studies, the present study has limitations. First, although employing much larger and more diverse samples than most previous work on authoritarianism, Studies 1-12 (like much prior authoritarianism research) are nonetheless limited to the United States and should not be taken to generalize beyond that region.

Further, as other researchers have noted (Nilsson et al., 2020), the Conway et al (2018) scale on which Studies 2-12 are based is not perfect. However, essentially all critiques of individual items on the scale hinge on the argument that these items do not measure anything beyond left-wing ideology.[19] As such, all these smaller critiques are best addressed with triangulating empirical evidence that the whole collection of items – used in the way originally intended by the authors of the scale, as a total summative measure – is in fact capturing something beyond mere ideology. Evidence that the whole scale is valid suggests at a minimum that the collection of items as a whole is valid – and thus directly suggests there is no *systemic* problem with items interfering with the validity of the scale. It is just that kind of whole-scale validity evidence that has been supplied across multiple studies in the present package. This empirical approach mirrors the approach in other domains when critiques arise of the empirical validity of particular theoretical constructs (e.g., Banaji et al., 2004).

However, we acknowledge that Conway et al's (2018) LWA scale, like all scales, is not perfect and thus does of course have room for improvement (Conway, 2020). But saying a scale is *imperfect* is not the same as saying a scale is *invalid*. *All* measurements

---

[19] We pause to note that, if this were true, as a measurement of *liberalism* the LWA scale would paint an excessively unflattering portrait of liberals – a portrait that would go against much theorizing about liberalism. Fortunately, our own data suggest this is *not* true, and thus the scale can rather be viewed as a measurement of a particular kind of liberal, and not a more general measurement of liberalism.

contain imperfections and all studies contain messiness, and yet that should not deter us from bigger-picture research conclusions (Cooper, 2016). Thus, we acknowledge the facts that (a) like virtually every scale, the LWA scale could be improved, and (b) as a scale designed to parallel the most widely-used RWA scale, it inherited some of that scale's weaknesses. However, this lack of perfection should not be confused with the larger, big-picture issue of the degree that it can be construed as a valid measurement of left-wing authoritarianism. The overwhelming amount of evidence across multiple studies speaks clearly: It can be accurately viewed as a measurement of left-wing authoritarianism.

Concluding Thoughts

Recent evidence has revealed a need for balanced evaluations of potential symmetries and asymmetries related to political ideology (e.g., Clark & Winegard, 2020; Crawford, 2017; Ditto et al., 2019; Duarte et al., 2015; Eadeh & Chang, 2019; Fiagbenu et al., 2019; Frimer et al., 2017; Honeycutt & Jussim, 2020; Jussim et al., 2015; Jussim et al., 2016; Proch et al., 2018). Using a multi-method approach spanning multiple content areas, validity types, statistical controls, and scale types, the present results consistently show that, just as right-wing persons are sometimes authoritarian, left-wing persons may also be similarly authoritarian. Taken together, this large array of evidence suggests that left-wing authoritarianism is more of a reality than a myth.

References

Altemeyer, R. A. (1996). *The Authoritarian Spectre*. Harvard University Press.

Altemeyer, B. (1998). The other "authoritarian" personality. *Advances in Experimental Social Psychology, 30*, 47-91. http://dx.doi.org/10.1027/1614-0001.27.3.117

Ariely, G., & Davidov, E. (2010). Can we rate public support for democracy in comparable way? Cross-national equivalence of democratic attitudes in the world value survey. *Social Indicators Research, 104*(2), 271-86.

Banaji, M. R., Nosek, B. A., & Greenwald, A. G. (2004). No place for nostalgia in science: A response to Arkes and Tetlock. *Psychological Inquiry, 15*(4), 279-310. https://www.jstor.org/stable/20447240

Bates, D., Maechler, M., Bolker, B., & Walker, S. (2015). Fitting linear mixed effects models using lme4. *Journal of Statistical Software, 67*(1), 1–48. https://doi.org/10.18637/jss.v067.i01.

Baumeister, R. F. (1993). *Self-Esteem*: *The Puzzle of Low Self-Regard*. Plenum Press.

Beall, A. T., Hofer, M. K., & Schaller, M. (2016). Infections and elections: Did an Ebola outbreak influence the 2014 U.S. Federal Elections (and if so, how?). *Psychological Science, 27*(5), 595-605. https://doi.org/10.1177/0956797616628861

Breiman, L., & Freedman, D. (1983). How many variables should be entered in a regression equation? *Journal of the American Statistical Association, 78*(381), 131–136. https://doi.org/10.1080%2F01621459.1983.10477941

Carroll, R., Peresman, A., & Back, H. (2021). Authoritarianism and immigration attitudes in the UK. *Political Studies*.

Chambers, J.R., Schlenker, B.R., & Collisson, B. (2013). Ideology and prejudice: The role of value conflicts. *Psychological Science*, *24*(2), 140-149. https://doi.org/10.1177/0956797612447820

Choma, B. L., & Hanoch, Y. (2017). Cognitive ability and authoritarianism: Understanding support for Trump and Clinton. *Personality and Individual Differences*, *106*, 287-291. https://doi.org/10.1016/j.paid.2016.10.054

Choma, B. L., & Hodson, G. (2017). Right-wing ideology: Positive (and negative) relations to threat. *Social cognition*, *35*(4), 415-432.

Clark, C. J., & Winegard, B. M. (2020). Tribalism in war and peace: The nature and evolution of ideological epistemology and its significance for modern social science. *Psychological Inquiry*, *31*(1), 1-22. https://doi.org/10.1080%2F1047840x.2020.1721233

Clifford, S., Jewell, R. M., & Waggoner, P. D. (2015). Are samples drawn from Mechanical Turk valid for research on political ideology? *Research and Politics, 2*(4), 1–9. https://doi.org/10.1177/2053168015622072

Chan, L., McFarland, J. D., & Conway, L. G., III. (2018). Political contamination of social psychology: A review of Crawford and Jussim's (2017) edited book 'The politics of social psychology.' *Social Justice Research, 31*(3), 323-333. https://doi.org/10.1007/s11211-018-0312-y

Conway, L. G., III. (2020, February 27-29). *Is left-wing authoritarianism real?: Evidence on both sides of the debate* [Symposium]. Society for Personality and Social Psychology, New Orleans, LA, United States.

Conway, L. G., III, Bongard, K., Plaut, V. C., Gornick, L. J., Dodds, D., Giresi, T.,

Tweed, R. G., Repke, M. A., & Houck, S. C. (2017). Ecological origins of freedom: Pathogens, heat stress, and frontier topography predict more vertical but less horizontal governmental restriction. *Personality and Social Psychology Bulletin*, *43*(10), 1378-1398. https://doi.org/10.1177/0146167217713192

Conway, L. G., III, Clements, S. M., & Tweed, R. G. (2006). Collectivism and governmentally initiated restrictions: A cross-sectional and longitudinal analysis across nations and within a nation. *Journal of Cross-Cultural Psychology, 37*(1), 1-23. https://doi.org/10.1177/0022022105282293

Conway, L. G., III, Conway, K. R., Gornick, L. J., & Houck, S. C. (2014). Automated integrative complexity. *Political Psychology*, *35*(5), 603-624. https://doi.org/10.1111/pops.12209

Conway, L. G. III, Dodds, D., Towgood, K. H., McClure, S, & Olson, J. (2011). The biological roots of complex thinking: Are heritable attitudes more complex? *Journal of Personality, 79*(1), 101-134. https://doi.org/10.1111/j.1467-6494.2010.00690.x

Conway, L. G., III, Gornick, L. J., Burfiend, C., Mandella, P., Kuenzli, A., Houck, S. C., & Fullerton, D. T. (2012). Does simple rhetoric win elections? An integrative complexity analysis of U.S. presidential campaigns. *Political Psychology*, *33*(5), 599-618. https://doi.org/10.1111/j.1467-9221.2012.00910.x

Conway, L. G. III, Gornick, L. J., Houck, S. C., Anderson, C., Stockert, J., Sessoms, D., & McCue, K. (2016). Are conservatives really more simple-minded than liberals? The domain specificity of complex thinking. *Political Psychology*, *37*(6), 777-798. https://doi.org/10.1111/pops.12304

Conway, L. G, III, Houck, S. C., Chan, L., Repke, M. A., & McFarland, J. (in press). The agreement paradox: How pressures for agreement can ultimately divide us. In J.-W. van Prooijen (Ed.), *Current Issues in Social Psychology: Political Polarization*. New York: Routledge.

Conway, L. G., III, Houck, S. C., Gornick, L. J., & Repke, M. A. (2016). Ideologically-motivated perceptions of complexity: Believing those who agree with you are more complex than they are. *Journal of Language and Social Psychology, 35*(6), 708-718. https://doi.org/10.1177/0261927x16634370

Conway, L. G., III, Houck, S. C., Gornick, L. J., Repke, M. R. (2018). Finding the Loch Ness Monster: Left-Wing Authoritarianism in the United States. *Political Psychology, 39*(5), 1049-1067. https://doi.org/10.1111/pops.12470

Conway, L. G., III, & McFarland, J. D. (2019). Do Right-Wing and Left-Wing Authoritarianism Predict Election Outcomes?: Support for Obama and Trump Across Two United States Presidential Elections. *Personality and Individual Differences, 138,* 84-87. https://doi.org/10.1016/j.paid.2018.09.033

Conway, L. G., III, McFarland, J. D., Costello, T. H., & Lilienfeld, S. O. (2020). The curious case of left-wing authoritarianism: When authoritarian persons meet anti-authoritarian norms [Manuscript Under Review]. Department of Psychology, University of Montana.

Conway, L. G., III, Repke, M. A., & Houck, S. C. (2017). Donald Trump as a cultural revolt against perceived communication restriction: Priming political correctness norms causes more Trump support. *Journal of Social and Political Psychology, 5*(1), 244-259. https://doi.org/10.5964/jspp.v5i1.732

Conway, L. G. III, Salcido, A., Gornick, L. J., Bongard, K. A., Moran, M., & Burfiend, C. (2009). When self-censorship norms backfire: The manufacturing of positive communication and its ironic consequences for the perceptions of groups. *Basic and Applied Social Psychology, 31*(4), 335-347.https://doi.org/10.1080/01973530903317169

Conway, L. G., III, & Schaller, M. (2005). When authority's commands backfire: Attributions about consensus and effects on deviant decision making. *Journal of Personality and Social Psychology*, *89*(3), 311-326. https://doi.org/10.1037/0022-3514.89.3.311

Conway, L. G., III, & Schaller, M. (2007). How communication shapes culture. In K. Fiedler (Ed.), *Frontiers of Social Psychology: Social communication* (pp. 107-127). Psychology Press.

Conway, L. G., III, Suedfeld, P., & Tetlock, P. E. (2018). Integrative complexity in politics. In A. Mintz (Ed.), *Oxford Handbook of Behavioral Political Science*. Oxford: Oxford University Press. https://doi.org/10.1093/oxfordhb/9780190634131.013.7

Conway, L. G., III., Thoemmes, F., Allison, A. M., Hands Towgood, K., Wagner, M. J., Davey, K., Salcido, A., Stovall, A. N., Dodds, D. P., Bongard, K, & Conway, K. R. (2008). Two ways to be complex and why they matter: Implications for attitude strength and lying. *Journal of Personality and Social Psychology, 95*(5), 1029-1044. https://doi.org/10.1037/a0013336

Conway, L. G., III, Woodard, S. R., & Zubrod, A. (2020). *Social psychological*

measurements of COVID-19: Coronavirus perceived threat, government response, impacts, and experiences questionnaires. PsyArXiv. https://doi.org/10.31234/osf.io/z2x9a

Conway, L. G., III, Woodard, S. R., & Zubrod, A., & Chan, L. *Why are conservatives less concerned about the coronavirus (COVID-19) than liberals?* PsyArXiv https://psyarxiv.com/fgb84/

Conway, L. G., III, Zubrod, A., & Chan, L. (2020). The paradox of the tribal equalitarian. *Psychological Inquiry, 31*(1), 48–52. https://doi.org/10.1080%2F1047840x.2020.1722018

Cooper, M. L. (2016). Personality processes and individual differences. *Journal of Personality and Social Psychology, 110(3)*, 431-434. https://www.apa.org/pubs/journals/features/psp-p0000033.pdf

Costello, T. H., Bowes, S. M., Stevens, S. T., Waldman, I. D., & Lilienfeld, S. O. (2020). *Clarifying the structure and nature of left-wing authoritarianism.* PsyArXiv. https://doi.org/10.31234/osf.io/3nprq

Crandall, C. S., & Sherman, J. W. (2016). On the scientific superiority of conceptual replications for scientific progress. *Journal of Experimental Social Psychology, 66,* 93-99.

Crawford, J. T. (2017). Are conservatives more sensitive to threat than liberals? It depends on how we define threat and conservatism. *Social Cognition, 35*(4), 354-373. https://doi.org/10.1521/soco.2017.35.4.354 https://doi.org/10.1177/0146167207303949

De Regt, S., Mortelmans, D., & Smits, T. (2011). Left-wing authoritarianism is not a

myth, but worrisome reality: Evidence from 13 Eastern European countries. *Communist and Post-Communist Studies*, *44*(4), 299-308. https://doi.org/10.1016/j.postcomstud.2011.10.006

Diamant, J. (2018). Blacks more likely than others in U.S. to read the Bible regularly, see it as God's word. *Pew Research Center.* https://www.pewresearch.org/fact-tank/2018/05/07/blacks-more-likely-than-others-in-u-s-to-read-the-bible-regularly-see-it-as-gods-word/

Ditto, P., Liu, B., Clark, C. J., Wojcik, S., Chen, E., Grady, R. H., Celniker, J. B., & Zinger, J. (2019). At least bias Is bipartisan: A meta-analytic comparison of partisan bias in liberals and conservatives. *Perspectives on Psychological Science, 14*(2), 273-291. https://doi.org/10.1177/1745691617746796

Donnellan, M. B., Oswald, F. L., Baird, B. M., & Lucas, R. E. (2006). The mini-IPIP scales: tiny-yet-effective measures of the Big Five factors of personality. *Psychological Assessment, 18(2),* 192-203. https://doi.org/10.1037/1040-3590.18.2.192

Duckitt, J. (2001). A dual-process cognitive-motivational theory of ideology and prejudice. In M. P. Zanna (Ed.), *Advances in experimental social psychology*, (Vol. 33, p. 41–113). Academic Press.

Duckitt, J. (2013). Introduction to the special section on authoritarianism in societal context: The role of threat. *International Journal of Psychology, 48(1)*, 1-5. doi:http://dx.doi.org.weblib.lib.umt.edu:8080/10.1080/00207594.2012.738298

Duckitt, J., Bizumic, B., Krauss, S. W., Heled, E. (2010). A tripartite approach to right-wing authoritarianism: The authoritarianism–conservatism–traditionalism model

Political Psychology, 31(5), 685–715. https://doi.org/10.1111/j.1467-9221.2010.00781.x

Duckitt, J., Wagner, C., du Plessis, I., & Birum, I. (2002). The psychological bases of ideology and prejudice: Testing a dual process model. *Journal of Personality and Social Psychology, 83*(1), 75–93. https://doi.org/10.1037/0022-3514.83.1.75

Duarte, J. L., Crawford, J. T., Stern, C., Haidt, J., Jussim, L., & Tetlock, P. E. (2015). Political diversity will improve social psychological science. *Behavioral and Brain Sciences, 38*. https://doi.org/10.1017%2Fs0140525x14000430

Dunwoody, P. T., & Funke, F. (2016). The Aggression-Submission-Conventionalism Scale: Testing a new three factor measure of authoritarianism. *Journal of Social and Political Psychology, 4*(2), 571–600. https://doi.org/10.5964%2Fjspp.v4i2.168

Dyrbye, L., Herrin, J., West, C.P., et al. (2019). Association of racial bias with burnout among resident physicians. *Journal of American Medical Association Network Open, 2(7)*. https://doi.org/10.1001/jamanetworkopen.2019.7457

Eadeh, F. R., & Chang, K. K. (2019). Can threat increase support for liberalism? New insights into the relationship between threat and political attitudes. *Social Psychological and Personality Science, 11*(1), 88-96. https://doi.org/10.31234%2Fosf.io%2Fcz35s

Everett, J. A. (2013). The 12 item Social and Economic Conservatism Scale (SECS). *PLoS ONE, 8,* e82131.

Eysenck, H. J. (1954). *Psychology of Politics*. Routledge & Kegan Paul.

Fasce, A., & Avendaño, D. (2020). Opening the can of worms: A comprehensive

examination of authoritarianism. *Personality and Individual Differences, 163*, 1-9. https://doi.org/10.1016/j.paid.2020.110057

Federico, C. M., Fisher, E. L., & Deason, G. (2017). The authoritarian left withdraws from politics: Ideological asymmetry in the relationship between authoritarianism and political engagement. *Journal of Politics*, *79*(3), 1010-1023. https://doi.org/10.1086/692126

Feldman, S. (2001). Authoritarianism. In N. J. Smelser & P. B. Baltes (Eds.), *International encyclopedia of the social & behavioral sciences* (p. 971). Elsevier.

Feldman, S. (2003). Enforcing social conformity: A theory of authoritarianism. *Political Psychology*, *24*(1), 41-74. https://doi.org/10.1111/0162-895X.00316

Fiagbenu, M. E., Proch, J., & Kessler, T. (2019). Of deadly beans and risky stocks: Political ideology and attitude formation via exploration depend on the nature of the attitude stimuli. *British Journal of Psychology*. https://doi.org/10.1111/bjop.12430

Fincher, C. L., & Thornhill, R. (2012). Parasite-stress promotes in-group assortative sociality: The cases of strong family ties and heightened religiosity. *Behavioral and Brain Sciences, 35*(2), 61–79. https://doi.org/10.1017%2Fs0140525x11000021

Frimer, J. A., Gaucher, D., & Schaefer, N. K. (2014). Political conservatives' affinity for obedience to authority is loyal, not blind. *Personality and Social Psychology Bulletin, 40*(9), 1205- 1214. https://doi.org/10.1177%2F0146167214538672

Frimer, J., Skitka, L. J., & Motyl, M. (2017). Liberals and conservatives are similarly motivated to avoid exposure to one another's opinions. *Journal of Experimental*

Social Psychology, 72, 1-12. https://doi.org/10.1016/j.jesp.2017.04.003

Funke, F. (2005). The dimensionality of right-wing authoritarianism: Lessons from the dilemma between theory and measurement. *Political Psychology, 26*(2), 195-218. https://doi.org/10.1111/j.1467-9221.2005.00415.x

Górska, P., Stefaniak, A., Lipowska, K., Malinowska, K., Skrodzka, M., & Marchlewska, M. (2021). Authoritarians Go with the Flow: Social Norms Moderate the Link between Right-Wing Authoritarianism and Outgroup-Directed Attitudes. *Political Psychology*.

Gries, T., Mueller, V., & Jost, J. (2021). The market for belief systems: A formal model of ideological choice. *Psychological Inquiry*.

Hayes, A. F. (2017). *Introduction to mediation, moderation, and conditional process analysis: A regression-based approach*. Guilford publications.

Hayes, A. F., & Rockwood, N. J. (2020). Conditional process analysis: Concepts, computation, and advances in the modeling of the contingencies of mechanisms. *American Behavioral Scientist, 64*(1), 19–54. https://doi.org/10.1177/0002764219859633

Henrich, J., Heine, S. J., & Norenzayan, A. (2010). The weirdest people in the world?. *Behavioral and Brain Sciences, 33*(2-3), 61-83. https://doi.org/10.2139/ssrn.1601785

Honeycutt, N., & Jussim, L. (2020). A model of political bias in social science research. *Psychological Inquiry, 31*(1), 73-85. https://doi.org/10.1080/1047840x.2020.1722600

Houck, S. C., & Conway, L. G. III. (2019). Strategic communication and the

integrative complexity-ideology relationship: Meta-analytic findings reveal differences between public politicians and private citizens in their use of simple rhetoric. *Political Psychology*, *40(5),* 1119-1141. https://doi.org/10.1111/pops.12583

Houck, S. C., Conway, L. G., III, & Gornick, L. J. (2014). Automated integrative complexity: Current challenges and future directions. *Political Psychology, 35*(5), 647-659. https://doi.org/10.1111/pops.12209

Houck, S. C., Conway, L. G., III, & Repke, M. (2014). Personal closeness and perceived torture efficacy: If torture will save someone I'm close to, then it must work. *Peace and Conflict: Journal of Peace Psychology, 20*(4), 590-592. https://doi.org/10.1037/pac0000058

Houck, S. C., Conway, L. G. III, Parrow, K., & Luce, A., & Salvati, J. (2018). An integrative complexity analysis of religious and irreligious thinking. *Sage Open, 8*(3),1-12. https://doi.org/10.1177/2158244018796302

Houck, S. C., Repke, M. A., & Conway, L. G., III. (2017). Understanding what makes terrorist groups' propaganda effective: An integrative complexity analysis of ISIL and Al Qaeda. *Journal of Policing, Intelligence and Counter Terrorism, 12*(2), 105-118. https://doi.org/10.1080/18335330.2017.1351032

INSOL. (2020). *Eastern European Countries: Committee Introduction and Members.* https://www.insol-europe.org/eastern-european-countries-committee-introduction-and-members#:~:text=Thanks%20to%20the%20existence%20of,Serbia%2C%20Slovak%20Republic%20and%20Slovenia.

Inglehart, R., C. Haerpfer, A. Moreno, C. Welzel, K. Kizilova, J. Diez-Medrano, M.

Lagos, P. Norris, E. Ponarin & B. Puranen et al. (Eds.). 2014. World Values Survey: Round Six - Country-Pooled Datafile Version: https://www.worldvaluessurvey.org/WVSDocumentationWV6.jsp. Madrid: JD Systems Institute.

Jost, J. T., Nosek, B. A., & Gosling, S. D. (2008). Ideology: Its resurgence in social, personality, and political psychology. *Perspectives on Psychological Science*, *3*(2), 126-136. https://doi.org/10.1111/j.1745-6916.2008.00070.x

Jost, T., Glaser, J., Kruglanski, A. W., & Sulloway, F. J. (2003). Political conservatism as motivated social cognition. *Psychological Bulletin 129*(3), 339-375. https://doi.org/10.4324/9781315175867-5

Jussim, L., Crawford, J.T., Stevens, S. T., & Anglin, S. M (2016). The politics of social psychological science: Distortions in the social psychology of intergroup relations. *Social psychology of political polarization* (P. Valdesolo, and J. Graham, eds, pp. 165-196). Routledge.

Jussim, L., Crawford, J.T., Anglin, S. M., & Stevens, S. T. (2015). Ideological bias in social psychological research. In J. Forgas, W. Crano, & K. Fiedler (Eds.), *Social Psychology and Politics* (pp. 91-109). Taylor & Francis.

Karwowski, M., Kowal, M., Groyecka, A., Bialek, M., Lebuda, I., Sorokowska, A., & Sorokowski, P. (2020). When in danger, turn right: Does Covid-19 threat promote social conservatism and right-wing presidential candidates? *Human Ethology, 35*(1), 37-48. https://doi.org/10.22330/he/35/037-048

Kennedy, R., Clifford, S., Burleigh, T., Jewell, R., & Waggoner, P. (2018). The shape of and solutions to the MTurk quality crisis. *Political Science Research and Methods*, *8*(4), 614-629. *https://doi.org/10.2139/ssrn.3272468*

Kitayama, S., Conway, L. G., III, Pietromonaco, P.R., Park, H., & Plaut, V. C. (2010). Ethos of independence across regions in the United States: The production-adoption model of cultural change. *American Psychologist*, *65*(6), 559-574. https://doi.org/10.1037/a0020277

Kitayama, S., Ishii, K., Imada, T., Takemura, K., & Ramaswamy, J. (2006). Voluntary settlement and the spirit of independence: Evidence from Japan's "northern frontier". *Journal of Personality and Social Psychology, 91*(3), 369–384. https://doi.org/10.1037/0022-3514.91.3.369

Lorah, J. (2018). Effect size measures for multilevel models: definition, interpretation, and TIMSS example. *Large-Scale Assessments in Education, 6*, 8, https://doi.org/10.1186/s40536-018-0061-2

Ludeke, S. G., Klitgaard, C. N., & Vitriol, J. (2018). Comprehensively-measured authoritarianism does predict vote choice: The importance of authoritarianism's facets, ideological sorting, and the particular candidate. *Personality and Individual Differences*, *123*, 209-216. https://doi.org/10.1016/j.paid.2017.11.019

Malka, A., Lelkes, Y., Bakker, B. N., & Spivack, E. (2020). Who is open to authoritarian governance within western democracies? *Perspectives on Politics*, 1-20. https://doi.org/10.1017/s1537592720002091

Malka, A., Lelkes, Y., & Soto, C. J. (2017). Are cultural and economic conservatism positively correlated? A large-scale cross-national test. *British Journal of Political Science*, *49*(3), 1045-1069. https://doi.org/10.1017/s0007123417000072

Malka, A., Lelkes, Y., & Holzer, N. (2017). Rethinking the rigidity of the right model: Three suboptimal methodological practices and their implications. In J. T. Crawford, & L. Jussim (Eds.) *Frontiers of social psychology: Politics of social psychology* (pp. 116-135). Psychology Press.

Manson, J. H. (2020). Right-wing Authoritarianism, Left-wing Authoritarianism, and pandemic-mitigation authoritarianism. *Personality and individual differences*, *167*, 110251.

McConahay, J. B. (1986). Modern racism, ambivalence, and the modern racism scale. In J. F. Dovidio & S. L. Gaertner (Eds.), *Prejudice, discrimination and racism* (pp. 91-126). Academic Press.

McFarland, S.G., Ageyev, V.S., Abalakina-Paap, M.A. (1992). Authoritarianism in the former Soviet Union. *Journal of Personality and Social Psychology, 63*(6), 1004–1010. https://doi.org/10.1037/0022-3514.63.6.1004

McFarland, S.G., Ageyev, V.S., Abalakina-Paap, M.A. (1993). The authoritarian personality in the United States and the former Soviet Union: Comparative studies. In W.F. Stone, G. Lederer, R. Christie(Eds.), *Strength and Weakness: The Authoritarian Personality Today* (pp. 199-225). Springler Verlag.

McFarland, S. G., Ageyev, V. S., & Djintcharadze, N. (1996). Russian authoritarianism two years after communism. *Personality and Social Psychology Bulletin, 22*(2), 210-217. https://doi.org/10.1177/0146167296222010

Miller, S. V. (2017). Economic threats or societal turmoil? Understanding p References for authoritarian political systems. *Political Behavior*, *39*(2), 457-78. https://doi.org/10.1007/s11109-016-9363-7

Mullen, E., Bauman, C. W., & Skitka, L. J. (2003). Avoiding the pitfalls of politicized psychology. *Analyses of Social Issues and Public Policy*, *3*(1), 171-176. https://doi.org/10.1111/j.1530-2415.2003.00021.x

Murray, D. R., & Schaller, M. (2010). Historical prevalence of infectious diseases within 230 geopolitical regions: A tool for investigating origins of culture. *Journal of Cross-Cultural Psychology, 41*(1), 99–108. doi:10.1177/0022022109349510

Napier, J. L., & Jost, J. T. (2008). The “antidemocratic personality” revisited: A cross-national investigation of working-class authoritarianism. *Journal of Social Issues*, *64*(3), 595-617. https://doi.org/10.1002/9781444307337.ch9

Newman, L. S., & Sargent, R. H. (2020). Liberals Report Lower Levels of Attitudinal Ambivalence Than Conservatives. *Social Psychological and Personality Science.* https://doi.org/10.1177/1948550620939798

Newport, F. (2019). Americans' views of Israel remain tied to religious beliefs. *Gallup*. https://news.gallup.com/opinion/polling-matters/247937/americans-views-israel-remain-tied-religious-beliefs.aspx

Nilsson, A., & Jost, J. T. (2020). The authoritarian-conservatism nexus. *Current Opinion in Behavioral Sciences, 34*, 148-154. https://doi.org/10.1016/j.cobeha.2020.03.003

Nosek, B. A. (2005). Moderators of the relationship between implicit and explicit evaluation. *Journal of Experimental Psychology: General*, *134*(4), 565-584. https://doi.org/10.1037/0096-3445.134.4.565

Oishi, S., Yagi, A., Komiya, A., Kohlbacher, F., Kusumi, T., & Ishii, K. (2017). Does a

major earthquake change job preferences and human values? *European Journal of Personality, 31*(3), 258–265. https://doi.org/10.1002%2Fper.2102

Pazhoohi, F., & Kingstone, A. (2021). Associations of political orientation, xenophobia, right-wing authoritarianism, and concern of COVID-19: Cognitive responses to an actual pathogen threat. *Personality and Individual Differences, 182*, 111081.

Pentony, J.F., Peterson, K.S.E., Philips, O., Leong, C., Harper, P., Bakowski, A., Steward, S., Gonzales, R. (2000). A comparison of authoritarianism in the United States, England, and Hungary with selected nonrandom samples. *European Psychologist, 5*(4), 259–268. https://doi.org/10.1027//1016-9040.5.4.259

Preacher, K. J. (2015). Advances in mediation analysis: A survey and synthesis of new developments. *Annual review of psychology*, *66*, 825-52.

Peterson, B. E., & Gerstein, E. D. (2005). Fighting and flying: Archival analysis of threat, authoritarianism, and the North American comic book. *Political Psychology, 26*(6), 887–904. https://doi.org/10.1111%2Fj.1467-9221.2005.00449.x

Proch, J., Elad-Strenger, J., & Kessler, T. (2018). Liberalism and conservatism, for a change!: Rethinking the association between political orientation and relation to societal change. *Political Psychology*, *40*(4), 877-903. https://doi.org/10.1111/pops.12559

R Core Team. (2014). R: A language and environment for statistical computing (Computer software). *R Foundation for Statistical Computing, Vienna, Austria*. http://www.R-project.org/.

Ray, J. J. (1983). Half of all authoritarians are left-wing: A reply to Eysenck and Stone. *Political Psychology, 4*(1), 139–144. https://doi.org/10.2307/3791178

Rokeach, M. (1960). *The open and closed mind: Investigations into the source of beliefs systems and personality systems*. Basic Books.

Roets, A., & Van Hiel, A. (2011). Item selection and validation of a brief, 15-item version of the Need for Closure Scale. *Personality and Individual Differences, 50*, 90-94.

Rokeach, M. (1960). The open and closed mind:

Sargent, R. H., & Newman, L. S. (2020). Conservatism and attitudinal ambivalence: Investigating conflicting findings. *Personality and Individual Differences*. https://doi.org/10.1016/j.paid.2020.109996

Saunders, B., Badaan, V., Hoffarth, M., & Jost, J. (2020, February 27-29). *Spotting the Loch Ness Monster, or smiling for the surgeon's photograph? A critique of Conway and colleagues' (2018) research on left-wing authoritarianism* [Symposium]. Society for Personality and Social Psychology, New Orleans, LA, United States.

Schaller, M., Conway, L. G., III, & Tanchuk, T. (2002). Selective pressures on the once and future contents of ethnic stereotypes: Effects of the 'communicability' of traits. *Journal of Personality and Social Psychology, 82*(6), 861-877. https://doi.org/10.1037/0022-3514.82.6.861

Shils, E. A. (1954). Authoritarianism: Right and left. In Christie, R., & Jahoda, M. (Eds.),*Studies in the Scope and the Method of the Authoritarian Personality* (pp. 24-29). Free Press.

Sprong, S., Jetten, J., Wang, Z., Peters, K., Mols, F., Verkuyten, M., Bastian, B.,

Ariyanto, A., Autin, F., Ayub, N., Badea, C., Besta, T., Butera, F., Costa-Lopes, R., Cui, L., Fantini, C., Finchilescu, G., Gaertner, L., Gollwitzer, M., … Wohl, M. J. A. (2019). "Our Country Needs a Strong Leader Right Now": Economic Inequality Enhances the Wish for a Strong Leader. *Psychological Science, 30*(11), 1625–1637. https://doi.org/10.1177/0956797619875472

Stone, W. F. (1980). The myth of left-wing authoritarianism. *Political Psychology, 2*(3/4), 3-19. https://doi.org/10.2307/3790998

Terrizzi, J. A., Shook, N. J., & McDaniel, M. A. (2013). The behavioral immune system and social conservatism: A meta-analysis. *Evolution and Human Behavior, 34*(2), 99–108. https://doi.org/10.1016%2Fj.evolhumbehav.2012.10.003

Tetlock, P. E. (1994). Political psychology or politicized psychology: Is the road to scientific hell paved with good moral intentions? *Political Psychology, 15*(3), 509-529. https://doi.org/10.2307%2F3791569

Tetlock, P. E., Armor, D., Peterson, R.S. (1994). The slavery debate in antebellum America: Cognitive style, value conflict, and the limits of compromise. *Journal of Personality and Social Psychology, 66*(1), 115-126. https://doi.org/10.1037/0022-3514.66.1.115

Thomas, K. R. (2013). Wild analysis in politics. *Political Psychology, 34*(6), 927–934. https://doi.org/10.1111/pops.12090

Todosijević, B. (2005). Authoritarianism and socialist ideology: The case of Yugoslavia, 1995. In R.F. Farnen, H. De Dekker, C. Landtsheer, H. Sünker, D.B. German (Eds.), *Democratization, Europeanization, and Globalization Trends: Cross-*

national Analysis of Authoritarianism, Socialization, Communications, Youth, and Social Policy. Peter Lang.

Todosijević, B., & Enyedi, Z. (2008). Authoritarianism without dominant ideology: Political manifestations of authoritarian attitudes in Hungary. *Political Psychology*, *29*(5), 767-787. https://doi.org/10.1111/j.1467-9221.2008.00663.x

Van de Vliert, E. (2013). Climato-economic habitats support patterns of human needs, stresses, and freedoms. *Behavioral and Brain Sciences, 36*(5), 465–480. https://doi.org/10.1017%2Fs0140525x12002828

Van de Vliert, E., & Conway, L. G. III. (2019). Northerners and Southerners differ in conflict culture. *Negotiation and Conflict Management Research, 12(3),* 256-277. DOI: 10.1111/ncmr.12138.

Van Hiel, A., Duriez, B., Kossowska, M. (2006). The presence of left-wing authoritarianism in Western Europe and its relationship with conservative ideology. *Political Psychology, 27*(5), 769-793. https://doi.org/10.1111/j.1467-9221.2006.00532.x

Vasilopoulos, P., Marcus, G. E., & Foucault, M. (2018). Emotional responses to the Charlie Hebdo Attacks: Addressing the authoritarianism puzzle. *Political Psychology, 39*(3), 557–575. https://doi.org/10.1111%2Fpops.12439

Wronski, J. Bankert, A., Amira, K., Johnson, A.A., & Levitan, L.C. (2018). A tale of two Democrats: How authoritarianism divides the Democratic Party. *The Journal of Politics, 80(4),* 1384-1388. https://doi.org/10.1086/699338

Zakrisson, I. (2005). Construction of a short version of the right-wing authoritarianism (RWA) scale. *Personality and Individual Differences, 39,* 863–872.
